# Cyanamide-induced desulfhydration enables access to α,β-dehydropeptides

**DOI:** 10.1021/acs.orglett.6c03312

**Published:** 2026-09-11

**Authors:** Yi Yang, Songyi Xue, Ryan Moreira, Wilfred A. van der Donk

**Affiliations:** Department of Chemistry and Howard Hughes Medical Institute, University of Illinois at Urbana-Champaign, Urbana, IL, 61822, USA

## Abstract

Dehydroamino acids are found in natural products, disease-associated proteins, and are used for the diversification of proteins and peptides. The synthesis of peptide sequences containing dehydroamino acids remains non-trivial and only a few chemical methods allow for their incorporation. We report the conversion of amino acid residues bearing β-thiols into dehydroamino acids through the treatment of unprotected peptides and proteins with cyanamide under mild, fully aqueous conditions that are compatible with commonly used buffer salts.

## Graphical Abstract

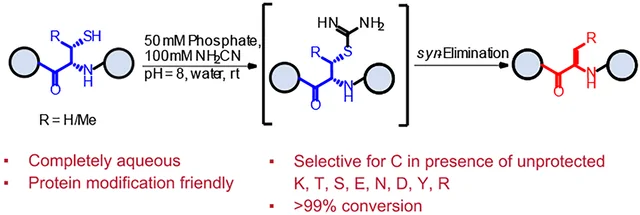

The dehydroaminoacid (DhAA) posttranslational modification is featured prominently in natural products and human disease.^1, 2^ In natural products biosynthesis, DhAAs are reactive intermediates that facilitate peptide cyclisation through cycloaddition or Michael addition.^3, 4^ For example, in the biosynthesis of lanthipeptides, lanthionine synthetases catalyze the dehydration of Ser/Thr residues to dehydroalanine (Dha) and dehydrobutyrine (Dhb), respectively, which serve as electrophiles for the enzyme-catalyzed Michael-type addition of Cys side chains (Scheme 1).^3^ DhAAs are also found in mature natural products where they can modulate peptide conformation,^5, 6^ increase proteolytic stability,^7^ and can serve as electrophilic warheads.^8^ DhAAs are rarely found in the human proteome, but are implicated in HIV-1 and Alzheimer's disease (AD),^2, 9^ with Dha and Dhb residues detected in protein fragments isolated from brain tissue of patients with AD.^2^ DhAAs are also important intermediates in the chemical modification of recombinantly expressed proteins^10^ because they provide sites for diversification s through downstream functionalizations.^11^ Several chemical methods for converting Cys to Dha in large proteins have been reported.^12^ These include oxidative elimination and bis-alkylation of Cys residues followed by elimination which have proven utility but suffer from some limitations (Scheme 1).^13, 14^ The oxidative elimination chemistry often proceeds with competing disulfide formation.^14^ The bis-alkylation elimination chemistry is more reliable, but often requires using an organic cosolvent and can proceed with formation of stapled peptides when multiple Cys are present even with optimized reagents.^15^

Existing methods for installing DhAAs into synthetic peptides also have limitations.^16, 17^ The linear chemical synthesis of dehydropeptides is complicated by difficulties associated with coupling onto their α-amino group because of tautomerization to the imine and hydrolysis.^17^ Moreover, the electrophilicity of DhAAs, especially Dha, results in the formation of adducts with basic reagents during peptide synthesis.^18^ Residues containing substituents at the β-position, such as Dhb, are also configurationally unstable, rendering thermodynamically disfavored isomers such as *E*-Dhb^1^ residues a challenge.^19^ In the realm of synthetic chemistry, β-selenium-containing residues and α-azido protected DhAAs are the most reliable synthons for the installation of DhAAs with stereospecificity.^20–22^ However, β-selenium-containing residues undergo elimination under conditions typically used for peptide synthesis,^23^ and traceless Staudinger ligations onto α-azido-protected DhAAs proceed slowly and with competing reduction.^21^ For these reasons, some studies have opted for installation of DhAAs late-stage via the bis-alkylation elimination of Cys and other β-thiol-containing amino acids,^24, 25^ or by fragment coupling.^26, 27^

Inspired by previous reports, we hypothesized that isothioureas formed by the transfer of an amidine to a β-thiol of an amino acid would undergo a *syn*-elimination yielding DhAAs (Scheme 1).^6, 28, 29^ To test the hypothesis, analogs of a fragment of the *Streptomyces tendae* Tü901 lanthipeptide SapT that contained L-*erythro*-β- or L-*threo*-β-methylcysteine (L-*erythro*-MeCys or L-*threo*-MeCys) (**1** or **2**) were synthesized.^30^ Peptide **1** was incubated overnight at room temperature in phosphate buffer (50 mM, pH = 8.0) that contained an electrophilic form of amidine (100 mM). The crude reactions were evaluated by liquid chromatography-mass spectrometry (LCMS). Of the water-soluble reagents tested, cyanamide was the only one that completely converted **1** to Dhb-containing peptide **3** (Tables S1 and S2; Figure S1). The effects of buffer salt and pH on the conversion of **1** to **3** by cyanamide were tested. No conversion was observed in CH_3_COONa/CH_3_COOH buffer at pH 5 (Table S2). At pH 8, reactions buffered with tris(hydroxymethyl)aminomethane (tris), 4-(2-hydroxyethyl)-1-piperazineethanesulfonic acid (HEPES), phosphate (PB), or tricine proceeded to completion (Table S2). To evaluate the stereoselectivity of the reaction of **1** with cyanamide/phosphate buffer, a standard mixture of **3** and **4** was prepared using the bis-alkylation elimination method.^11, 15^ This standard was analyzed by NMR spectroscopy. ^1^H NMR spectra showed two peaks at 6.39 ppm and 5.65 ppm that were consistent with the β-H of *Z*- and *E*-Dhb, respectively (Figure S2).^30^ The assignment was based on a cross-peak in the 2D NOESY spectrum indicating that the α-NH and vinyl proton were on the same side of the double bond in the spin system containing the peak at 5.65 ppm consistent with the *E* configuration (Figure S3).^20^ Reaction of **1** with cyanamide/phosphate buffer produced exclusively *Z*-Dhb-containing peptide **3** (Table 1). For comparison with the bis-alkylation elimination method, **1** and **2** were treated with methyl 2,5-dibromopentanoate (MDBP)/K_2_CO_3_ in 1:1 DMSO:H_2_O (Table 1).^25^ Analysis of the product by ^1^H NMR spectroscopy revealed that they both gave the same mixture of products in a 1:8 *E*:*Z* ratio. These results suggest that the bis-alkylation elimination chemistry is not stereospecific and that the cyanamide elimination process that gave a higher selectivity for **3** might be stereospecific. We attempted to verify this conclusion but unfortunately none of the tested reagents efficiently converted L-*threo*-MeCys-containing peptide **2** to Dhb-containing peptide **3** or **4**. LCMS analysis of a sample of **2** treated with cyanamide/phosphate buffer revealed that the starting material was consumed and converted to unspecified products; trace quantities of **4** were detected (Figure S4). These results suggest that elimination of the isothiourea is unfavorable when the β-Me and α-carbonyl are on the same side of the incipient double bond in the transition state due to increased 1,3-allylic strain.^19^

Recently, Dha-containing sequences derived from hyperphosphorylated tau and adducts thereof were found to be enriched in samples of protein aggregate harvested from brain of AD patients compared.^2^ LanCLs are human proteins that catalyze the addition of glutathione (GSH) to Dha, possibly including disease-associated proteins containing Dha.^2, 13^ In an effort to follow up on these findings, two sequences associated with abnormal crosslinking of tau (peptides **5** and **6**) were synthesized replacing the Ser with Cys (Table S3).^2^ Both peptides were treated with cyanamide/phosphate buffer at room temperature overnight. Analysis by ^1^H NMR showed that Dha-containing peptides **7** and **8** (Figure 1) were formed with few side products or impurities (Figures S5 and S6). To compare these results to the bis-alkylation-elimination method, peptide **5** was dissolved in 1:1 DMSO:H_2_O and treated with MDBP/K_2_CO_3_ for 4 h at 37 °C. Both conditions gave samples of **7** that were similar in quality and yield (Figure S5 and S7). This finding demonstrated that cyanamide/phosphate buffer is a promising alternative to existing methods for converting Cys into Dha that does not require a co-solvent.

To further probe the scope of the method, substrates **9**-**12** were treated with cyanamide/phosphate buffer and analyzed by LCMS (Figure 1 and Table S3). Peptides **9**-**11** possessed one Cys and were converted to Dha-containing peptides **14–16**. Peptide **11** was converted into a stoichiometric mixture of dehydropeptide **16** and isothiourea adduct **13** (Figure S8). High-resolution MS-MS analysis confirmed the formation of dehydropeptide **16** (Figure S8), and analysis of **13**, which possessed an exact mass consistent with a isothiourea-containing peptide, induced the loss of a fragment that matched the expected mass of thiourea. Further fragmentation of **13** produced a pattern that matched that of dehydropeptide **16**, suggesting that the isothiourea-containing peptide was converted to **16** during the ionization and fragmentation process (Figure S8). These results are consistent with the elimination process proceeding through an isothiourea-containing peptide intermediate. The scope of this reaction is broad, and as demonstrated with substrates **5**, **6**, **9**-**12** it is selective for Cys in the presence of unprotected Lys, Thr, Ser, Glu, Gln, Asp, Tyr, and Arg side chains (Figures S9–S14). Substrate **12** possessed two Cys residues and underwent complete desulfhydration yielding peptide **17** possessing two Dha residues. Cyanamide did not react with the N- or C-termini of unprotected peptides and crosslinked byproducts were not observed when two Cys were present in the substrate.

To determine the generality of the reaction proceeding through an isothiourea intermediate, crude reaction mixtures were screened for masses for isothiourea adducts via LCMS. Peptides **9** and **10** also yielded detectable quantities of isothiourea-containing peptides **18** and **19** (Table S3, Figures S11, S12, and S15).

The chemical synthesis of natural products is facilitated by methods for making dehydropeptides that are compatible with Cys protecting groups that are orthogonal to Fmoc/*tert*-Bu/Trt, such as the Acm group.^31^ To test the compatibility of the cyanamide method with the Acm group, peptide **20** was synthesized, which contained an unprotected Cys side chain after deprotection of a trityl group and a second Cys residue protected with the Acm group. Treatment of peptide **20** with cyanamide/phosphate buffer resulted in complete conversion and formation of peptide **21** (Figure S16). MS-MS sequencing confirmed that the Acm protecting group was intact.

Some natural products contain dehydroamino acids beyond Dha and Dhb. For example, yaku’amide A contains dehydrovaline and dehydroisoleucine,^32^ with the latter also found in the mycotoxin phomopsin.^33^ Cyanamide/phosphate buffer could provide access to dehydrovaline containing peptides from peptide sequences containing the commercially available penicillamine. To test this possibility, peptide **22** was synthesized and incubated with cyanamide/phosphate buffer overnight at room temperature. LCMS analysis showed insignificant conversion of peptide **22**; traces of an exact mass consistent with dehydrovaline-containing peptide **23** were detected (Figure S17). The method in its current state is therefore not useful for the synthesis of dehydrovaline-containing peptides.

The ability of cyanamide/phosphate buffer to convert a Cys to a Dha in a recombinantly expressed natural product was tested next. Bibacillin 1 is a recently discovered two-component lantibiotic.^34^ Bib1α and Bib1β comprising the mature lantibiotic are biosynthesized from the ribosomally synthesized proteins Bib1 A1 and A2. Previous studies revealed that an analog of Bib1β that contained a Dha in place of a Ser at position 16 (Bib1β_Dha_) possessed favorable bioactive properties including the ability to stimulate non-cognate quorum sensing systems.^34^ This analog could not be accessed biosynthetically and therefore a chemoenzymatic route was pursued in which Ser64 in Bib1 A2 was mutated to Cys allowing for conversion to Dha using bis-alkylation elimination after recombinant expression.^34^ This reaction was repeated in the current study using both cyanamide/phosphate buffer and bis-alkylation elimination conditions. Both reactions gave Bib1 A2 Ser64Dha; however, the reaction employing cyanamide/phosphate buffer proceeded with less byproducts (Figure 2). The product was treated with the protease CylA, and analyzed by high resolution MS-MS. The fragmentation pattern was consistent with previously data^34^ showing Cys64 was converted to Dha (Figure S18).

In conclusion, the treatment of peptides containing β-thiol amino acid residues with cyanamide in aqueous buffer results in desulfhydration and yields dehydropeptides. The reaction does not require organic cosolvents, is compatible with common buffers, reacts specifically with Cys in the presence of other reactive side chains, and proceeds under mildly basic conditions at room temperature. These attributes make cyanamide an appealing reagent for incorporating Dha into proteins under native conditions that are then handles for bioconjugation,^11, 12, 35^ and for synthesis of Dha-containing natural products such as lanthipeptides.^10, 26, 27, 36–41^ Mechanistic investigations suggest that the reaction proceeds through an isothiourea that eliminates under mildly basic conditions (Scheme 2). Experiments on a peptide containing L-*erythro*-MeCys demonstrated that this process likely proceeds through a *syn*-elimination mechanism analogous to a Chugaev elimination (Scheme 2).^42^ Such a mechanism could also explain why L-*threo-*MeCys did not undergo elimination to give *E*-Dhb, since a *syn* elimination would result in increased steric repulsion between the β-Me group and the C-terminal amide carbonyl (Scheme 2). This mechanism circumvents the formation of unwanted byproducts, such as stapled peptides and intermolecular disulfides, which can complicate other methods for the incorporation of DhAAs.^43^

## Supplementary Material

SI

The Supporting Information is available free of charge on the ACS Publications website.

Experimental procedures, pH and reagent screening, NMR spectra, LCMS data and mass spectra (PDF).

## Figures and Tables

**Figure 1.**
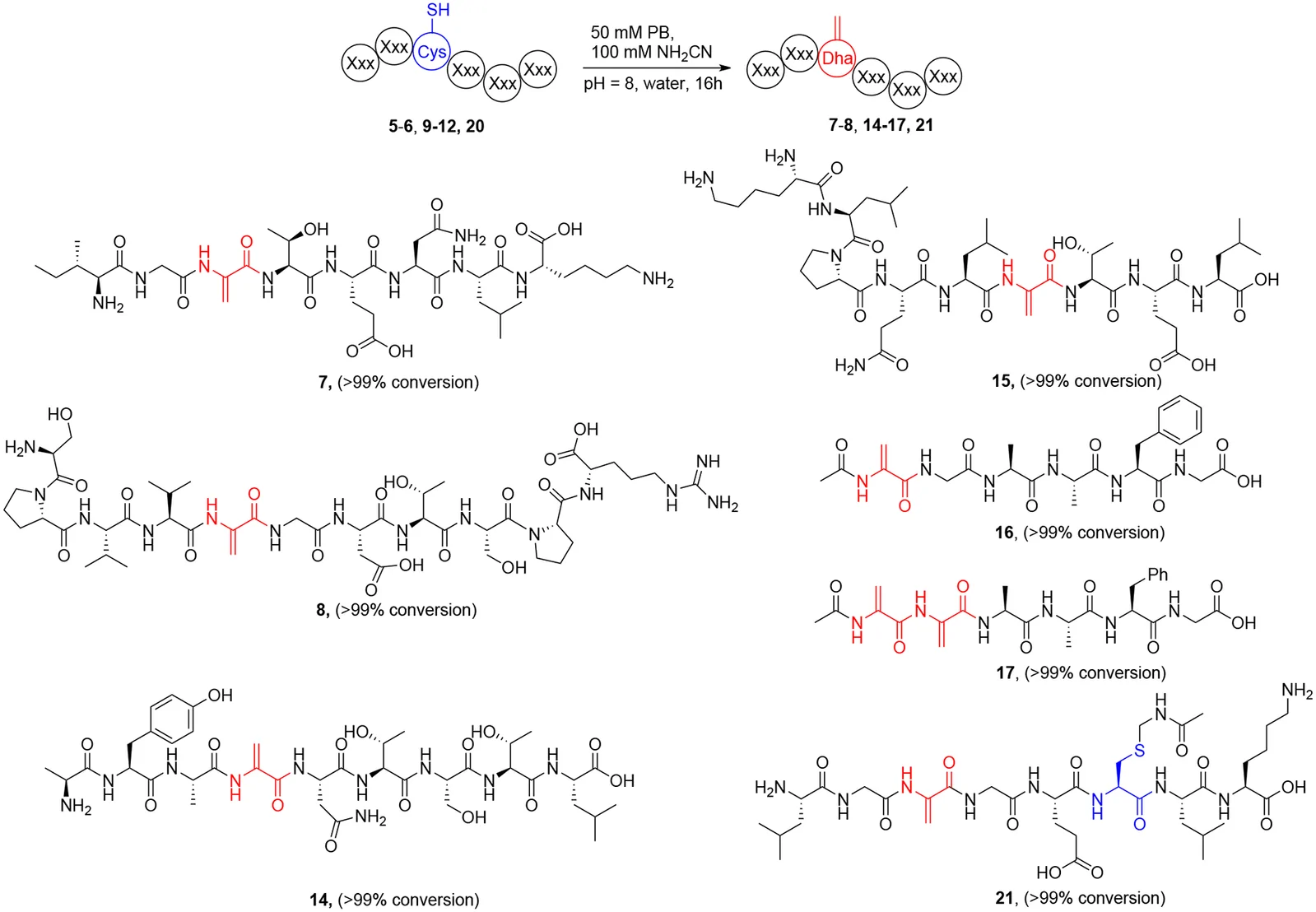
Major products from treating peptides 5–6, 9–12, 20 with cyanamide in phosphate buffer at pH 8.

**Figure 2.**
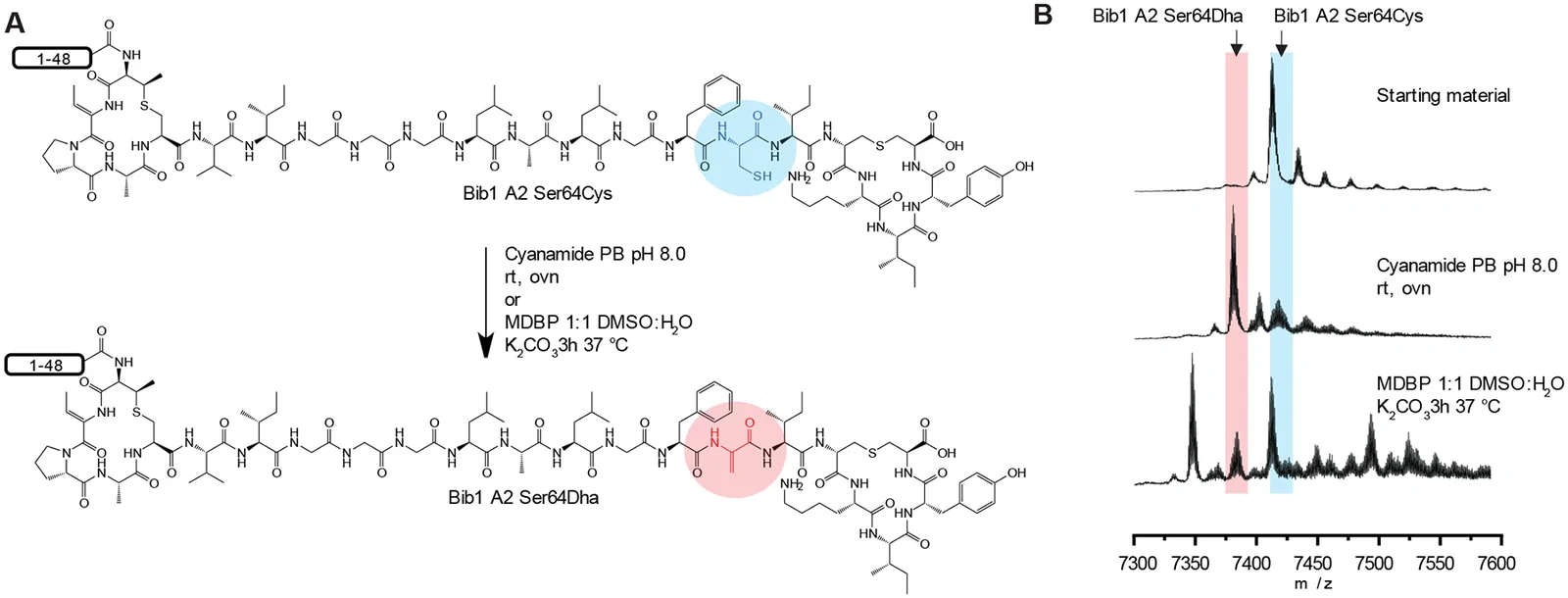
Conversion of Cys to Dha in a recombinantly expressed protein. A) Conversion of Bib1 A2 Ser6Cys to Bib1 A2 Ser64Dha. B) MALDI-ToF spectra of Bib1 A2 Ser64Cys before and after treatment with cyanamide/phosphate buffer or MDBP/K_2_CO_3_.

**Scheme 1.**
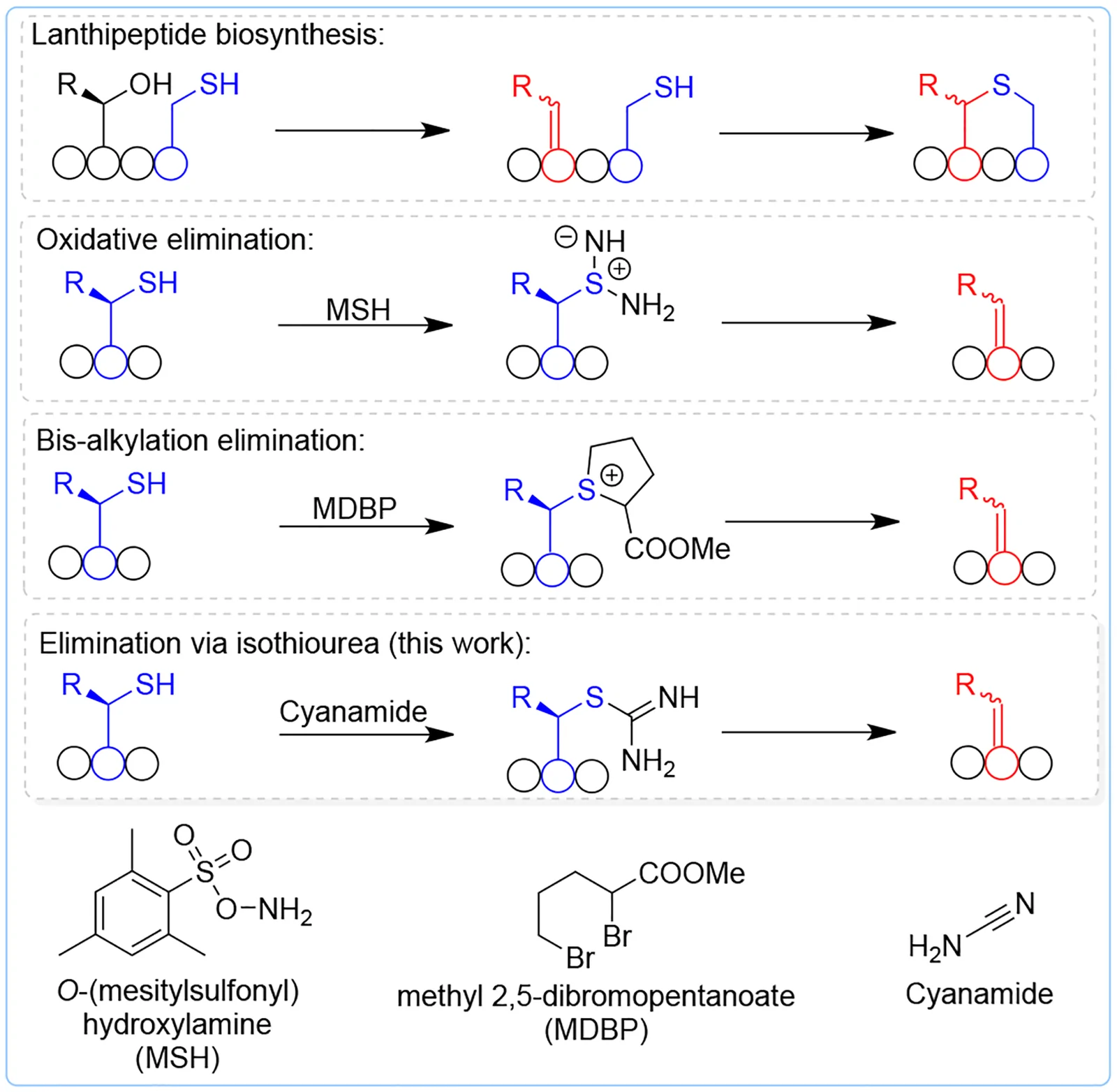
Synthesis and biosynthesis of DhAAs in peptides.

**Scheme 2.**
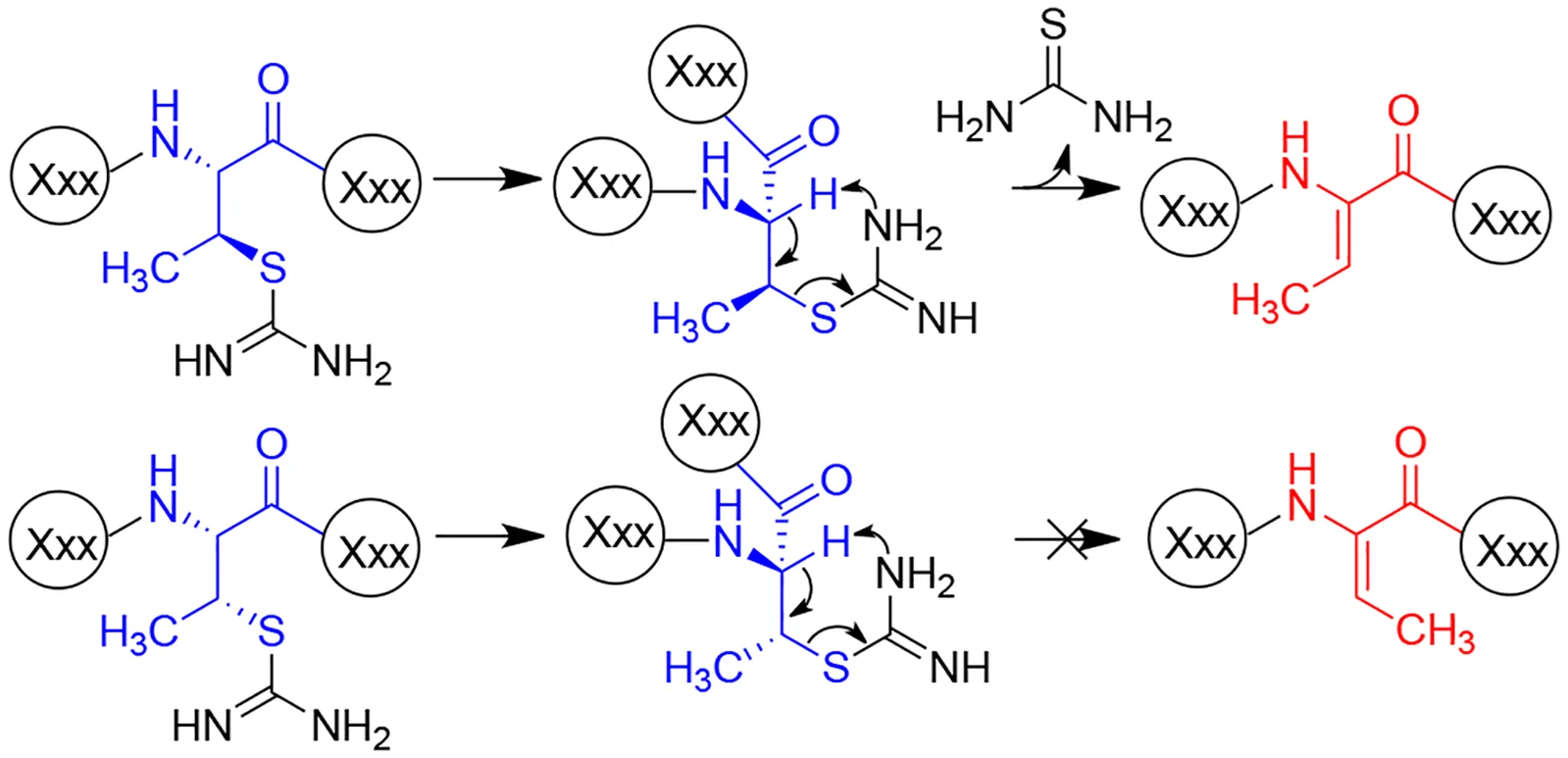
Proposed mechanism of cyanamide-induced desulfhydration of a peptide containing L-*erythro*- or L-*threo*-MeCys. Xxx represents various amino acids.

**Table 1.** The stereoselectivity of the elimination of MeCys- containing peptides facilitated by cyanamide and MDBP.

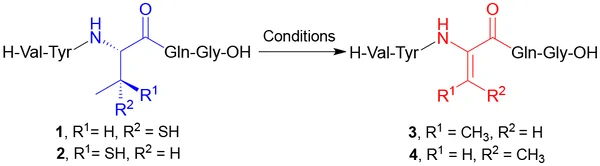			
Starting Material^b^	Conditions^c^	4:3^a^	Conversion^d^
**1**	Phosphate buffer (pH 8.0), Cyanamide, rt, 12 h	1:>9^e^	>99%
**1**	DMSO/H_2_O, MDBP, K_2_CO_3_, 3h, 37 °C	1:8	>99%
**2**	DMSO/H_2_O, MDBP, K_2_CO_3,_ 3h, 37 °C	1:8	>99%

a Determined by ^1^H NMR.

b Starting material at 1 mM.

c pH determined after adding cyanamide.

d Conversion was evaluated by LCMS.

e The concentration of **4** was below the limit of detection.

## Data Availability

The data underlying this study are available in the published article, in its Supporting Information, and openly available in Mendeley Data at https://data.mendeley.com/datasets/7kj9n9syfk/1

## References

[1] Wang S; Wu K; Tang YJ; Deng H. Dehydroamino acid residues in bioactive natural products. Nat. Prod. Rep. 2024, 41 (2), 273–297. DOI: 10.1039/d3np00041a. 37942836 PMC10880069

[2] Markovich SW; Frey BL; Scalf M; Shortreed MR; Smith LM. Dehydroamino acids and their crosslinks in Alzheimer’s disease aggregates. Brain Commun. 2025, 7 (1), fcaf019. DOI: 10.1093/braincomms/fcaf019 (acccessed 12/19/2025). 39882022 PMC11775630

[3] Repka LM; Chekan JR; Nair SK; van der Donk WA. Mechanistic understanding of lanthipeptide biosynthetic enzymes. Chem. Rev. 2017, 117, 5457–5520. DOI: 10.1021/acs.chemrev.6b00591. 28135077 PMC5408752

[4] Nguyen DT; Le TT; Rice AJ; Hudson GA; van der Donk WA; Mitchell DA. Accessing diverse pyridine-based macrocyclic peptides by a two-site recognition pathway. J. Am. Chem. Soc. 2022, 144 (25), 11263–11269. DOI: 10.1021/jacs.2c02824. 35713415 PMC9247985

[5] Tang W; van der Donk WA. The sequence of the enterococcal cytolysin imparts unusual lanthionine stereochemistry. Nat. Chem. Biol. 2013, 9 (3), 157–159. DOI: 10.1038/nchembio.1162. 23314913 PMC3578037

[6] Jones LH. Dehydroamino acid chemical biology: an example of functional group interconversion on proteins. RSC Chem. Biol. 2020, 1 (5), 298–304. DOI: 10.1039/d0cb00174k. 34458767 PMC8341704

[7] Jalan A; Kastner DW; Webber KGI; Smith MS; Price JL; Castle SL. Bulky dehydroamino acids enhance proteolytic stability and folding in β-hairpin peptides. Org. Lett. 2017, 19 (19), 5190–5193. DOI: 10.1021/acs.orglett.7b02455. 28910115 PMC6085080

[8] MacKintosh C; Beattie KA; Klumpp S; Cohen P; Codd GA. Cyanobacterial microcystin-LR is a potent and specific inhibitor of protein phosphatases 1 and 2A from both mammals and higher plants. FEBS Lett 1990, 264 (2), 187–192. 2162782 10.1016/0014-5793(90)80245-e

[9] Miller RM; Knoener RA; Benner BE; Frey BL; Scalf M; Shortreed MR; Sherer NM; Smith LM. Discovery of dehydroamino acid residues in the capsid and matrix structural proteins of HIV-1. J. Proteome Res. 2022, 21 (4), 993–1001. DOI: 10.1021/acs.jproteome.1c00867. 35192358 PMC8976760

[10] Josephson B; Fehl C; Isenegger PG; Nadal S; Wright TH; Poh AWJ; Bower BJ; Giltrap AM; Chen L; Batchelor-McAuley C; Light-driven post-translational installation of reactive protein side chains. Nature 2020, 585 (7826), 530–537. DOI: 10.1038/s41586-020-2733-7. 32968259

[11] Dadová J; Galan SRG; Davis BG. Synthesis of modified proteins via functionalization of dehydroalanine. Curr. Opin. Chem. Biol. 2018, 46, 71–81. DOI: 10.1016/j.cbpa.2018.05.022. 29913421

[12] Chalker JM; Gunnoo SB; Boutureira O; Gerstberger SC; Fernández-González M; Bernardes GJL; Griffin L; Hailu H; Schofield CJ; Davis BG. Methods for converting cysteine to dehydroalanine on peptides and proteins. Chem. Sci. 2011, 2 (9), 1666–1676. DOI: 10.1039/C1SC00185J.

[13] Lai KY; Galan SRG; Zeng Y; Zhou TH; He C; Raj R; Riedl J; Liu S; Chooi KP; Garg N; LanCLs add glutathione to dehydroamino acids generated at phosphorylated sites in the proteome. Cell 2021, 184 (10), 2680–2695. DOI: 10.1016/j.cell.2021.04.001. 33932340 PMC8209957

[14] Bernardes GJ; Chalker JM; Errey JC; Davis BG. Facile conversion of cysteine and alkyl cysteines to dehydroalanine on protein surfaces: versatile and switchable access to functionalized proteins. J. Am. Chem. Soc. 2008, 130 (15), 5052–5053. DOI: 10.1021/ja800800p. 18357986

[15] Morrison PM; Foley PJ; Warriner SL; Webb ME. Chemical generation and modification of peptides containing multiple dehydroalanines. Chem. Commun. 2015, 51 (70), 13470–13473. DOI: 10.1039/C5CC05469A. PMC4847484 26219458

[16] Mori T; Sumida S; Sakata K; Shirakawa S. Efficient synthetic methods for α,β-dehydroamino acids as useful and environmentally benign building blocks in biological and materials science. Org. Biomol. Chem. 2024, 22 (23), 4625–4636. DOI: 10.1039/D4OB00507D. 38804977

[17] Bonauer C; Walenczyk T; König B. a,b-Dehydroamino Acids. Synthesis 2006, 1–20.

[18] Mthembu SN; Chakraborty A; Schönleber R; Albericio F; de la Torre BG. Solid-phase synthesis of C-terminus cysteine peptide acids. Org. Process Res. Dev. 2022, 26 (12), 3323–3335. DOI: 10.1021/acs.oprd.2c00321.

[19] Yamashita T; Kuranaga T; Inoue M. Solid-phase total synthesis of bogorol a: stereocontrolled construction of thermodynamically unfavored (E)-2-amino-2-butenamide. Org. Lett. 2015, 17 (9), 2170–2173. DOI: 10.1021/acs.orglett.5b00769. 25866994

[20] Lambrecht MJ; Lu Y; Alexander MK; Burdick DJ; Cheng C; Lai T; Rutherford ST; Wang Q; Xu J; Koehler MFT. Total synthesis of pedopeptin C. Org. Lett. 2025, 27 (25), 6767–6771. DOI: 10.1021/acs.orglett.5c01928. 40512973

[21] Itoh H; Miura K; Kamiya K; Yamashita T; Inoue M. Solid-phase total synthesis of yaku'amide B enabled by traceless Staudinger ligation. Angew. Chem. Int. Ed. 2020, 59 (11), 4564–4571. DOI: 10.1002/anie.201916517. 31943639

[22] Zhou H; van der Donk WA. Biomimetic stereoselective formation of methyllanthionine. Org. Lett. 2002, 4 (8), 1335–1338. DOI: 10.1021/ol025629g. 11950356

[23] Levengood MR; van der Donk WA. Dehydroalanine-containing peptides: preparation from phenylselenocysteine and utility in convergent ligation strategies. Nat. Protoc. 2006, 1 (6), 3001–3010. DOI: 10.1038/nprot.2006.470. 17406561

[24] Engelhardt DB; Donnelly BL; Beadle J; van Belkum MJ; Vederas JC. Ring-opening reactions for the solid-phase synthesis of nisin lipopeptide analogues. Org. Biomol. Chem. 2022, 20 (45), 8988–8999. DOI: 10.1039/D2OB01526A. 36326622

[25] Dickman R; Mitchell SA; Figueiredo AM; Hansen DF; Tabor AB. Molecular recognition of lipid II by lantibiotics: synthesis and conformational studies of analogues of nisin and mutacin rings A and B. J. Org. Chem. 2019, 84 (18), 11493–11512. DOI: 10.1021/acs.joc.9b01253. 31464129 PMC6759747

[26] Liu W; Chan ASH; Liu H; Cochrane SA; Vederas JC. Solid supported chemical syntheses of both components of the lantibiotic lacticin 3147. J. Am. Chem. Soc. 2011, 133 (36), 14216–14219. DOI: 10.1021/ja206017p. 21848315

[27] Ross AC; Liu H; Pattabiraman VR; Vederas JC. Synthesis of the lantibiotic lactocin S using peptide cyclizations on solid phase. J. Am. Chem. Soc. 2010, 132 (2), 462–463. DOI: 10.1021/ja9095945. 20017553

[28] Yap SY; Butcher T; Spears RJ; McMahon C; Thanasi IA; Baker JR; Chudasama V. Chemo- and regio-selective differential modification of native cysteines on an antibody via the use of dehydroalanine forming reagents. Chem. Sci. 2024, 15 (22), 8557–8568. DOI: 10.1039/D4SC00392F. 38846383 PMC11151841

[29] Bashore C; Jaishankar P; Skelton NJ; Fuhrmann J; Hearn BR; Liu PS; Renslo AR; Dueber EC. Cyanopyrrolidine inhibitors of ubiquitin specific protease 7 mediate desulfhydration of the active-site cysteine. ACS Chem. Biol. 2020, 15 (6), 1392–1400. DOI: 10.1021/acschembio.0c00031. 32302100

[30] Sarksian R; Zhu L; van der Donk WA. syn-Elimination of glutamylated threonine in lanthipeptide biosynthesis. Chem. Commun. 2023, 59 (9), 1165–1168. DOI: 10.1039/d2cc06345j. PMC9890492 36625436

[31] Chakraborty A; Mthembu SN; de la Torre BG; Albericio F. Ready to use cysteine thiol protecting groups in SPPS, a practical overview. Org. Process Res. Dev. 2024, 28 (1), 26–45. DOI: 10.1021/acs.oprd.3c00425.

[32] Kuranaga T; Mutoh H; Sesoko Y; Goto T; Matsunaga S; Inoue M. Elucidation and Total Synthesis of the Correct Structures of Tridecapeptides Yaku’amides A and B. Synthesis-Driven Stereochemical Reassignment of Four Amino Acid Residues. J. Am. Chem. Soc. 2015, 137 (29), 9443–9451. DOI: 10.1021/jacs.5b05550. 26146759

[33] Culvenor CCJ; Edgar JA; Mackay MF; P. Gorst-Allman C; F.O. Marasas W; S. Steyn P; Vleggaar R; L. Wessels P. Structure elucidation and absolute configuration of phomopsin a, a hexapeptide mycotoxin produced by phomopsis leptostromiformis. Tetrahedron 1989, 45 (8), 2351–2372. DOI: 10.1016/S0040-4020(01)83436-0.

[34] Moreira R; Yang Y; Luo Y; Gilmore MS; van der Donk WA. Bibacillin 1: a two-component lantibiotic from *Bacillus thuringiensis*. RSC Chem. Biol. 2024, 5, 1060–1073. DOI: 10.1039/d4cb00192c. 39268544 PMC11385697

[35] Wright TH; Bower BJ; Chalker JM; Bernardes GJ; Wiewiora R; Ng WL; Raj R; Faulkner S; Vallee MR; Phanumartwiwath A; Posttranslational mutagenesis: A chemical strategy for exploring protein side-chain diversity. Science 2016, 354 (6312). DOI: 10.1126/science.aag1465. 27708059

[36] Tabor AB. The challenge of the lantibiotics: synthetic approaches to thioether-bridged peptides. Org. Biomol. Chem. 2011, 9 (22), 7606–7628. DOI: 10.1039/c1ob05946g. 21960309

[37] Mukherjee S; Huo L; Thibodeaux GN; van der Donk WA. Synthesis and bioactivity of diastereomers of the virulence lanthipeptide cytolysin. Org. Lett. 2016, 18 (23), 6188–6191. DOI: 10.1021/acs.orglett.6b03246. 27934350 PMC5269379

[38] Knerr PJ; van der Donk WA. Chemical synthesis of the lantibiotic lacticin 481 reveals the importance of lanthionine stereochemistry. J. Am. Chem. Soc. 2013, 135 (19), 7094–7097. DOI: 10.1021/ja4014024. 23621626 PMC3736828

[39] Narayan RS; Vannieuwenhze MS. Versatile and stereoselective syntheses of orthogonally protected beta-methylcysteine and beta-methyllanthionine. Org. Lett. 2005, 7 (13), 2655–2658. DOI: 10.1021/ol0507930. 15957914 PMC1351053

[40] Carrillo AK; Vannieuwenhze MS. Synthesis of the AviMeCys-containing D-ring of mersacidin. Org. Lett. 2012, 14 (4), 1034–1037. 22296295 10.1021/ol2034806 PMC4096711

[41] García-Reynaga P; Carrillo AK; VanNieuwenhze MS. Decarbonylative approach to the synthesis of enamides from amino acids: Stereoselective synthesis of the (*Z*)-aminovinyl-D-cysteine unit of mersacidin. Org. Lett. 2012, 14, 1030–1033. DOI: 10.1021/ol203399x. 22296268 PMC3307523

[42] Bordwell FG; Landis PS. Elimination Reactions. VIII. A trans Chugaev Elimination1. J. Am. Chem. Soc. 1958, 80 (10), 2450–2453. DOI: 10.1021/ja01543a026.

[43] Yang Y; Xue S; Moreira R; van der Donk WA. Cyanamide-induced desulfhydration enables access to α,β-dehydropeptides. ChemRxiv 2026, 2026 (0727). DOI: doi:10.26434/chemrxiv.15004572/v2. PMC13573842 42726266

