# Supplementary material for: Cyanamide-induced desulfhydration enables access to α,β-dehydropeptides
Source: Org Lett. Author manuscript; Available in PMC 2026 Sep 14. (PMC13573842; doi:10.1021/acs.orglett.6c03312)

Cyanamide-Induced desulfhydration enables access to α,β-dehydropeptides

Yi Yang, Songyi Xue, Ryan Moreira* and Wilfred A. van der Donk*

Department of Chemistry and Howard Hughes Medical Institute, University of Illinois at Urbana-Champaign, Urbana, IL, 61822, USA

*corresponding authors' emails: ryanem@illinios.edu, vddonk@illinois.edu

Supporting information

Moreira, Ryan; Yang, Yi; Xue, Songyi; van der Donk, Wilfred (2026), "Data associated with "Cyanamide-induced desulfhydration enables access to α,β-dehydropeptides"", Mendeley Data, V1, doi: 10.17632/7kj9n9syfk.1

General Methods

All reagents used for chemical synthesis were purchased from Sigma Aldrich, Chem-Impex and CombiBlocks. Substrates **9** and **10** were purchased from GenScript and analyzed before use. *N*,*N*-Dimethylformamide (DMF) and dichloromethane (DCM) were dried over 3 Å molecular sieves before use. Chromatography was performed using 60 Å silica gel.

^{1}H NMR spectra were collected at 500 or 600 MHz; ^{13}C NMR spectra were collected at 125 MHz. All spectra were collected on Bruker NMR instruments. The chemical shifts of ^{1}H and ^{13}C resonances were reported relative to the residual solvent peak. The ^{1}H resonances corresponding to residual $CHCl_3$, CH_2Cl_2, DMSO, and 90% H_2O/10% D_2O were assigned chemical shifts of 7.25, 5.32, 2.50, and 4.70 ppm, respectively. The ^{13}C resonances corresponding to $CDCl_3$, CD_2Cl_2, and DMSO-d_6 were assigned chemical shifts equal to 77.0, 53.8, and 39.5 ppm, respectively. Structural assignments were made with additional information from gCOSY, gHSQC, and gHMBC experiments

Matrix-assisted laser desorption/ionization time-of-flight mass spectrometry (MALDI-TOF MS) analysis was performed on a Bruker UltrafleXtreme MALDI-TOF mass spectrometer (Bruker Daltonics) at the University of Illinois School of Chemical Sciences Mass Spectrometry Facility. Super-DHB (Sigma) was used as a matrix.

High resolution LCMS analysis was performed using an Agilent 1290 LC-MS QToF instrument. Data analysis was performed using the MassHunter Qualitative Analysis software (v 10.0) provided by Agilent.

L-Fmoc-*threo*-MeCys-OH (**S1**) was prepared according to a previously described procedure.[1]

Synthetic procedures

Scheme S1. Routes to L-Fmoc-*threo*-β-MeCys(Trt)-OH (**S2**) and L-Fmoc-*erythro*-β-MeCys(Trt)-OH (**S5**).

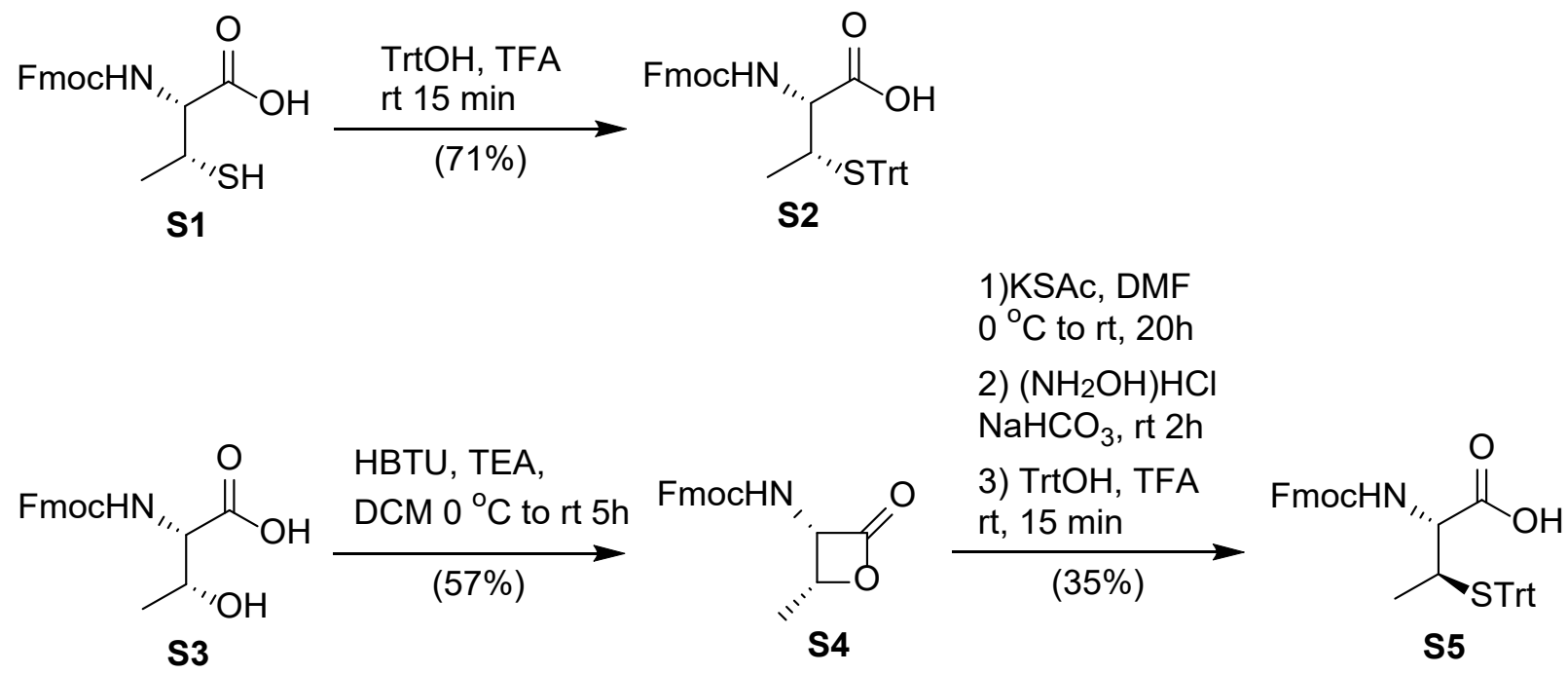

*(2R,3R)-2-((((9H-fluoren-9-yl)methoxy)carbonyl)amino)-3-(tritylthio)butanoic acid (**S2**).*

S1 (1.16 g, 3.26 mmol), prepared as described previously,[1] was dissolved in trifluoroacetic acid (TFA) (6.50 mL) and triphenylmethanol (3.26 mmol, 849 mg, 1.00 equiv.) was added to the solution at room temperature. The solution was stirred for 15 min, then the solvent was removed under reduced pressure and trace TFA was removed by co-evaporation from toluene. The crude residue was purified by silica gel column chromatography (50:50:1 EtOAc:Hexanes:AcOH), which yielded **S2** as a colorless foam (425 mg, 71% yield). ^{1}H and ^{13}C spectra matched previous reports and are provide at the end of the Supporting Information.[2] ^{1}H NMR (500 MHz, CD_2Cl_2): 7.84-7.82 (m, 2H), 7.68-7.65 (m, 2H), 7.56-7.54 (m, 4H), 7.45-7.22 (m, 15H), 5.46 (d, J = 8.8 Hz, 1H), 4.47-4.44 (m, 3H), 4.28 (t, J = 6.9 Hz, 1H), 2.81 (m, 1H), 0.96 (d, J = 6.8 Hz, 3H). ^{13}C NMR (125 MHz, CD_2Cl_2): δ 175.4, 156.5, 144.9, 144.3, 144.1, 141.7, 129.9, 128.3, 128.3, 128.2, 128.1, 127.5, 127.2, 125.5, 120.3, 120.3, 68.2, 67.5, 59.6, 47.5, 42.9, 20.1. HRMS (ESI) m/z: [M − H]− Calcd for C38H32O4NS 598.2052; found 598.2053.

*(9H-fluoren-9-yl)methyl ((2R,3S)-2-methyl-4-oxooxetan-3-yl)carbamate (**S4**).*

Fmoc-L-Thr-OH (**S3**, 5.00 g, 15.5 mmol, obtained from Chem-Impex) was dissolved in dry DCM (150 mL) then cooled in an ice-water bath for 10 min before the addition of 2-(1H-benzotriazol-1-yl)-1,1,3,3-tetramethyluronium hexafluorophosphate (HBTU) (31.0 mmol, 11.8 g, 2.00 equiv.) followed by triethylamine (62.0 mmol, 8.60 mL, 4.00 equiv.). Cooling was removed and the reaction was stirred for 5 h under ambient conditions. The reaction mixture was passed through a short plug of silica and the plug was extracted with DCM until all the product was removed. The product-containing fractions were concentrated and the residue was dissolved in chloroform ($CHCl_3$) and then concentrated until precipitate began to form. Hexanes were added to drive the precipitation to completion (the final ratio of hexanes:$CHCl_3$ was 2:1). The colorless solid was collected and washed with 1:1 hexanes:$CHCl_3$, then residual solvent was removed under reduced pressure. The solid was dissolved in ethyl acetate (300 mL), washed with water (3 × 100 mL), and then brine (1 × 50 mL). The organic layer was dried over $MgSO_4$, then filtered and concentrated.

This procedure yielded **S4** as a colorless solid (2.84 g, 57%). ^{1}H NMR (500 MHz, $CDCl_3$) δ 7.79 (d, *J* = 7.6 Hz, 2H), 7.59 (d, *J* = 7.5 Hz, 2H), 7.38 (dt, *J* = 47.4, 7.5 Hz, 4H), 5.87 (d, *J* = 8.6 Hz, 1H), 5.62 – 5.30 (m, 1H), 4.96 – 4.74 (m, 1H), 4.49 (q, *J* = 8.5 Hz, 2H), 4.22 (t, *J* = 6.8 Hz, 1H), 1.70 – 1.28 (m, 3H). ^{13}C NMR (75 MHz, CD_2Cl_2): δ 169.0, 155.6, 143.7, 143.6, 141.6, 128.1, 127.4, 125.2, 120.3, 75.2, 67.8, 60.6, 47.3, 15.2. HRMS-(ESI) m/z: [M + Na]+ Calcd for C19H17O4NNa 346.1055; found 346.1046. NMR spectra are provided at the end of the Supporting Information.

*(2R,3S)-2-((((9H-fluoren-9-yl)methoxy)carbonyl)amino)-3-(tritylthio)butanoic acid (**S5**).*

S4 (3.63 g, 11.2 mmol. 1.00 equiv.) was dissolved in dry DMF (48.0 mL) and cooled (ice-water). To this solution was added dropwise a solution of potassium thioacetate (1.41 g, 12.3 mmol, 1.10 equiv.) in dry DMF (16.0 mL). Once the addition was complete, cooling was removed and the reaction was stirred for 20 h at room temperature. The reaction was cooled (ice-water) and acidified with 1 M HCl (40 mL). The ice-water bath was removed, and the reaction mixture was extracted with EtOAc (50 mL × 3). The organic layer was dried with $MgSO_4$, passed through a short plug of silica, and then concentrated. The crude residue was dissolved in MeOH (60 mL). Hydroxylamine HCl (834 mg, 12 mmol, 1.07 equiv.) and $NaHCO_3$ (1.50 g, 18.0 mmol, 1.61 equiv.) were added to the stirring solution at room temperature. After 3 h, solvent was removed and the residue was suspended in DCM (50 mL), cooled in an ice-water bath, then acidified with 1 M HCl (50 mL). The layers were separated and the organic layer was dried with $MgSO_4$, filtered, and then concentrated onto silica. The product-containing silica was applied to a short column of silica gel. The plug was washed with DCM then eluted with DCM:MeOH:AcOH (9:1:0.1). The product-containing fraction was collected, concentrated, and then the residue was concentrated from toluene twice before being dissolved in TFA (12.0 mL). Triphenylmethanol (1.56 g, 6.00 mmol, 0.536 equiv.) was added and the solution was stirred for 15 min at room temperature before the solvent was removed under reduced pressure. The residue was concentrated from toluene three times then purified by silica gel column chromatography using EtOAc:hexanes:AcOH (30:70:1). This procedure yielded **S5** as a colorless foam (2.33 g, 35% yield). ^{1}H NMR (500 MHz, CD_2Cl_2) δ 7.80 (d, *J* = 7.6 Hz, 2H), 7.63 (d, *J* = 7.5 Hz, 2H), 7.52 (d, *J* = 7.8 Hz, 6H), 7.41 (t, *J* = 7.5 Hz, 2H), 7.37 – 7.10 (m, 11H), 5.23 (d, *J* = 8.9 Hz, 1H), 4.39 (td, *J* = 11.5, 7.2 Hz, 2H), 4.27 (d, *J* = 7.3 Hz, 1H), 4.02 – 3.85 (m, 1H), 2.99 – 2.76 (m, 1H), 1.09 (d, *J* = 7.3 Hz, 3H). ^{13}C NMR (125 MHz, $CDCl_3$) δ 175.4, 156.3, 144.6, 143.9, 143.7, 141.4, 141.3, 129.6, 128.3, 128.1, 128.1, 128.0, 128.0, 127.8, 127.2, 126.9, 125.3, 125.2, 120.0, 68.2, 67.4, 58.3, 47.2, 41.8, 18.0. HRMS (ESI) m/z: [M − H]− Calcd for C38H32O4NS, 598.2052; found 598.2056.

General procedure for solid-phase peptide synthesis (SPPS)

Fmoc-SPPS was performed using a Liberty Blue™ microwave peptide synthesizer. Commercially available polystyrene resin with a 2-ClTrt linker pre-loaded with Fmoc-Gly (Chem-Impex) was used to make **1**, **2**, **11**, and **12**. TentaGel® resin with a Trt linker (Rapp Polymere) was activated and loaded, as described previously,[3] with L-Fmoc-Lys(Boc)-OH or L-Fmoc-Arg(Pbf)-OH to

initiate the synthesis of **5** or **6**, respectively. All resin loadings were determined using dried resin immediately before peptide synthesis. Syntheses were done on a 0.1 mmol scale and all steps were performed at room temperature unless otherwise noted. Fmoc removal was performed by treating Fmoc-protected peptide on the solid support with 20% piperidine in DMF (3 × 10 min) followed by resin washing with DMF (6 × 1 min). Coupling steps were performed by mixing the resin with a solution of Fmoc-protected amino acid (3 equiv.), ethyl cyanohydroxyiminoacetate (Oxyma) (3 equiv.), and *N*,*N*'-diisopropylcarbodiimide (DIC) (3 equiv.) in DMF for 1 h. The resin was then filtered and washed with DMF (6 × 1 min). The coupling step and Fmoc deprotection was repeated with different Fmoc-protected amino acids until all amino acids were coupled onto the solid supported peptde. When desired, acetylation of the N-terminal amine was performed by treating peptide-bound resin with resin-capping solution (90:8:2 DMF:acetic anhydride:*N*,*N*'-diisopropylethylamine) (2 × 10 min). The resin was washed with DMF (6 × 1 min) and then DCM (6 × 1 min). The resin-bound peptide was removed from the solid support by treatment with a mixture of trifluoroacetic acid (TFA):triisopropylsilane:water (95:2.5:2.5) for 2h. The solid resin was removed by filtration, and the filtrate was concentrated under a stream of nitrogen. Crude peptide was precipitated from the concentrated residue through the addition of ice-cold diethyl ether. The precipitate was collected and analyzed for quality by LCMS. In many cases, further purification was not necessary, and reactions were conducted using crude product. Peptides **20** and **22** were isolated using an Agilent 1260 Infinity II reversed-phase HPLC.

Preparation of a mixture of 3 and 4 by bis-alkylation elimination of 1 or 2

Method 1:

A crude sample of **2** (292 µg, 0.500 µmol, 1.00 equiv.) was dissolved in H_2O deionized using a milliQ setup (MQ H_2O) (0.350 mL). Tris(2-carboxyethyl)phosphine hydrochloride (71.5 µg, 0.250 µmol, 0.500 equiv.) dissolved in MQ H_2O (50.0 µL) was added, and the solution was mixed at room temperature for 45 min. An aqueous solution of sodium phosphate buffer (50.0 µL; 500 mM phosphate concentration) with a pH equal to 8 was added followed by methyl 2,5-dibromopentanoate (4.71 mg, 17.2 µmol, 10.0 equiv) as a solution in DMF (50.0 µL). The mixture was incubated at room temperature for 16 h.

Method 2:

A crude sample of **1** or **2** was dissolved in MQ H_2O (3.43 mM). Tris(2-carboxyethyl)phosphine hydrochloride (0.500 equiv.) was added, and the solution was mixed at room temperature for 30 min before adding an equal volume of methyl 2,5-dibromopentanoate (60.0 equiv.) dissolved in DMSO followed by solid K_2CO_3 (150 equiv.). The mixture was incubated at 37 °C for 3 h with shaking.

*Purification of **3** and **4**:*

Crude products were isolated by semi-preparative reversed-phase HPLC (Macherey-Nagel C18 HTec column (5 µm, 110 Å, 250 x 10 mm); gradient: water with 0.1% TFA (Solvent A): acetonitrile with 0.1% TFA (Solvent B) 98:2 for 5 min, then a linear gradient from 98:2 to 37:63 Solvent A:Solvent B over 20 min; flow rate = 4 mL/min; l = 220 nm; t_R 17.5 min. Product-containing fractions were combined and lyophilized prior to analysis.

General procedure for the desulfhydration of thiol-containing peptides with cyanamide

A solution containing peptide substrate (1.00 equiv.), phosphate buffer (50.0 equiv., pH 8.0) and cyanamide (100 equiv.) was prepared in MQ H_2O (1.00 mM). The pH of the solution was adjusted to 8.0 with NaOH, and the reaction mixture was incubated at room temperature overnight. The crude reaction mixture was analyzed by LC-MS/MS. The column temperature was 45 °C. Normalized collision energies were 20 and 30. The tandem MS data were analyzed and visualized with the Interactive Peptide Spectral Annotator.[4]

*Val-Tyr-**L-threo-MeCys**-Gln-Gly (**1**)*

Peptide **1** was synthesized following the general procedure for Fmoc solid-phase peptide synthesis. The crude material was analyzed by UPLC coupled to HRMS. UPLC (C18 Poroshell column (2.7 µm, 120 Å, 100 × 3.0 mm); gradient: 98% H_2O with 0.1% FA (Solvent A)/2% MeCN with 0.1% FA (Solvent B) for 2 min then a linear gradient of 98:2 to 2:98 Solvent A:Solvent B over 10 min; flow rate = 0.4 mL/min; l = 220 nm) t_R = 5.800 min. HRMS (ESI) m/z: $[M + H]^+$ Calcd for C25H39N6O8S 583.2545; found 583.2576.

*Val-Tyr-**L-erythro-MeCys**-Gln-Gly (**2**)*

Peptide **2** was synthesized following the general procedure for Fmoc solid-phase peptide synthesis. The crude material was analyzed by UPLC coupled to HRMS. UPLC (C18 Poroshell column (2.7 µm, 120 Å, 100 × 3.0 mm); gradient: 98% H_2O with 0.1% FA (Solvent A)/2% MeCN with 0.1% FA (Solvent B) for 2 min then a linear gradient of 98:2 to 2:98 Solvent A:Solvent B over 10 min; flow rate = 0.4 mL/min; l = 220 nm) t_R = 5.859 min. HRMS (ESI) m/z: $[M + H]^+$ Calcd for C25H39N6O8S 583.2545; found 583.2567.

*Val-Tyr-**Z-Dhb**-Gln-Gly (**3**)*

Peptide **3** was synthesized from peptide **2** following the method outlined in 'General procedure for the desulfhydration of thiol-containing peptides with cyanamide'. The crude material was analyzed by UPLC coupled to HRMS. UPLC (C18 Poroshell column (2.7 µm, 120 Å, 100 × 3.0 mm); gradient: 98% H_2O with 0.1% FA (Solvent A)/2% MeCN with 0.1% FA (Solvent B) for 2 min then a linear gradient of 98:2 to 2:98 Solvent A:Solvent B over 10 min; flow rate = 0.4 mL/min; l = 220 nm) t_R = 5.640 min. HRMS (ESI) m/z: $[M + H]^+$ Calcd for C25H37N6O8 549.2668; found 549.2674. Prior to ^{1}H NMR analysis (Figure S1), crude peptide **3** was purified using a Vydac C18 (5 µm, 250 × 4.6 mm) with the following gradient: 98% H_2O with 0.1% TFA (Solvent A)/2% MeCN with 0.1% TFA (Solvent B) for 5 min then a linear gradient of 98:2 to 63:37 Solvent

A:Solvent B over 20 min. The column temperature was 25 °C and the flow rate was 1 mL/min; t_R = 11.7 min. Product-containing fractions were combined and lyophilized.

*Ile-Gly-**Cys**-Thr-Glu-Asn-Leu-Lys (**5**)*

Peptide **5** was synthesized following the general procedure for Fmoc solid-phase peptide synthesis. The crude material was analyzed by UPLC coupled to HRMS. UPLC (Macherey-Nagel C18 EC 125/2 NUCLEODUR 100-5 C18ec column (5 µm, 250 × 4.6 mm); gradient: 98% H_2O with 0.1% FA (Solvent A)/2% MeCN with 0.1% FA (Solvent B) for 2 min then a linear gradient of 98:2 to 2:98 Solvent A:Solvent B over 6 min; flow rate = 0.4 mL/min; l = 220 nm) t_R = 4.934 min. HRMS (ESI) m/z: $[M + H]^+$ Calcd for C36H65N10O13S 877.4448; found 877.4447.

*Ser-Pro-Val-Val-**Cys**-Gly-Asp-Thr-Ser-Pro-Arg (**6**)*

Peptide **6** was synthesized following the general procedure for Fmoc solid-phase peptide synthesis. The crude material was analyzed by UPLC coupled to HRMS. UPLC (Macherey-Nagel C18 EC 125/2 NUCLEODUR 100-5 C18ec column (5 µm, 250 × 4.6 mm); gradient: 98% H_2O with 0.1% FA (Solvent A)/2% MeCN with 0.1% FA (Solvent B) for 2 min then a linear gradient of 98:2 to 2:98 Solvent A:Solvent B over 6 min; flow rate = 0.4 mL/min; l = 220 nm) t_R = 4.897 min. HRMS (ESI) m/z: $[M + H]^+$ Calcd for C45H77N14O17S 1117.5307; found 1117.5410.

*Ile-Gly-**Dha**-Thr-Glu-Asn-Leu-Lys (7)*

Peptide **7** was synthesized from peptide **5** following the method outlined in 'General procedure for the desulfhydration of thiol-containing peptides with cyanamide'. The crude material was analyzed by UPLC coupled to HRMS. UPLC (Macherey-Nagel C18 EC 125/2 NUCLEODUR 100-5 C18ec column (5 µm, 250 × 4.6 mm); gradient: 98% H_2O with 0.1% FA (Solvent A)/2% MeCN with 0.1% FA (Solvent B) for 2 min then a linear gradient of 98:2 to 2:98 Solvent A:Solvent B over 6 min; flow rate = 0.4 mL/min; l = 220 nm) t_R = 4.844 min. HRMS (ESI) m/z: $[M + 2H]^{2+}$ Calcd for C36H63N10O13 422.2322; found 422.2325. Prior to ^{1}H NMR analysis (Figure S5), crude peptide **7** was purified using a Vydac C18 (5 µm, 250 × 4.6 mm) with the following gradient: 98% H_2O with 0.1% TFA (Solvent A)/2% MeCN with 0.1% TFA (Solvent B) for 10 min then a linear gradient of 98:2 to 30:70 Solvent A:Solvent B over 20 min. The column temperature was 25 °C and the flow rate was 1 mL/min; t_R = 20.5 min. Product-containing fractions were combined and lyophilized. Peptide **7** was isolated as a colorless powder (15% yield based on resin loading, 0.202mg).

*Ser-Pro-Val-Val-**Dha**-Gly-Asp-Thr-Ser-Pro-Arg (**8**)*

Peptide **8** was synthesized from peptide **6** following the method outlined in 'General procedure for the desulfhydration of thiol-containing peptides with cyanamide'. The crude material was analyzed by UPLC coupled to HRMS. UPLC (Macherey-Nagel C18 EC 125/2 NUCLEODUR 100-5 C18ec column (5 µm, 250 × 4.6 mm); gradient: 98% H_2O with 0.1% FA (Solvent A)/2% MeCN with 0.1% FA (Solvent B) for 2 min then a linear gradient of 98:2 to 2:98 Solvent A:Solvent B over 6

min; flow rate = 0.4 mL/min; l = 220 nm) t_R = 4.925 min. HRMS (ESI) m/z: $[M + 2H]^{2+}$ Calcd for C45H75N14O17 524.2751; found 524.2778. Prior to ^{1}H NMR analysis (Figure S6), peptide **8** was purified using a Vydac C18 (5 µm, 250 × 4.6 mm) with the following gradient: 98% H_2O with 0.1% TFA (Solvent A)/2% MeCN with 0.1% TFA (Solvent B) for 10 min then a linear gradient of 98:2 to 50:50 Solvent A:Solvent B over 15 min. The column temperature was 25 °C and the flow rate was 1 mL/min; t_R = 20.8 min. Product containing fractions were combined and lyophilized.

*Ala-Tyr-Ala-**Cys**-Asn-Thr-Ser-Thr-Leu (**9**)*

Peptide **9** was purchased from GenScript and was analyzed by UPLC coupled to HRMS. UPLC (Macherey-Nagel C18 EC 125/2 NUCLEODUR 100-5 C18ec column (5 µm, 250 × 4.6 mm); gradient: 98% H_2O with 0.1% FA (Solvent A)/2% MeCN with 0.1% FA (Solvent B) for 2 min then a linear gradient of 98:2 to 2:98 Solvent A:Solvent B over 6 min; flow rate = 0.4 mL/min; l = 220 nm) t_R = 5.378 min. HRMS (ESI) m/z: $[M + H]^+$ Calcd for C39H63N10O15 943.4190; found 943.4248.

*Lys-Leu-Pro-Gln-Leu-**Cys**-Thr-Glu-Leu (**10**)*

Peptide **10** was purchased from GenScript and was analyzed by UPLC coupled to HRMS. UPLC (Macherey-Nagel C18 EC 125/2 NUCLEODUR 100-5 C18ec column (5 µm, 250 × 4.6 mm); gradient: 98% H_2O with 0.1% FA (Solvent A)/2% MeCN with 0.1% FA (Solvent B) for 2 min then a linear gradient of 98:2 to 2:98 Solvent A:Solvent B over 6 min; flow rate = 0.4 mL/min; l = 220 nm) t_R = 4.934 min. HRMS (ESI) m/z: $[M + H]^+$ Calcd for C46H82N11O14S 1044.5758; found 1044.5844.

*Ac-**Cys**-Gly-Ala-Ala-Phe-Gly (**11**)*

Peptide **11** was synthesized following the general procedure for Fmoc SPPS. The crude material was analyzed by UPLC coupled to HRMS. UPLC (Macherey-Nagel C18 EC 125/2 NUCLEODUR 100-5 C18ec column (5 µm, 250 × 4.6 mm); gradient: 98% H_2O with 0.1% FA (Solvent A)/2% MeCN with 0.1% FA (Solvent B) for 2 min then a linear gradient of 98:2 to 2:98 Solvent A:Solvent B over 6 min; flow rate = 0.4 mL/min; l = 220 nm) t_R = 6.566 min. HRMS (ESI) m/z: $[M + H]^+$ Calcd for C24H35N6O8S 567.2199; found 567.2232.

*Ac-**Cys**-**Cys**-Ala-Ala-Phe-Gly (**12**)*

Peptide **12** was synthesized following the general procedure for Fmoc SPPS. The crude material was analyzed by UPLC coupled to HRMS. UPLC (Macherey-Nagel C18 EC 125/2 NUCLEODUR 100-5 C18ec column (5 µm, 250 × 4.6 mm); gradient: 98% H_2O with 0.1% FA (Solvent A)/2% MeCN with 0.1% FA (Solvent B) for 2 min then a linear gradient of 98:2 to 2:98 Solvent A:Solvent B over 6 min; flow rate = 0.4 mL/min; l = 220 nm) t_R = 6.219 min. HRMS (ESI) m/z: $[M + H]^+$ Calcd for C25H37N6O8S2 613.2109; found 613.2127.

*Ac-**Cys(isothiourea)**-Gly-Ala-Ala-Phe-Gly (**13**)*

Peptide **13** was observed during the desulfhydration of peptide **11** following the method outlined in 'General procedure for the desulfhydration of thiol-containing peptides with cyanamide'. The crude material was analyzed by UPLC coupled to HRMS. UPLC (Macherey-Nagel C18 EC 125/2 NUCLEODUR 100-5 C18ec column (5 µm, 250 × 4.6 mm); gradient: 98% H_2O with 0.1% FA (Solvent A)/2% MeCN with 0.1% FA (Solvent B) for 2 min then a linear gradient of 98:2 to 2:98 Solvent A:Solvent B over 6 min; flow rate = 0.4 mL/min; l = 220 nm) t_R = 5.143 min. HRMS (ESI) m/z: $[M + H]^+$ Calcd for C25H37N8O8S 609.2450; found 609.2446.

*Ala-Tyr-Ala-**Dha**-Asn-Thr-Ser-Thr-Leu (**14**)*

Peptide **14** was synthesized from peptide **9** following the method outlined in 'General procedure for the desulfhydration of thiol-containing peptides with cyanamide'. The crude material was analyzed by UPLC coupled to HRMS. UPLC (Macherey-Nagel C18 EC 125/2 NUCLEODUR 100-5 C18ec column (5 µm, 250 × 4.6 mm); gradient: 98% H_2O with 0.1% FA (Solvent A)/2% MeCN with 0.1% FA (Solvent B) for 2 min then a linear gradient of 98:2 to 2:98 Solvent A:Solvent B over 6 min; flow rate = 0.4 mL/min; l = 220 nm) t_R = 5.303 min. HRMS (ESI) m/z: $[M + H]^+$ Calcd for C39H61N10O15 909.4312; found 909.4388.

*Lys-Leu-Pro-Gln-Leu-**Dha**-Thr-Glu-Leu (**15**)*

Peptide **15** was synthesized from peptide **10** following the method outlined in 'General procedure for the desulfhydration of thiol-containing peptides with cyanamide'. The crude material was analyzed by UPLC coupled to HRMS. UPLC (Macherey-Nagel C18 EC 125/2 NUCLEODUR 100-5 C18ec column (5 µm, 250 × 4.6 mm); gradient: 98% H_2O with 0.1% FA (Solvent A)/2% MeCN with 0.1% FA (Solvent B) for 2 min then a linear gradient of 98:2 to 2:98 Solvent A:Solvent B over 6 min; flow rate = 0.4 mL/min; l = 220 nm) t_R = 5.715 min. HRMS (ESI) m/z: $[M + H]^+$ Calcd for C46H80N11O14 1010.5881; found 1010.5955.

*Ac-**Dha**-Gly-Ala-Ala-Phe-Gly (**16**)*

Peptide **16** was synthesized from peptide **11** following the method outlined in 'General procedure for the desulfhydration of thiol-containing peptides with cyanamide'. The crude material was analyzed by UPLC coupled to HRMS. UPLC (Macherey-Nagel C18 EC 125/2 NUCLEODUR 100-5 C18ec column (5 µm, 250 × 4.6 mm); gradient: 98% H_2O with 0.1% FA (Solvent A)/2% MeCN with 0.1% FA (Solvent B) for 2 min then a linear gradient of 98:2 to 2:98 Solvent A:Solvent B over 6 min; flow rate = 0.4 mL/min; l = 220 nm) t_R = 6.335 min. HRMS (ESI) m/z: $[M + H]^+$ Calcd for C24H33N6O8 533.2354; found 533.2317.

*Ac-**Dha**-**Dha**-Ala-Ala-Phe-Gly (**17**)*

Peptide **17** was synthesized from peptide **12** following the method outlined in 'General procedure for the desulfhydration of thiol-containing peptides with cyanamide'. The crude material was analyzed by UPLC coupled to HRMS. UPLC (Macherey-Nagel C18 EC 125/2 NUCLEODUR 100-5 C18ec column (5 µm, 250 × 4.6 mm); gradient: 98% H_2O with 0.1% FA (Solvent A)/2%

MeCN with 0.1% FA (Solvent B) for 2 min then a linear gradient of 98:2 to 2:98 Solvent A:Solvent B over 6 min; flow rate = 0.4 mL/min; l = 220 nm) t_R = 6.581 min. HRMS (ESI) m/z: $[M + H]^+$ Calcd for C25H33N6O8 545.2354; found 545.2319.

*Ala-Tyr-Ala-**Cys(isothiourea)**-Asn-Thr-Ser-Thr-Leu (**18**)*

Peptide **18** was observed during the desulfhydration of peptide **9** following the method outlined in 'General procedure for the desulfhydration of thiol-containing peptides with cyanamide'. The crude material was analyzed by UPLC coupled to HRMS. UPLC (Macherey-Nagel C18 EC 125/2 NUCLEODUR 100-5 C18ec column (5 µm, 250 × 4.6 mm); gradient: 98% H_2O with 0.1% FA (Solvent A)/2% MeCN with 0.1% FA (Solvent B) for 2 min then a linear gradient of 98:2 to 2:98 Solvent A:Solvent B over 6 min; flow rate = 0.4 mL/min; l = 220 nm) t_R = 4.835 min. HRMS (ESI) m/z: $[M + 2H]^{2+}$ Calcd for C40H65N12O15S 493.2240; found 493.2272.

*Ala-Tyr-Ala-**Cys(isothiourea)**-Asn-Thr-Ser-Thr-Leu (**19**)*

Peptide **19** was observed during the desulfhydration of peptide **10** following the method outlined in 'General procedure for the desulfhydration of thiol-containing peptides with cyanamide'. The crude material was analyzed by UPLC coupled to HRMS. UPLC (Macherey-Nagel C18 EC 125/2 NUCLEODUR 100-5 C18ec column (5 µm, 250 × 4.6 mm); gradient: 98% H_2O with 0.1% FA (Solvent A)/2% MeCN with 0.1% FA (Solvent B) for 2 min then a linear gradient of 98:2 to 2:98 Solvent A:Solvent B over 6 min; flow rate = 0.4 mL/min; l = 220 nm) t_R = 5.341 min. HRMS (ESI) m/z: $[M + 2H]^{2+}$ Calcd for C47H84N13O14S 543.8024; found 543.8072.

*Leu-Gly-**Cys**-Gly-Glu-**Cys(Acm)**-Leu-Lys (**20**)*

Peptide **20** was synthesized following the general procedure for Fmoc SPPS. The peptide was purified by reversed-phase HPLC (Luna C18 column (10 µm, 250 × 10 mm); gradient: 1:0 H_2O with 0.1 % TFA (Solvent A):Acetonitrile with 0.1% TFA (Solvent B) for 5 min, then a linear gradient from 100:0 to 30:70 (Solvent A:Solvent B) over 35 min, then a linear gradient from 30:70 to 5:95 (Solvent A:Solvent B) over 5 min; flow rate = 4 mL/min; l = 220 nm) t_R = 20.8 min. Product-containing fractions were combined and lyophilized. Purified peptide **20** was analyzed by UPLC (Macherey-Nagel C18 EC 125/2 NUCLEODUR 100-5 C18ec column (5 µm, 250 × 4.6 mm); gradient: 98% H_2O with 0.1% FA (Solvent A)/2% MeCN with 0.1% FA (Solvent B) for 2 min then a linear gradient of 98:2 to 2:98 Solvent A:Solvent B over 10 min; flow rate = 0.4 mL/min; l = 205 nm) t_R = 5.121 min. HRMS (ESI) m/z: $[M + H]^+$ Calcd for C36H65N10O12S2 893.4219; found 893.4245.

*Leu-Gly-**Dha**-Gly-Glu-**Cys(Acm)**-Leu-Lys (**21**)*

Peptide **21** was synthesized from peptide **20** following the method outlined in 'General procedure for the desulfhydration of thiol-containing peptides with cyanamide'. The crude material was analyzed by UPLC coupled to HRMS. UPLC (Macherey-Nagel C18 EC 125/2 NUCLEODUR 100-5 C18ec column (5 µm, 250 × 4.6 mm); gradient: 98% H_2O with 0.1% FA (Solvent A)/2%

MeCN with 0.1% FA (Solvent B) for 2 min then a linear gradient of 98:2 to 2:98 Solvent A:Solvent B over 10 min; flow rate = 0.4 mL/min; l = 205 nm) t_R = 5.050 min. HRMS (ESI) m/z: $[M + H]^+$ Calcd for C36H63N10O12S 859.4342; found 859.4352.

*Phe-Glu-**Penicillamine**-Leu-Lys (22)*

Peptide **22** was synthesized following the general procedure for Fmoc SPPS. The peptide was purified by reversed-phase HPLC (Luna C18 column (10 µm, 250 × 10 mm); gradient: 1:0 H_2O with 0.1 % TFA (Solvent A):Acetonitrile with 0.1% TFA (Solvent B) for 5 min, then a linear gradient from 100:0 to 30:70 (Solvent A:Solvent B) over 35 min, then a linear gradient from 30:70 to 5:95 (Solvent A:Solvent B) over 5 min; flow rate = 4 mL/min; l = 220 nm) t_R = 19.1 min. Product-containing fractions were combined and lyophilized. Purified peptide **22** was analyzed by UPLC (Macherey-Nagel C18 EC 125/2 NUCLEODUR 100-5 C18ec column (5 µm, 250 × 4.6 mm); gradient: 98% H_2O with 0.1% FA (Solvent A)/2% MeCN with 0.1% FA (Solvent B) for 2 min then a linear gradient of 98:2 to 2:98 Solvent A:Solvent B over 10 min; flow rate = 0.4 mL/min; l = 205 nm) t_R = 5.357 min. HRMS (ESI) m/z: $[M + H]^+$ Calcd for C31H51N6O8S 667.3484; found 667.3524.

*Phe-Glu-**dehydrovaline**-Leu-Lys (23)*

Trace quantities of peptide **23** were synthesized from peptide **22** following the method outlined in 'General procedure for the desulfhydration of thiol-containing peptides with cyanamide'. The crude material was analyzed by UPLC coupled to HRMS. UPLC (Macherey-Nagel C18 EC 125/2 NUCLEODUR 100-5 C18ec column (5 µm, 250 × 4.6 mm); gradient: 98% H_2O with 0.1% FA (Solvent A)/2% MeCN with 0.1% FA (Solvent B) for 2 min then a linear gradient of 98:2 to 2:98 Solvent A:Solvent B over 10 min; flow rate = 0.4 mL/min; l = 205 nm) t_R = 5.442 min. HRMS (ESI) m/z: $[M + H]^+$ Calcd for C31H49N6O$_8$ 633.3606; found 633.3606.

Conversion of modified His$_6$-Bib1A2-S64C to

Modified His$_6$-Bib1A2-S64C was prepared by recombinant co-expression of His$_6$-Bib1A2-S64C and Bib1M in *E. coli* as described previously.[5] Modified His$_6$-Bib1A2-S64C (65.4mg, 8.84 µmol, 1.00 equiv.) was dissolved in MQ H_2O (1.30 mL) and this solution was diluted in MQ H_2O (7.54 mL) containing cyanamide (37.1 mg, 884 µmol, 100 equiv.) and sodium phosphate (53.0 mg, 442 µmol, 50 equiv.). The pH of the solution was adjusted to 8 using concentrated NaOH. The reaction mixture was incubated at room temperature for 18 h. Reaction completion was evaluated through MALDI-ToF analysis of a sample of the crude reaction mixture desalted using a ZipTip® containing C18 resin (Sigma). MS (ESI) m/z: $[M + H]^+$ Calcd for C327H507N96O94S3 7378.70; found 7378.90.

Conversion of modified Bib1 A2 Ser64Dha to Bib1βDha

The reaction mixture was transferred to an Amicon® centrifugal filter (Sigma) with a 3 kDa molecular weight cutoff. The filtering unit was centrifuged at 3000 × g for 20 min and the retentate

was diluted to its original volume with phosphate buffered saline (PBS). Centrifugation followed by dilution with PBS was repeated two more times. Protease CylA was prepared as described previously[6] and added to a final concentration of 40 nM. After 6 h at room temperature, the reaction was acidified with TFA to a pH of 4 and Bib1β_{Dha} was purified by RP-HPLC following a previously reported method.[5] The peptide was characterized by LC-MS/MS. See Figures S18 and S19.

Table S1. The effect of amidine transfer reagents on samples of 1 and 2

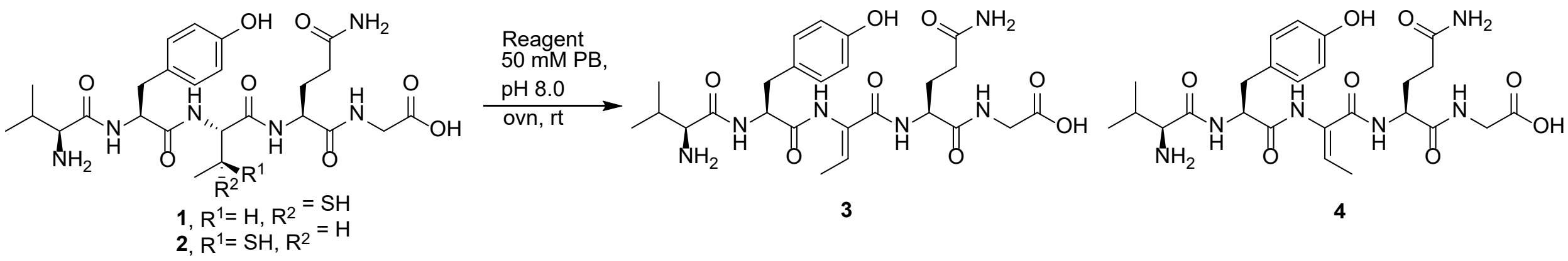

Entry	Starting material[a]	Reagent[b]	Outcome[c]
1	**2**	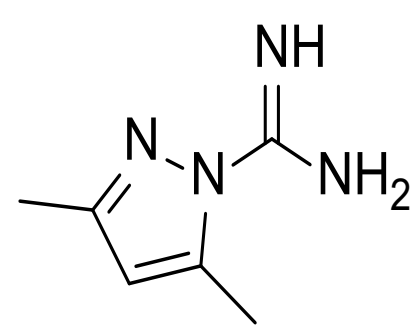	Trace isothiourea adducts detected
2	**2**	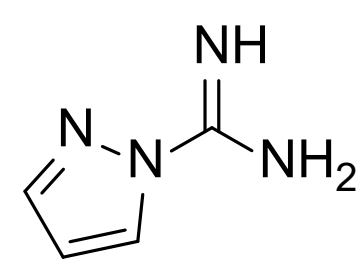	Disulfide of starting material detected
3	**2**	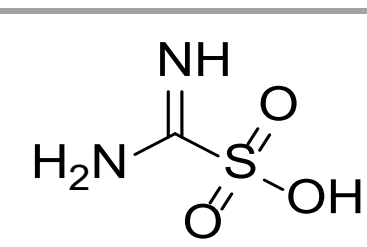	Trace **3** and/or **4** formed
4	**1**	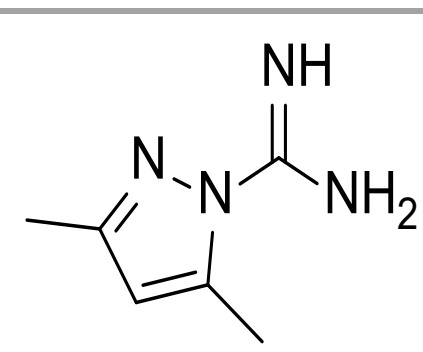	Trace **3** and/or **4** formed
5	**1**	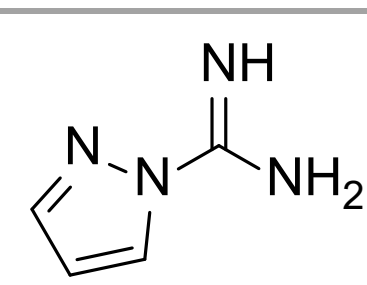	Trace **3** and/or **4** formed
6	**1**	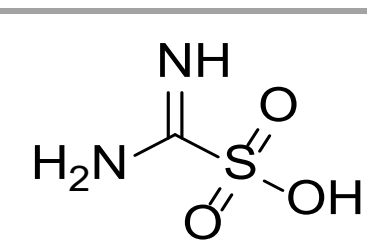	Trace **3** and/or **4** formed

[a] Concentration of starting material was 1 mM for all reactions. [b] Concentration of reagent was 100 mM. [c]Determined by LCMS analysis of the crude reaction mixture after incubating overnight at room temperature.

Table S2. The effect of pH and buffer system on the conversion of 1 to 3

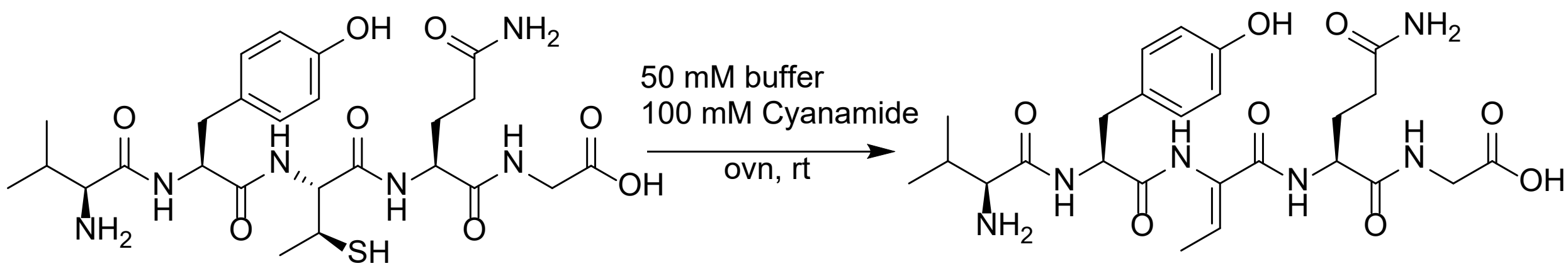

Entry	pH[a]	Buffer	Peak area of **1**[b]	Peak area of **3**[b]
1	5	Acetate	85	0
2	8	HEPES	0	84
3	8	Tricine	0	103
4	8	Tris	0	120
5	8	Phosphate	0	70

[a] pH was evaluated after adding cyanamide. [b] Peak area was determined by integrating the UV-vis trace recorded at 205 nm.

Table S3. Summary of LCMS characterization data collected on peptides 1-23

Compound number	Sequence	Retention time	Ion	Observed mass	Expected mass	Difference (ppm)
**1**	Val-Tyr-**L-*threo*-MeCys**-Gln-Gly	5.800	M+H	583.2576	583.2545	5.32
**2**	Val-Tyr-**L-*erythro*-MeCys**-Gln-Gly	5.859	M+H	583.2567	583.2545	3.77
**3**	Val-Tyr-***Z*-Dhb**-Gln-Gly	5.640	M+H	549.2674	549.2668	1.09
**4**	Val-Tyr-***E*-Dhb**-Gln-Gly	N/A	N/A	N/A	549.2668	N/A
**5**	Ile-Gly-**Cys**-Thr-Glu-Asn-Leu-Lys	4.934	M+H	877.4447	877.4448	0.11
**6**	Ser-Pro-Val-Val-**Cys**-Gly-Asp-Thr-Ser-Pro-Arg	4.897	M+H	1117.5410	1117.5307	9.22
**7**	Ile-Gly-**Dha**-Thr-Glu-Asn-Leu-Lys	4.844	M+2H	422.2325	422.2322	0.71
**8**	Ser-Pro-Val-Val-**Dha**-Gly-Asp-Thr-Ser-Pro-Arg	4.925	M+2H	542.2778	542.2751	4.98
**9**	Ala-Tyr-Ala-**Cys**-Asn-Thr-Ser-Thr-Leu	5.378	M+H	943.4248	943.4190	6.15
**10**	Lys-Leu-Pro-Gln-Leu-**Cys**-Thr-Glu-Leu	5.715	M+H	1044.5844	1044.5758	8.23
**11**	Ac-**Cys**-Gly-Ala-Ala-Phe-Gly	6.566	M+H	567.2199	567.2232	5.82
**12**	Ac-**Cys-Cys**-Ala-Ala-Phe-Gly	6.219	M+H	613.2127	613.2109	2.94
**13**	Ac-**Cys(isothiourea)**-Gly-Ala-Ala-Phe-Gly	5.143	M+H	609.2446	609.2450	0.66
**14**	Ala-Tyr-Ala-**Dha**-Asn-Thr-Ser-Thr-Leu	5.303	M+H	909.4388	909.4312	8.36
**15**	Lys-Leu-Pro-Gln-Leu-**Dha**-Thr-Glu-Leu	5.715	M+H	1010.5955	1010.5881	7.32
**16**	Ac-**Dha**-Gly-Ala-Ala-Phe-Gly	6.335	M+H	533.2317	533.2354	6.94
**17**	Ac-**Dha-Dha**-Ala-Ala-Phe-Gly	6.581	M+H	545.2319	545.2354	6.42
**18**	Ala-Tyr-Ala-**Cys(isothiourea)**-	4.835	M+2H	493.2272	493.2240	6.49

	Asn-Thr-Ser-Thr-Leu					
**19**	Lys-Leu-Pro-Gln-Leu-**Cys(isothiourea)**-Thr-Glu-Leu	5.341	M+2H	543.8072	543.8024	8.83
**20**	Leu-Gly-**Cys**-Gly-Glu-**Cys(Acm)**-Leu-Lys	5.121	M+H	893.4245	893.4219	2.91
**21**	Leu-Gly-**Dha**-Gly-Glu-**Cys(Acm)**-Leu-Lys	5.050	M+H	859.4342	859.4352	1.16
**22**	Phe-Glu-**Penicilliamine**-Leu-Lys	5.357	M+H	667.3524	667.3484	5.99
**23**	Phe-Glu-**dehydrovaline**-Leu-Lys	5.442	M+H	633.3606	633.3606	0.00

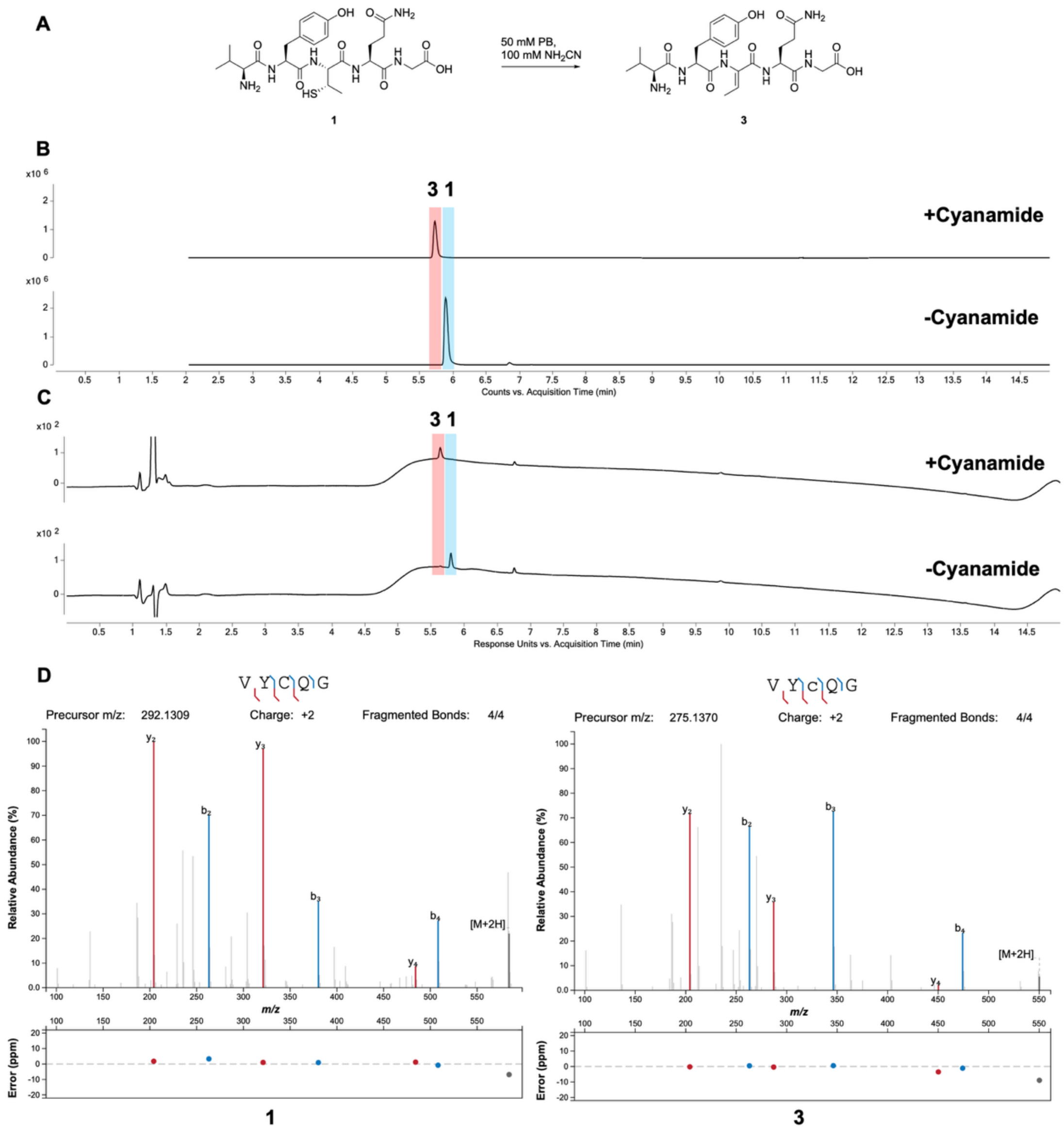

Figure S1. Conversion of **1** to **3**. A) Reaction scheme for the conversion of **1** to **3**. B) and C) Solutions of **1** analyzed by LCMS before and after treatment with cyanamide. B) Extracted ion-chromatograms extracting the following m/z: 583.2545 (**1**) and 549.2668 (**3**). C) LC traces collected using UV-vis detection. The wavelength was set to 220 nm. For B) and C) the samples were not purified by HPLC prior to analysis. D) High resolution MS-MS analysis of substrate and product. MeCys residues are indicated by upper-case C. Dhb residues are indicated by lower-case c. The Interactive Peptide Annotator tool was used to make D).[4]

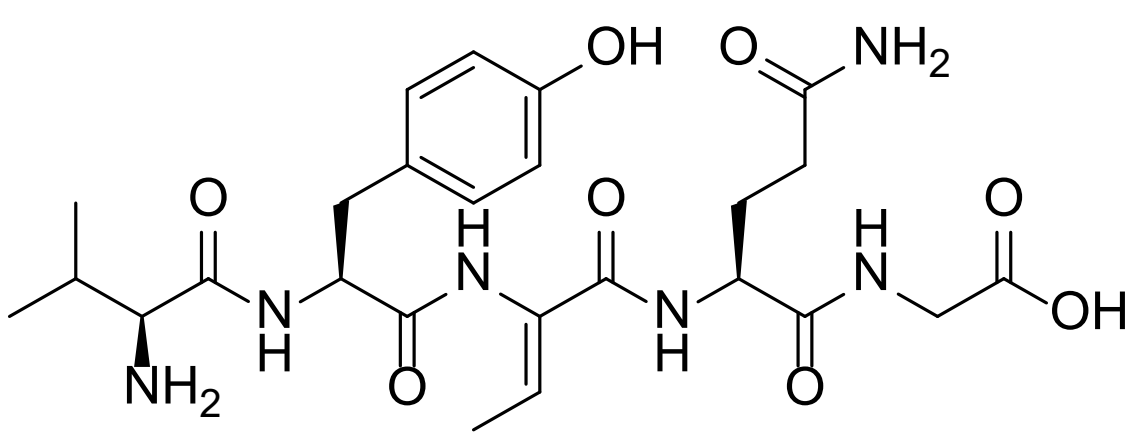

3

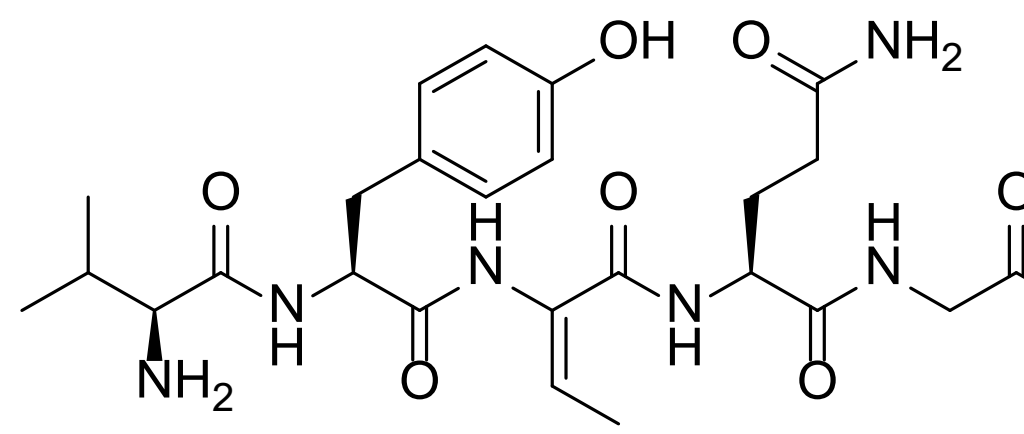

4

^{1}H NMR spectrum of a mixture of **3** and **4**

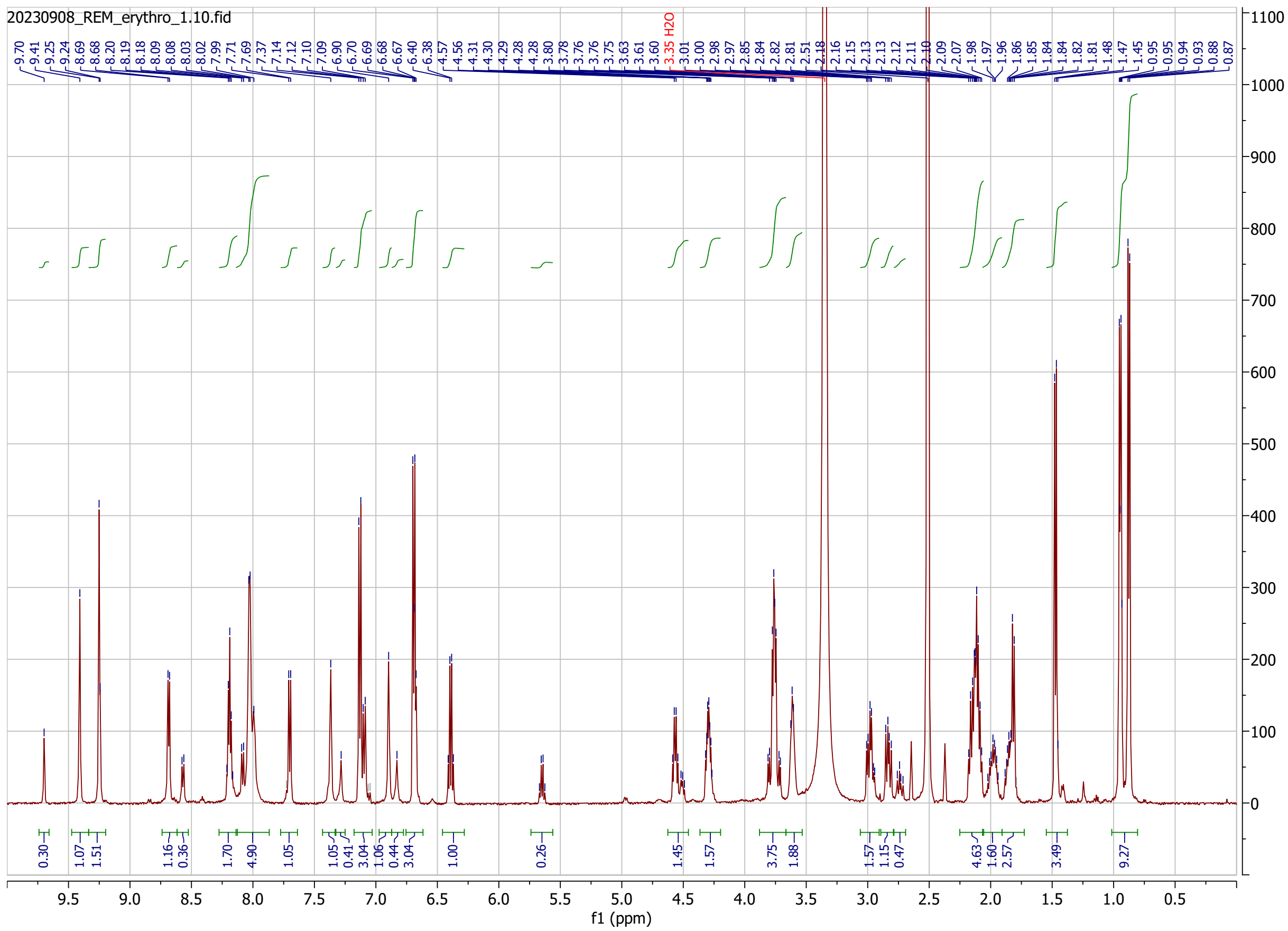

Figure S2. ^{1}H NMR (500 MHz, DMSO-d$_6$) of a mixture of **3** and **4** synthesized by bis-alkylation Method 1, which is different than the conditions listed in Table 1, and purified by HPLC. The two resonances corresponding to the β-H of *E*- and *Z*-Dhb possessed chemical shifts of 5.65 ppm and 6.39 ppm, respectively.

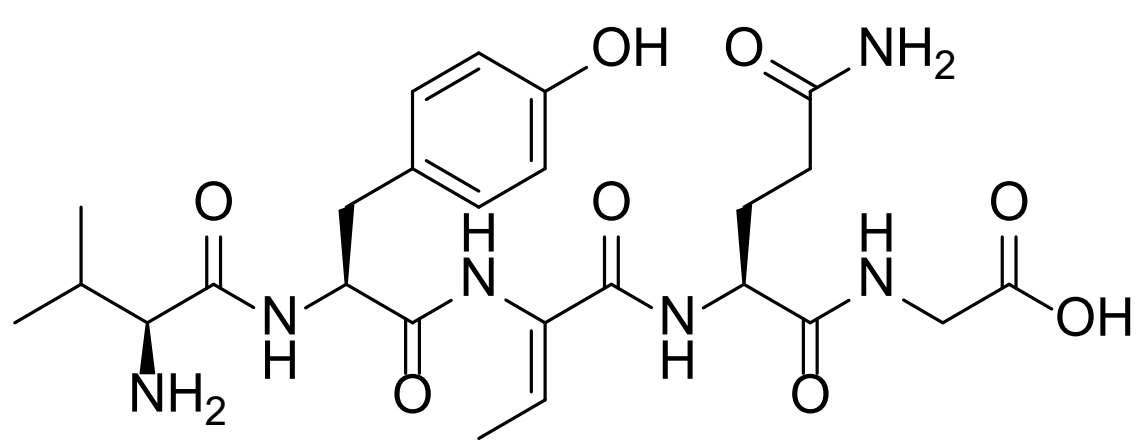

3

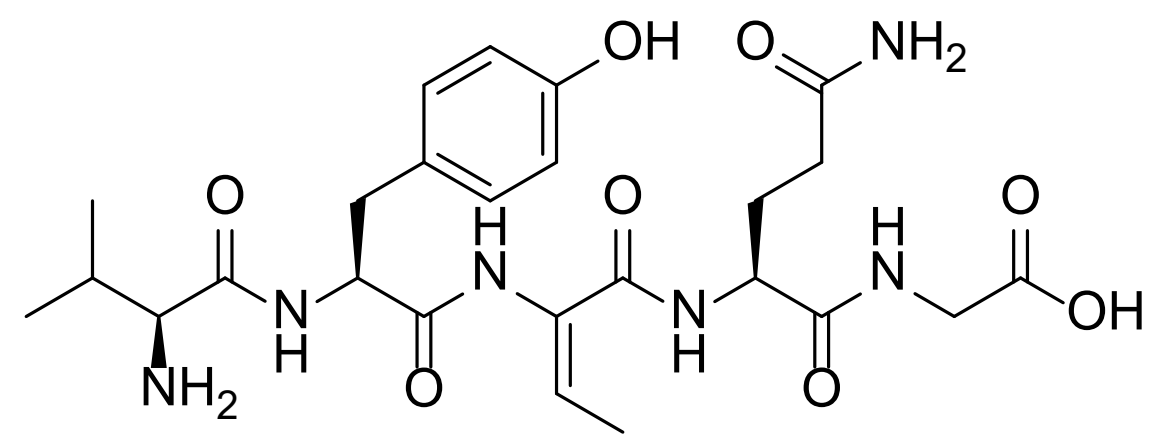

4

Two-dimensional NOESY spectrum of a mixture of **3** and **4**

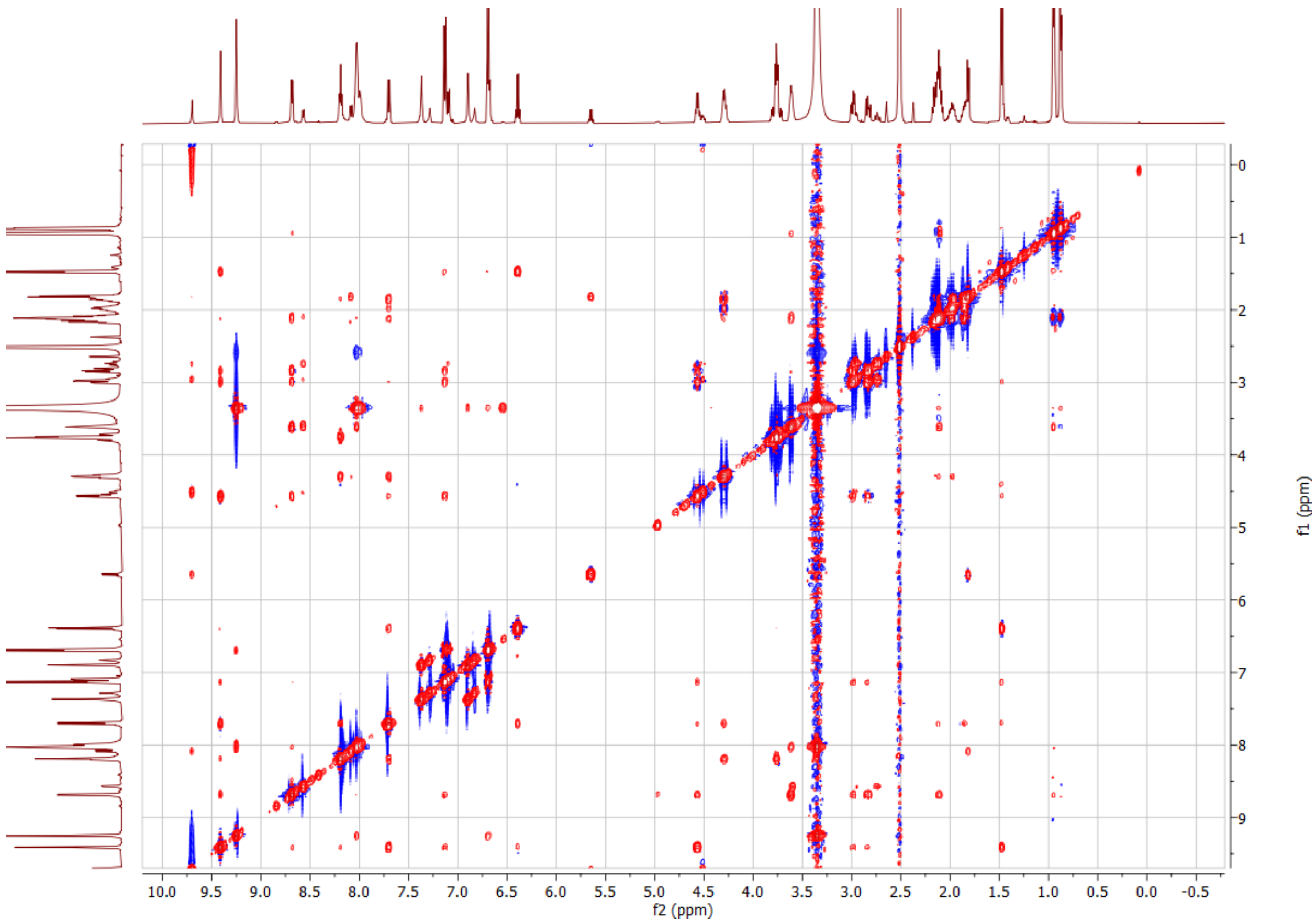

Figure S3. Two-dimensional NOESY (500 MHz, DMSO-d$_6$) spectrum of a mixture of **3** and **4** synthesized by bis-alkylation Method 1 and purified by HPLC. A cross peak at 9.70 ppm and 5.65 ppm indicates that these resonances are part of an *E*-Dhb system.

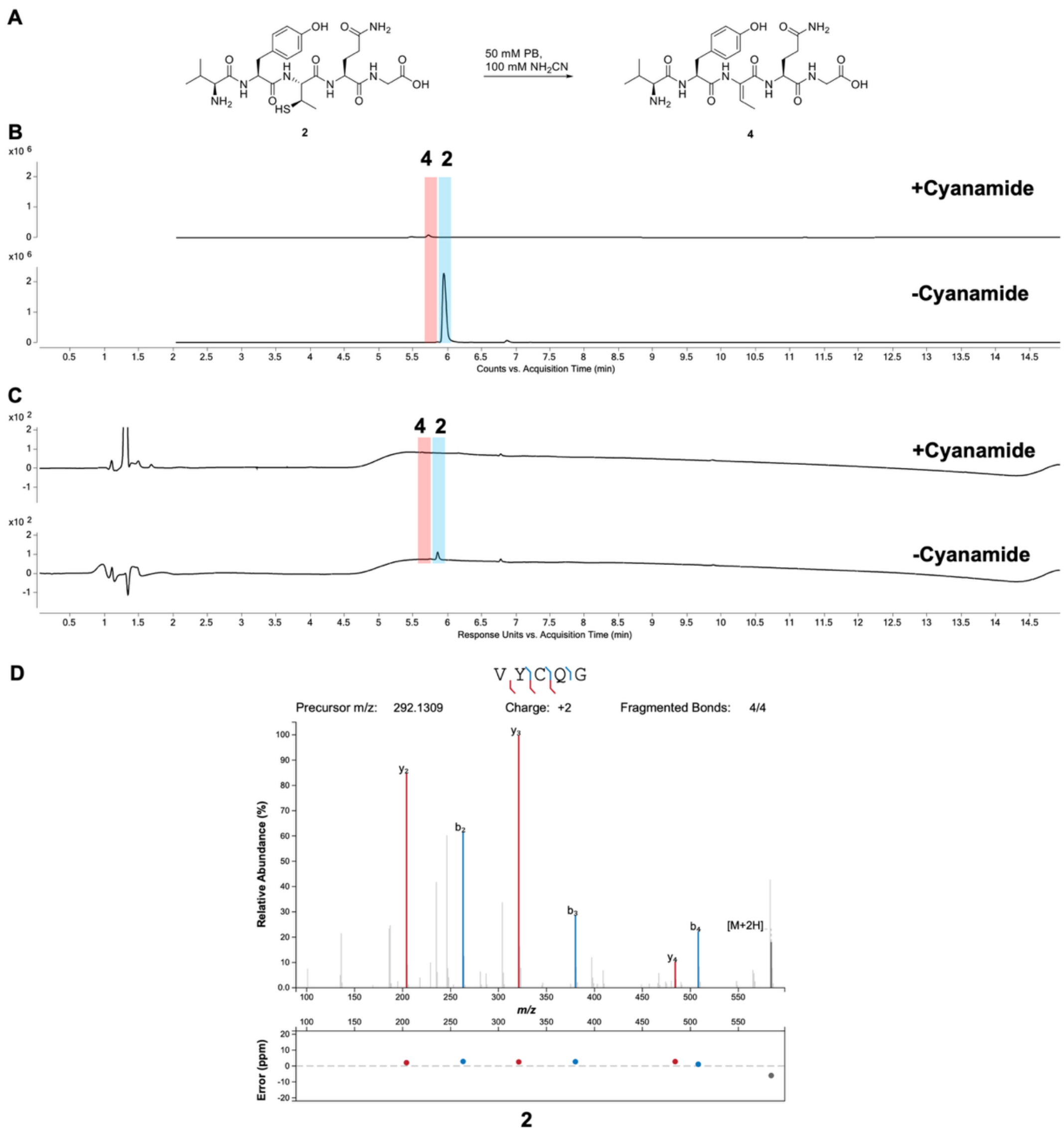

Figure S4. Attempted conversion of **2** to **4**. A) Reaction scheme for the conversion of **2** to **4**. B) and C) Solutions of **2** analyzed by LCMS before and after treatment with cyanamide. B) Extracted ion-chromatograms extracting the following m/z: 583.2545 (**2**) and 549.2668 (**3**/**4**). C) LC traces collected using UV-vis detection. The wavelength was set to 220 nm. For B) and C) the samples were not purified by HPLC prior to analysis. D) High resolution MS-MS analysis of substrate. The Interactive Peptide Annotator tool was used to make D).[4] MeCys residues are indicated by upper-case C.

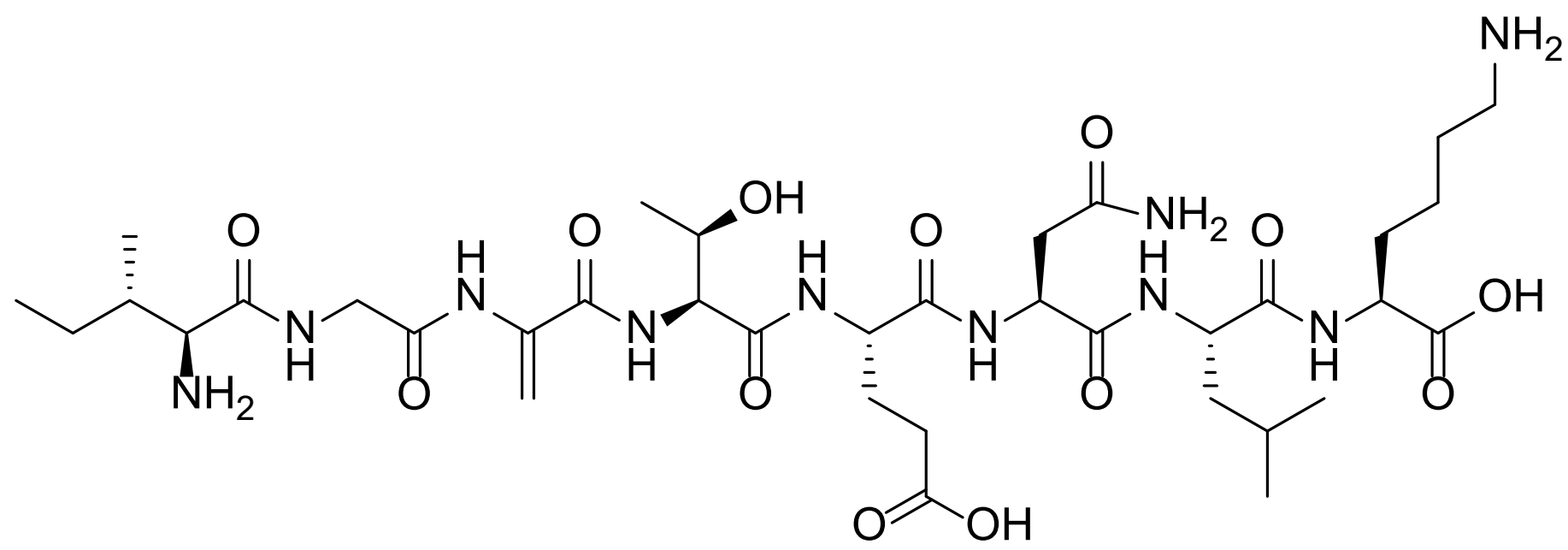

^{1}H NMR spectrum of **7**

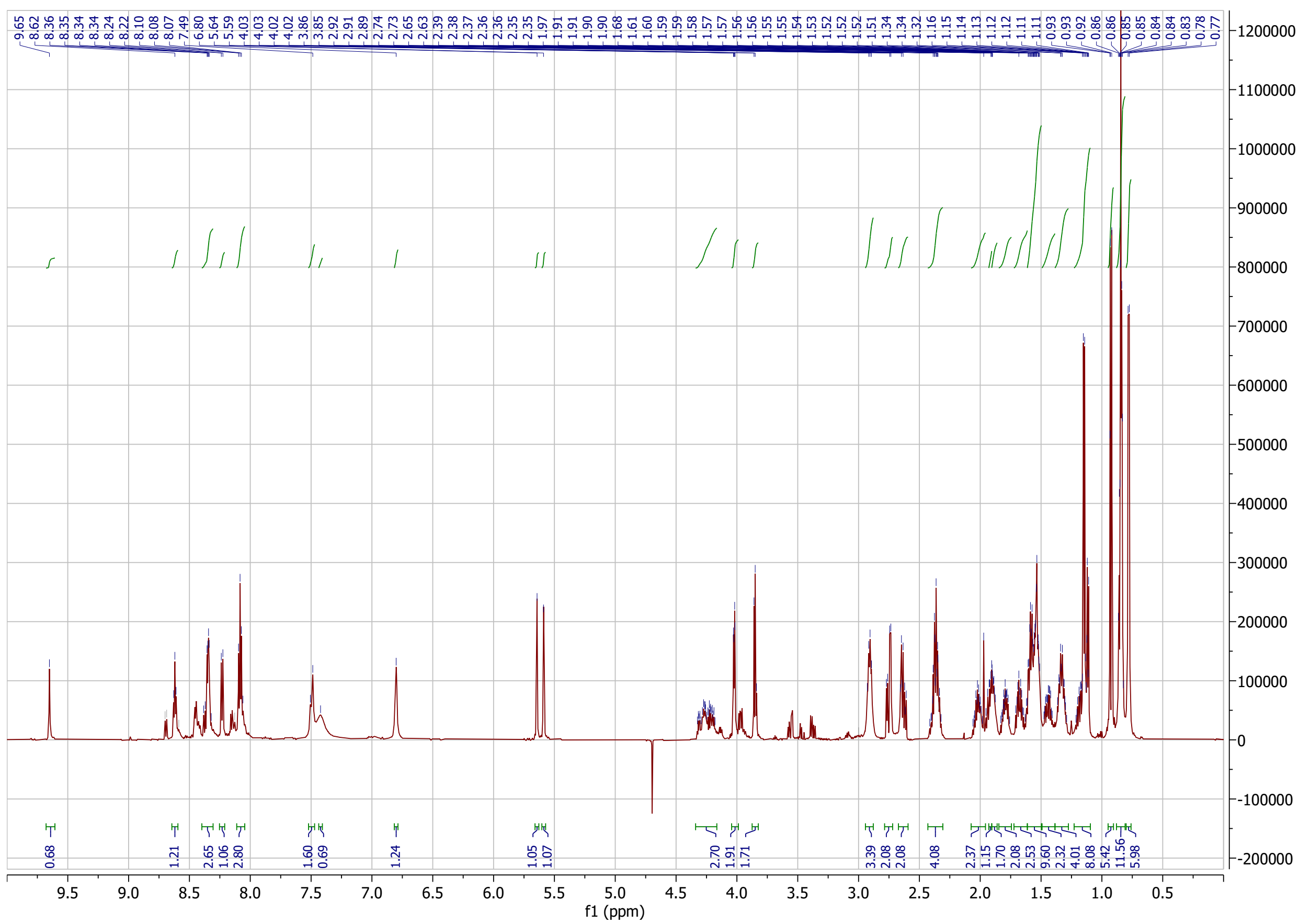

Figure S5. ^{1}H NMR (600 MHz, 10% D_2O/90% H_2O) of Ile-Gly-**<u>Dha</u>**-Thr-Glu-Asn-Leu-Lys (**7**) prepared via treatment of Ile-Gly-**<u>Cys</u>**-Thr-Glu-Asn-Leu-Lys (**5**) with cyanamide/phosphate buffer followed by HPLC purification. The resonances at 9.65 and 5.64 ppm are the α-NH and β-CH_2 of Dha, respectively.

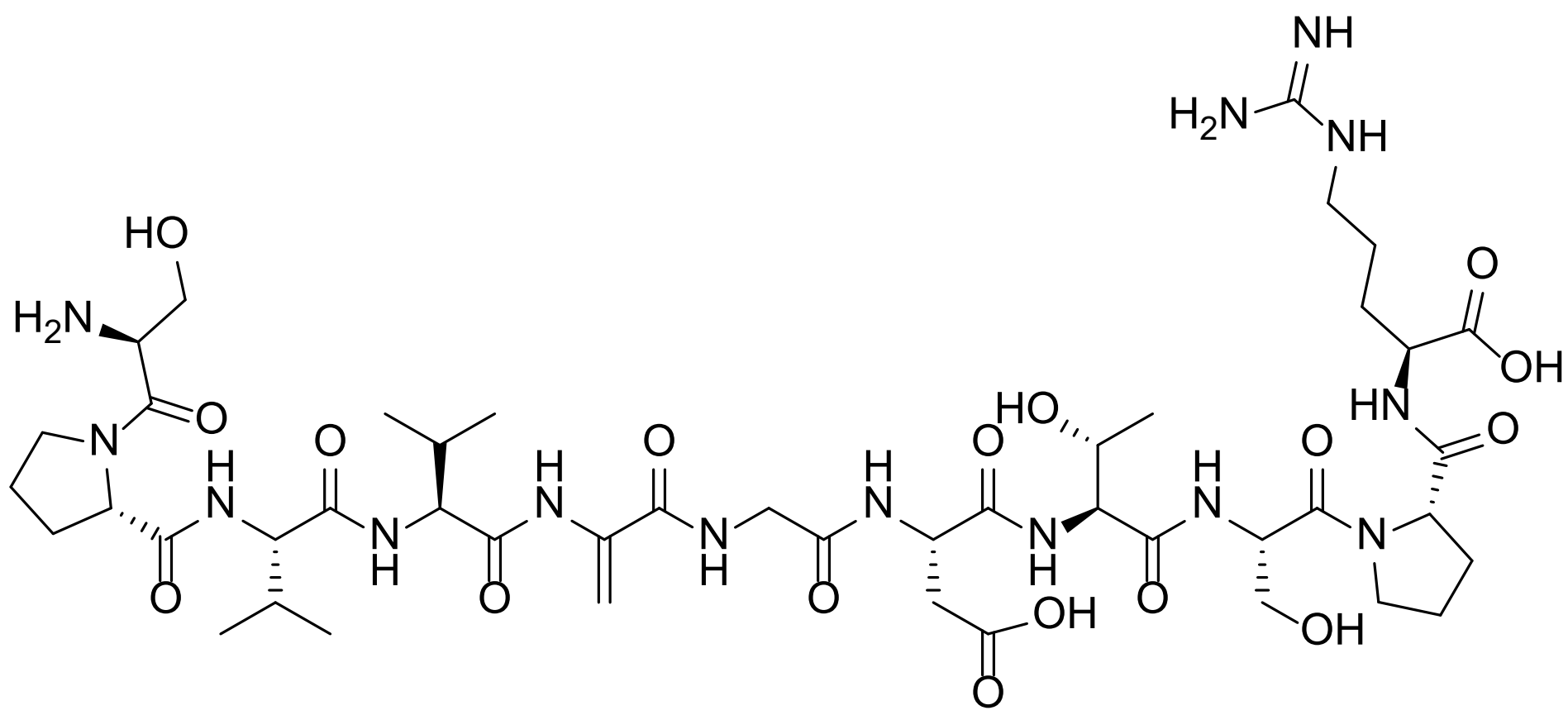

^{1}H NMR spectrum of **8**

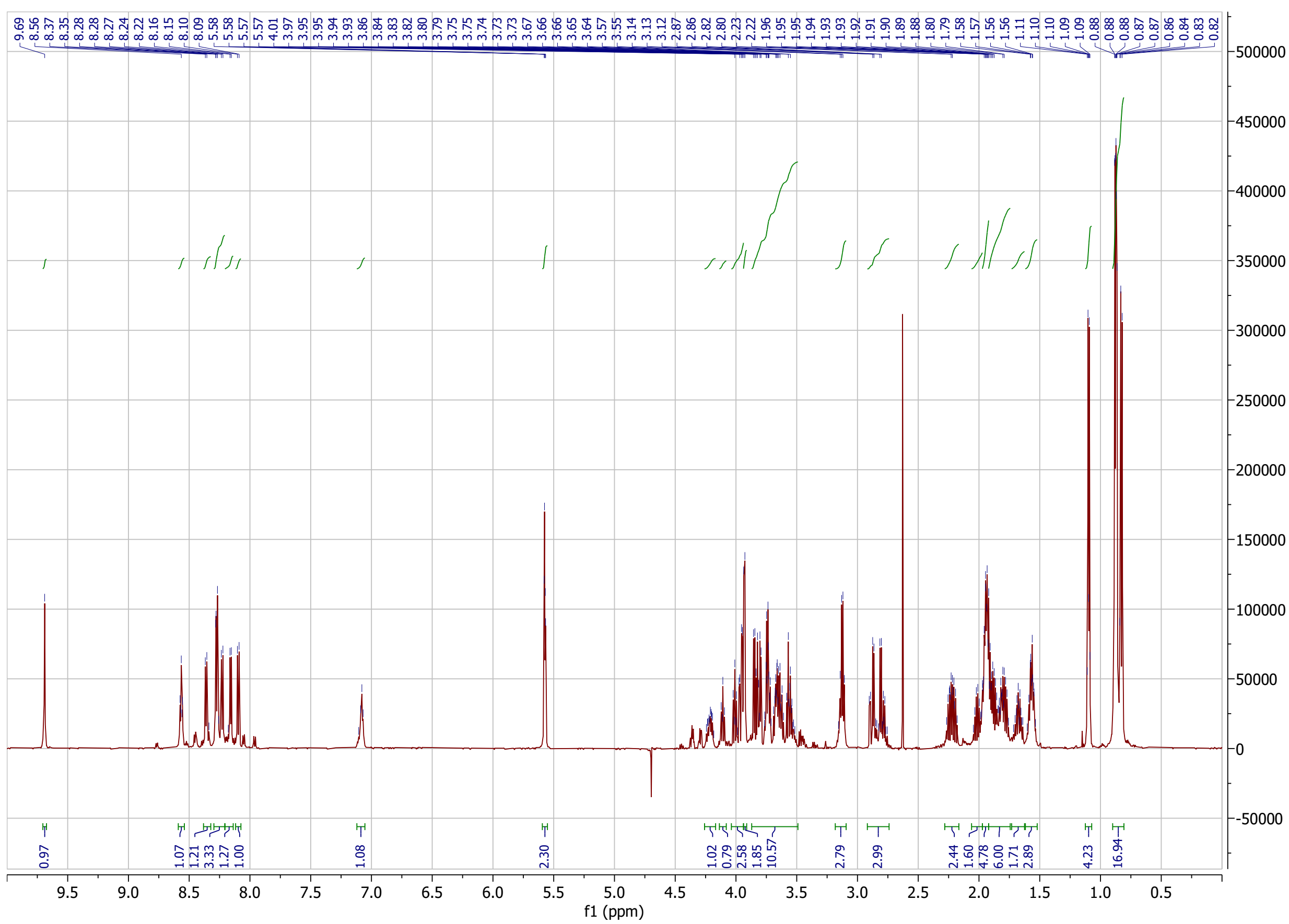

Figure S6. ^{1}H NMR (600 MHz, 10% D_2O/90% H_2O) of Ser-Pro-Val-Val-**Dha**-Gly-Asp-Thr-Ser-Pro-Arg (**8**) prepared via treatment of Ser-Pro-Val-Val-**Cys**-Gly-Asp-Thr-Ser-Pro-Arg (**6**) with cyanamide/phosphate buffer followed by HPLC purification. The resonances at 9.69 and 5.58 ppm are the α-NH and β-CH_2 of Dha, respectively.

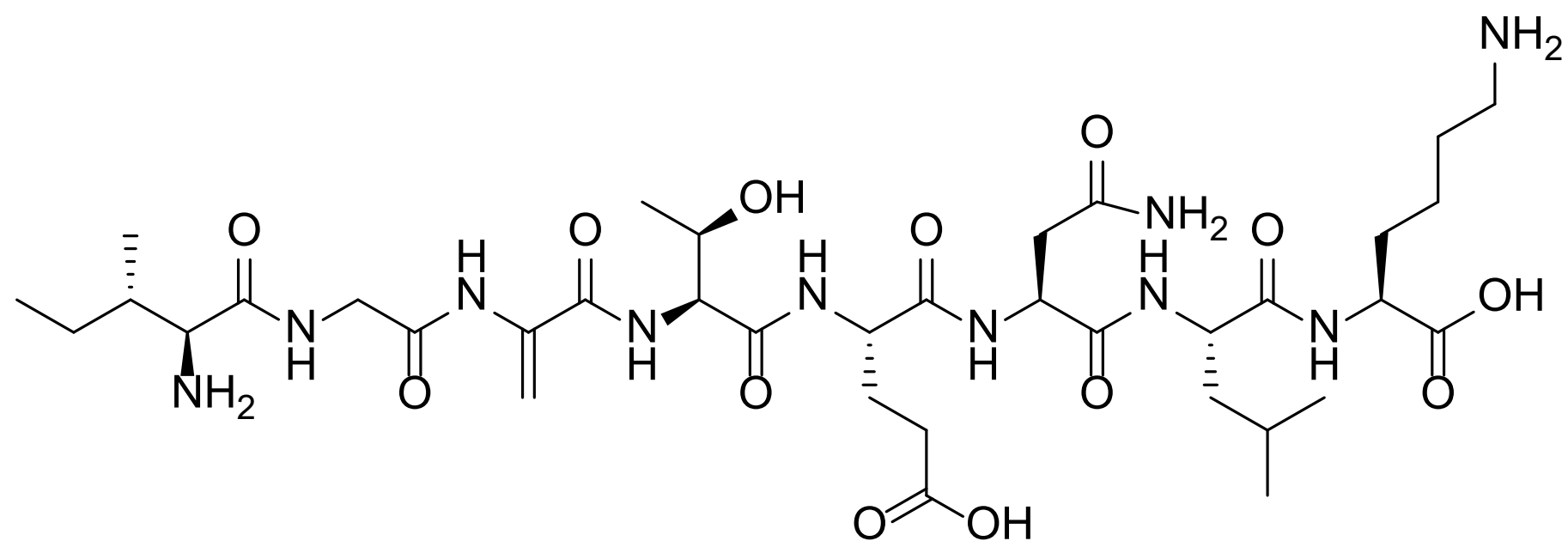

^{1}H NMR spectrum of **7**

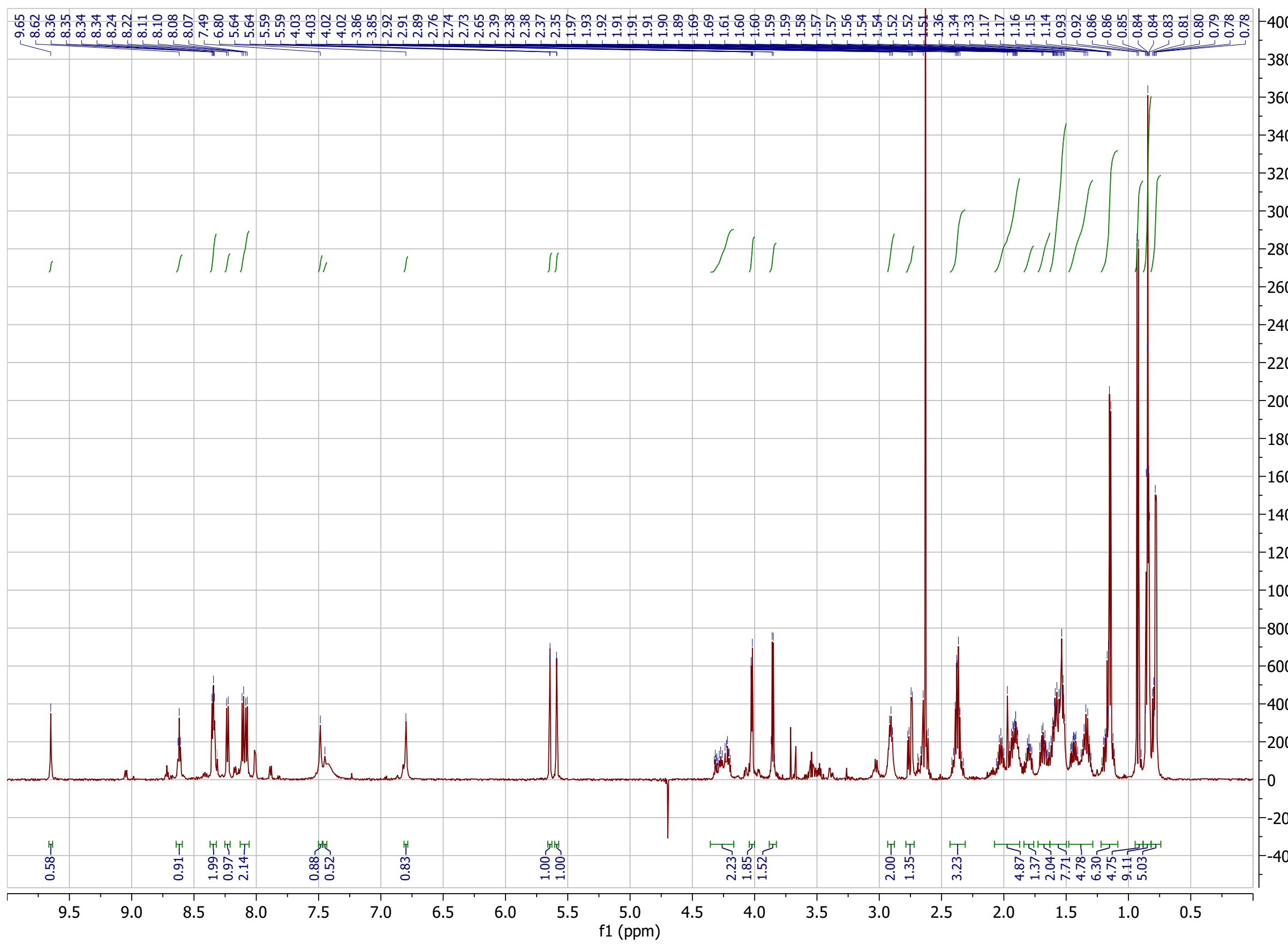

Figure S7. ^{1}H NMR (600 MHz, 10% D_2O/90% H_2O) of Ile-Gly-**<u>Dha</u>**-Thr-Glu-Asn-Leu-Lys (**7**) prepared via treatment of Ile-Gly-**<u>Cys</u>**-Thr-Glu-Asn-Leu-Lys (**5**) with MDBP/K_2CO_3 in 1:1 DMSO:H_2O followed by HPLC purification. The resonances at 9.65 and 5.64 ppm are the α-NH and β-CH_2 of Dha, respectively.

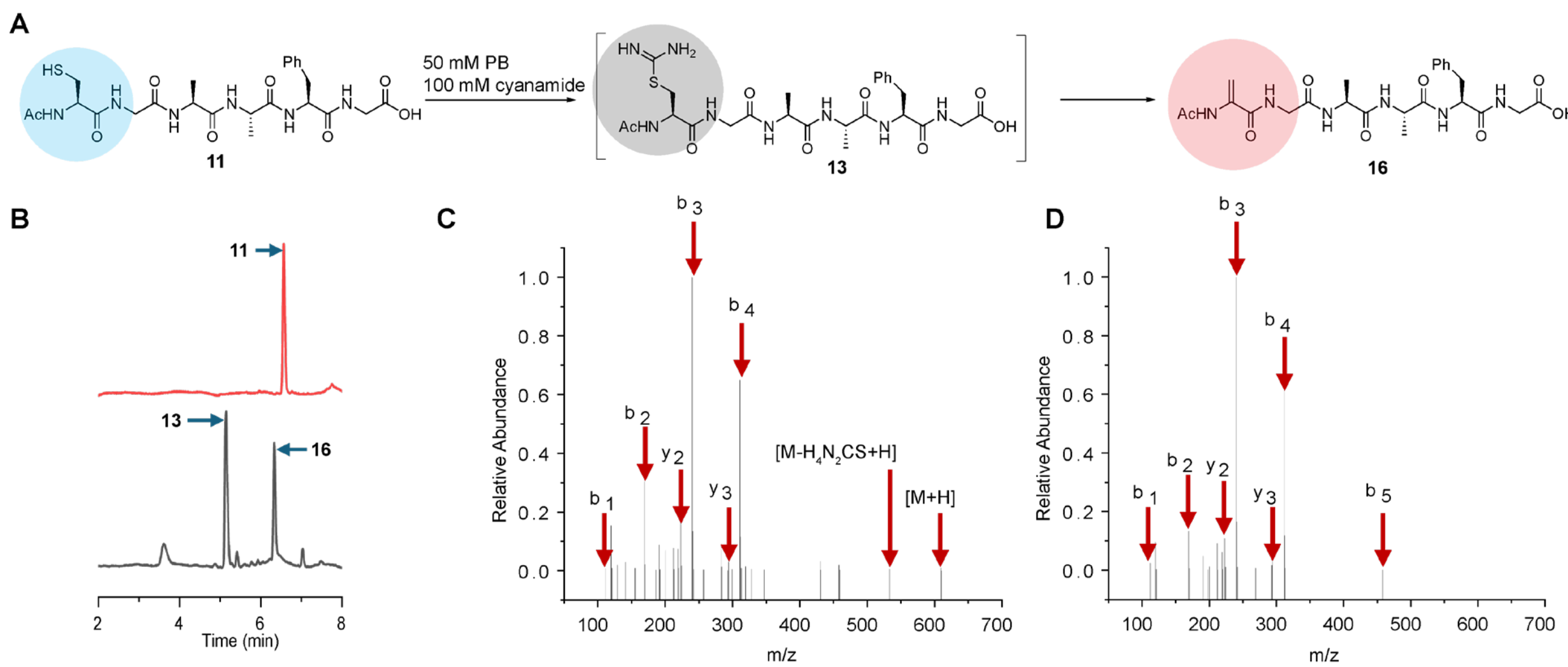

Figure S8. Proposed mechanism of elimination. A) Conversion of **11** to **16** proceeds via isothiourea **13**. B) UV-vis traces collected on a sample of starting material (**11**) and the reaction mixture after incubation overnight at room temperature. Traces were baseline corrected in Origin Pro. C) Tandem MS-MS analysis of **13** showing facile loss of thiourea and further fragmentation of the Dha-containing peptide **16**. D) Tandem MS analysis of Dha-containing peptide **16**.

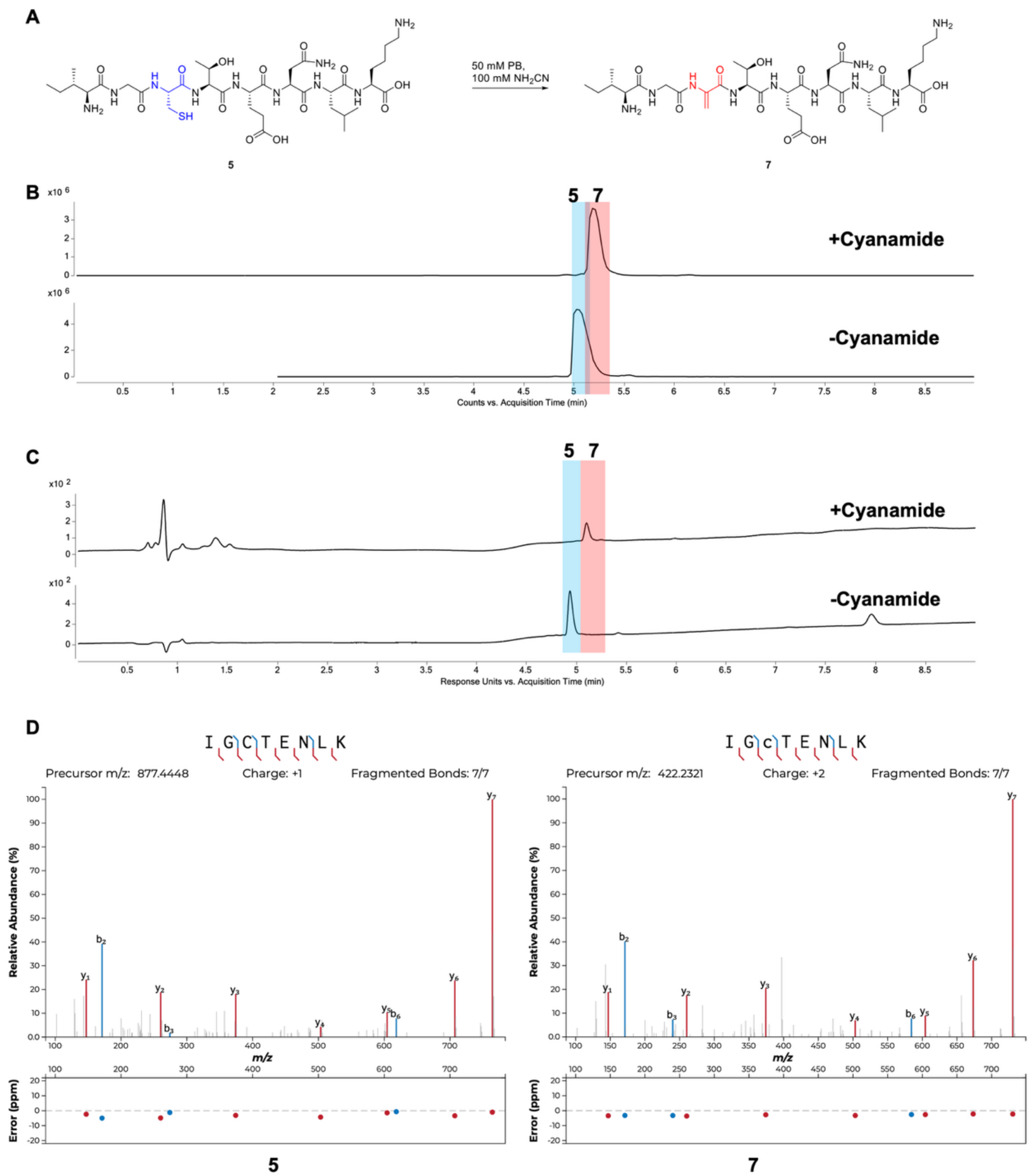

Figure S9. Conversion of **5** to **7**. A) Reaction scheme for the conversion of **5** to **7**. B) and C) Solutions of **5** analyzed by LCMS before and after treatment with cyanamide. B) Extracted ion-chromatograms extracting the following m/z: 877.4448 (**5**) and 422.2322 (**7**). C) LC traces collected using UV-vis detection. The wavelength was set to 220 nm. For B) and C) the samples were not purified by HPLC prior to analysis. D) High resolution MS-MS analysis of substrate and product. Dha residues are indicated by lower-case c. Unmodified Cys residues are indicated by an upper-case C. The Interactive Peptide Annotator tool was used to make panel D).[4]

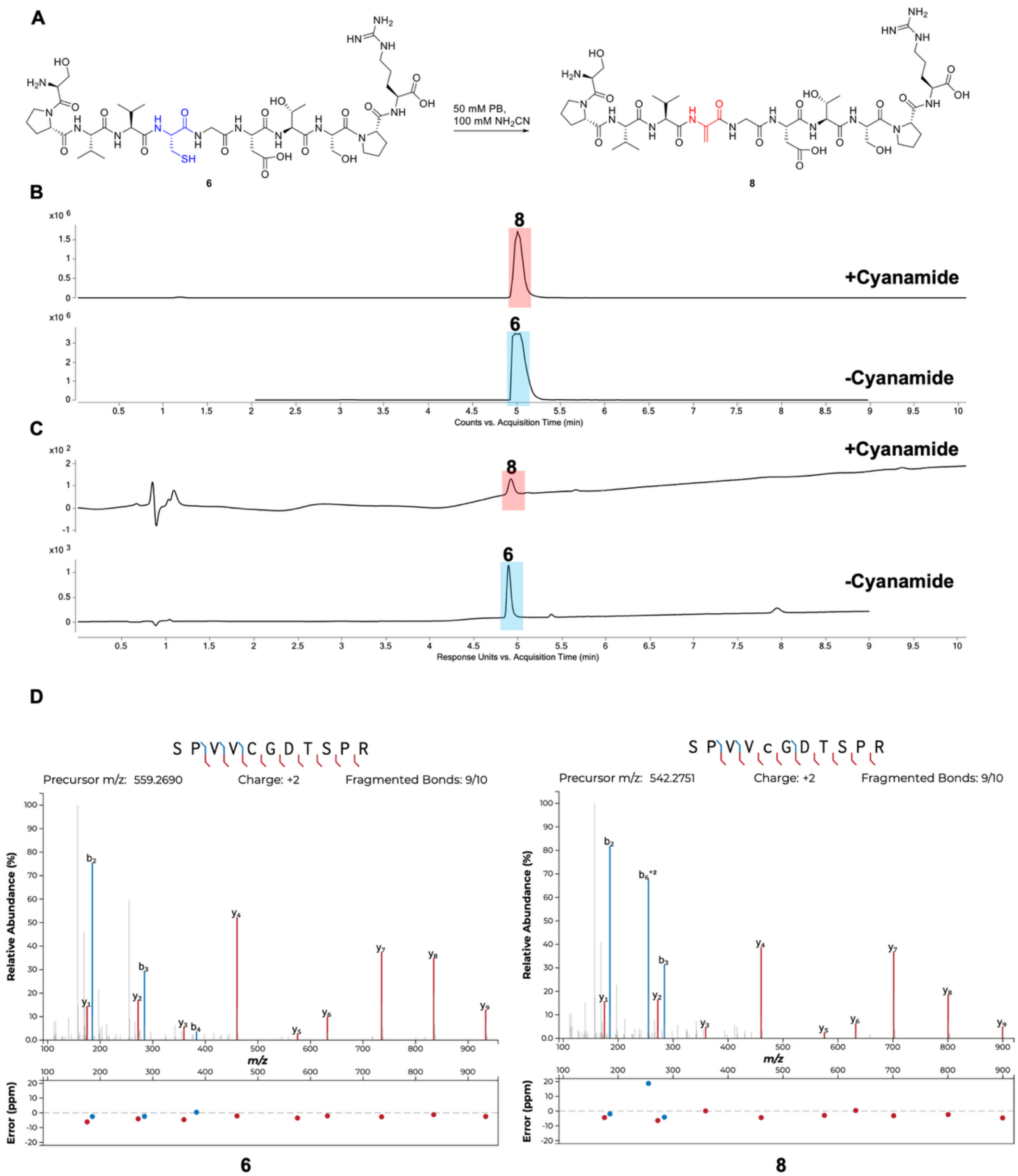

Figure S10. Conversion of **6** to **8**. A) Reaction scheme for the conversion of **6** to **8**. B) and C) Solutions of **6** analyzed by LCMS before and after treatment with cyanamide. B) Extracted ion-chromatograms were generated through extracting the following masses: 1117.5307 (**6**) and 542.2751 (**8**). C) LC traces collected using UV-vis detection. The wavelength was set to 220 nm. For B) and C) the samples were not purified by HPLC prior to analysis. D) High resolution MS-MS analysis of substrate and product. Dha residues are indicated by lower-case c. Unmodified Cys residues are indicated by an upper-case C. The Interactive Peptide Annotator tool was used to make panel D).[4]

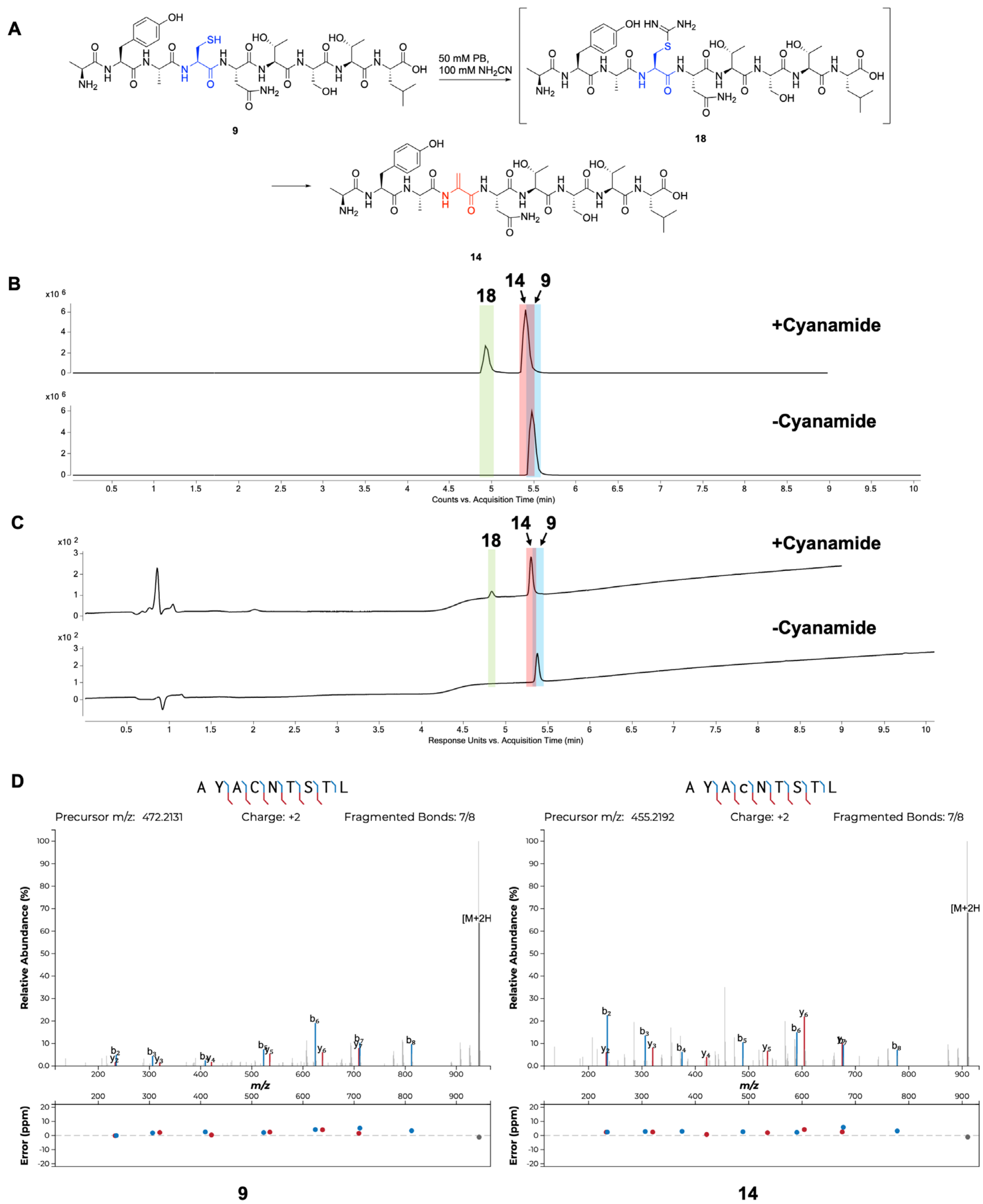

Figure S11. Conversion of **9** to **14** via **18**. A) Reaction scheme showing the conversion of **9** to **14**. B) and C) Solutions of **9** analyzed by LCMS before and after treatment with cyanamide. B) Extracted ion-chromatograms extracting the following masses: 943.4190 (**9**), 493.2240 (**18**), and 909.4312 (**14**). C) LC traces collected using UV-vis detection. The wavelength was set to 220 nm. For B) and C) the samples were not purified by HPLC prior to analysis. D) High resolution MS-MS analysis of substrate and product. Dha residues are indicated by lower-case c. Unmodified Cys residues are indicated by an upper-case C. High resolution MS-MS analysis of intermediates is shown in Figure S17. The Interactive Peptide Annotator tool was used to make panel D).[4]

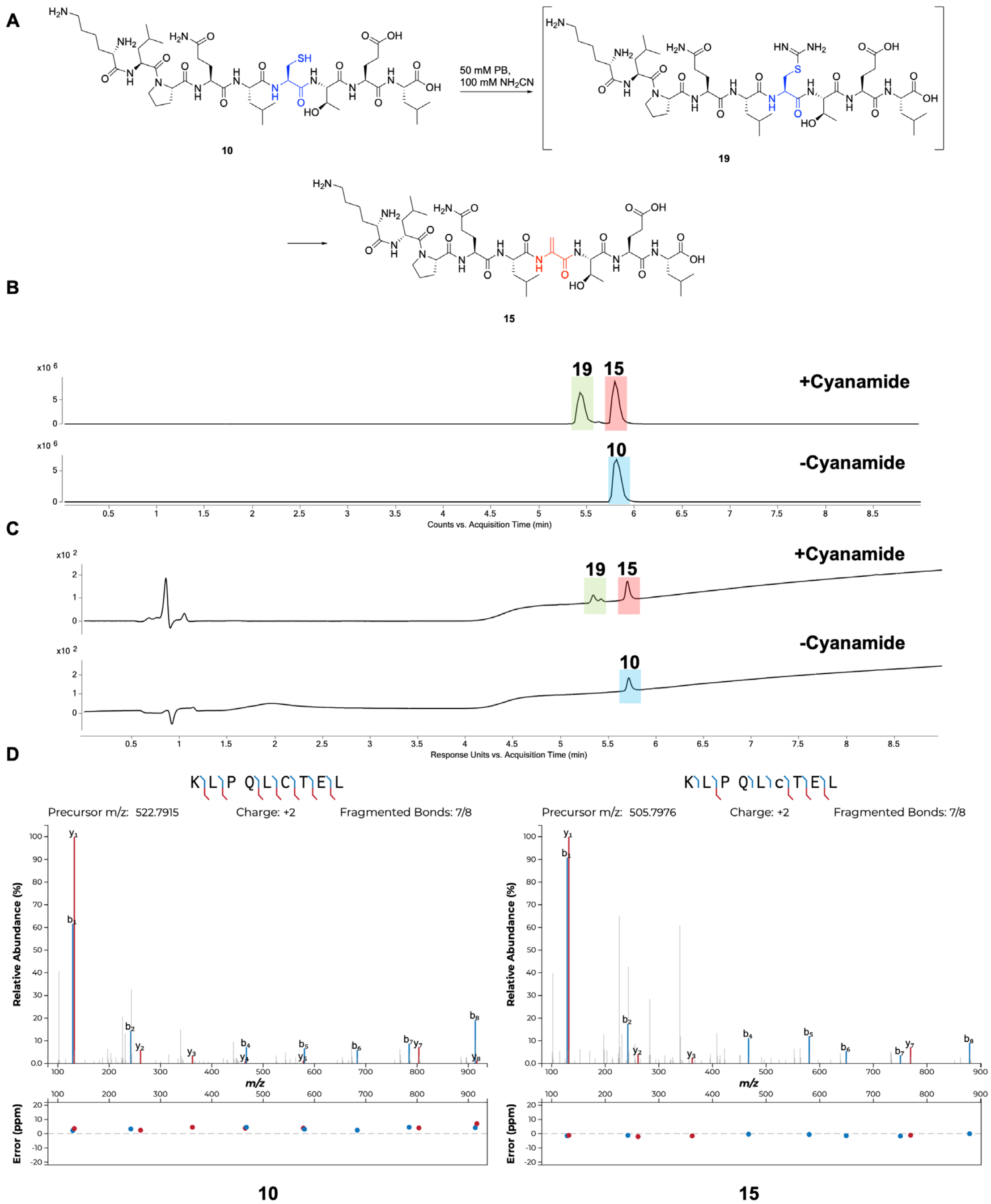

Figure S12. Conversion of **10** to **15** via **19**. A) Reaction scheme for the conversion of **10** to **15**. B) and C) Solutions of **10** analyzed by LCMS before and after treatment with cyanamide. B) Extracted ion-chromatograms extracting the following masses: 1044.5758 (**10**), 543.8024 (**19**), and 1010.5881 (**15**). C) LC traces collected using UV-vis detection. The wavelength was set to 220 nm. For B) and C) the samples were not purified by HPLC prior to analysis. D) High resolution MS-MS analysis of substrate and product. Dha residues are indicated by lower-case c. Unmodified Cys residues are indicated by an upper-case C. High resolution MS-MS analysis of intermediates is shown in Figure S17. The Interactive Peptide Annotator tool was used to make panel D).[4]

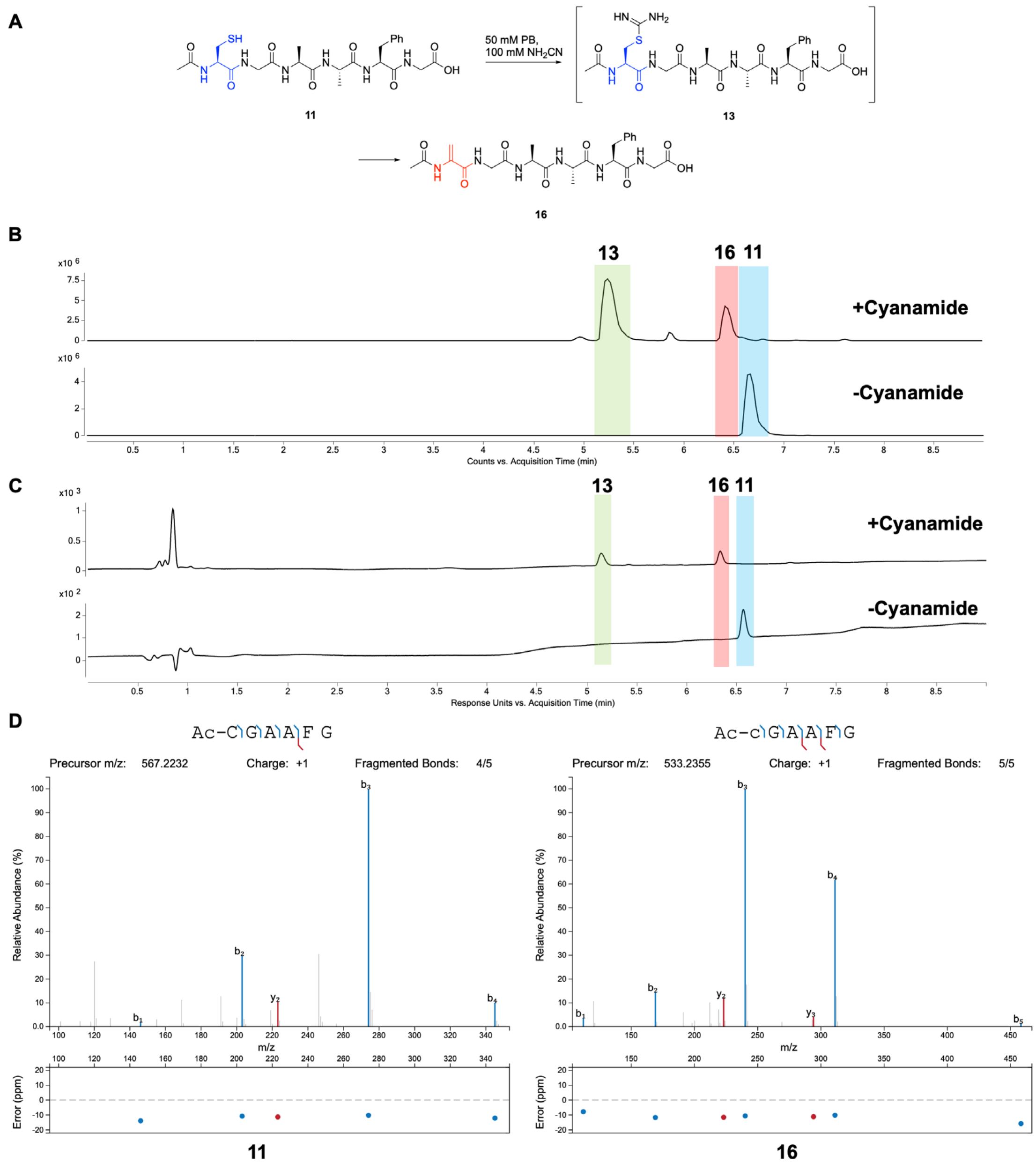

Figure S13. Conversion of **11** to **16** via **13**. A) Reaction scheme for the conversion of **11** to **16**. B) and C) Solutions of **11** analyzed by LCMS before and after treatment with cyanamide. B) Extracted ion-chromatograms generated through extracting the following masses: 567.2232 (**11**), 609.2450 (**13**), and 533.2354 (**16**). C) LC traces collected using UV-vis detection. The wavelength was set to 220 nm. For B) and C) the samples were not purified by HPLC prior to analysis. D) High resolution MS-MS analysis of substrate and product. Dha residues are indicated by lower-case c. Unmodified Cys residues are indicated by an upper-case C. High resolution MS-MS analysis of intermediates is shown in Figure S17. The Interactive Peptide Annotator tool was used to make panel D).[4]

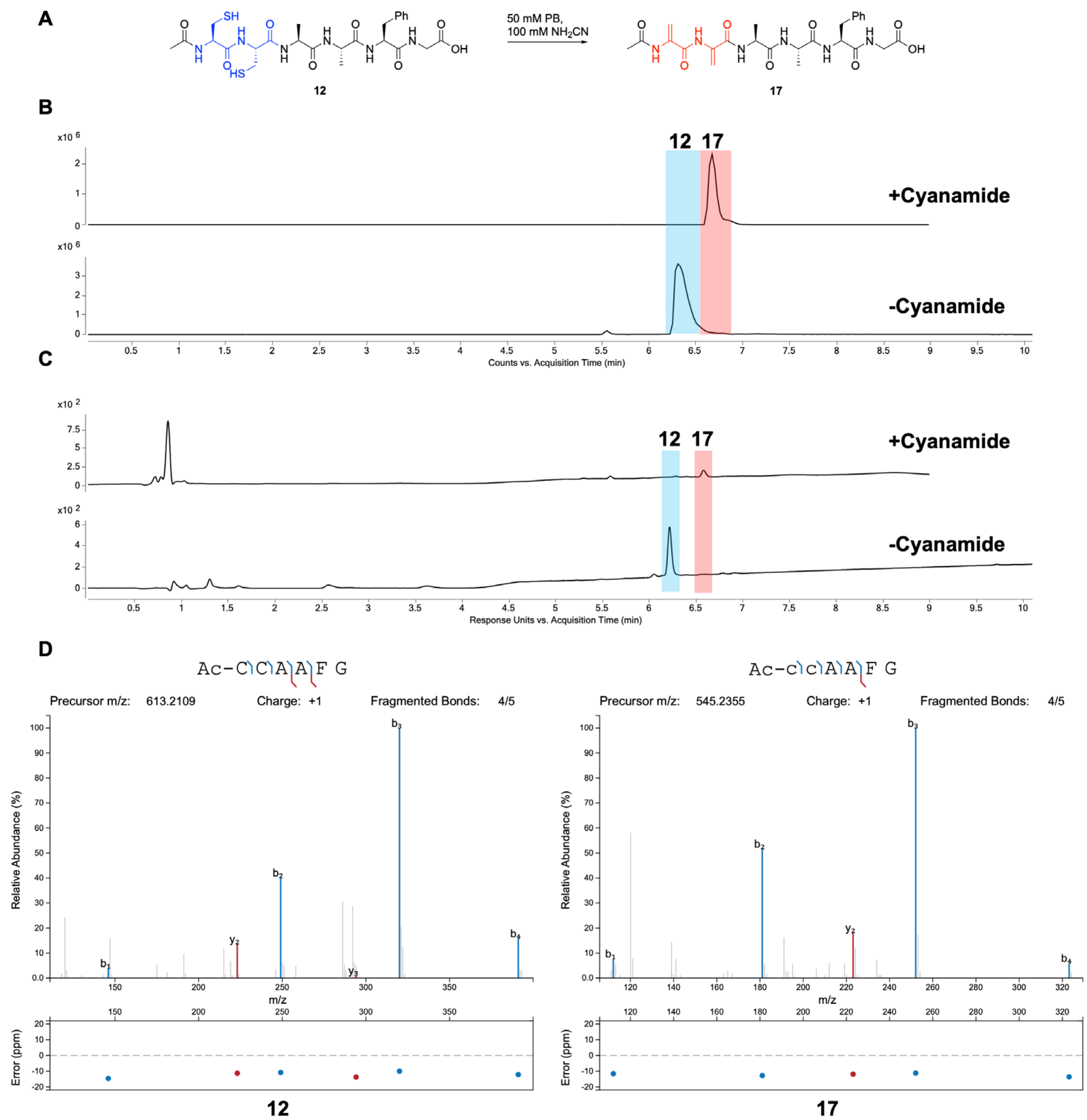

Figure S14. Conversion of **12** to **17**. A) Reaction scheme for the conversion of **12** to **17**. B) and C) Solutions of **12** analyzed by LCMS before and after treatment with cyanamide. B) Extracted ion-chromatograms extracting the following masses: 613.2109 (**12**) and 545.2354 (**17**). C) LC traces collected using UV-vis detection. The wavelength was set to 220 nm. For B) and C) the samples were not purified by HPLC prior to analysis. D) High resolution MS-MS analysis of substrate and product. Dha residues are indicated by lower-case c. Unmodified Cys residues are indicated by an upper-case C. The Interactive Peptide Annotator tool was used to make panel D).[4]

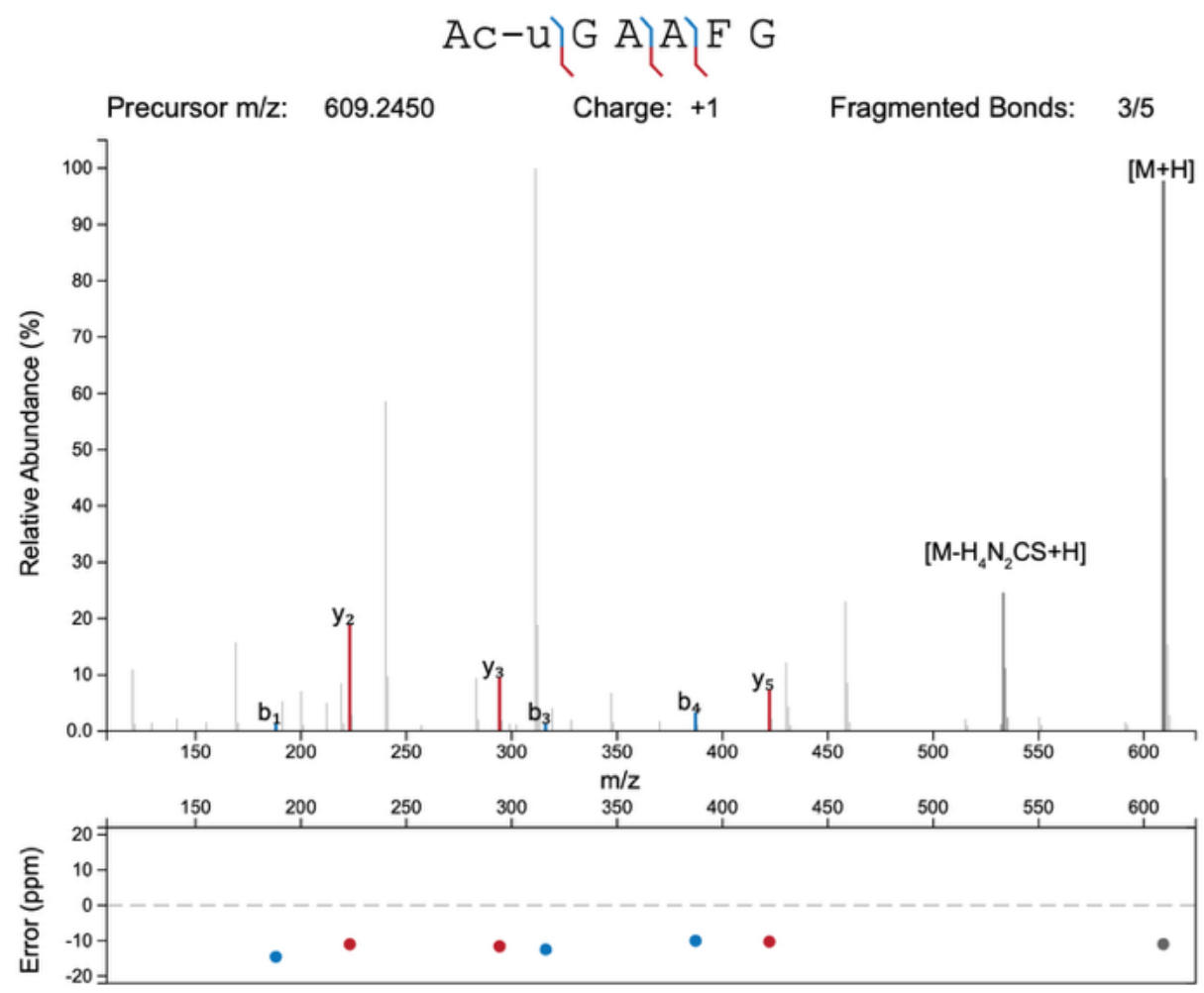

13

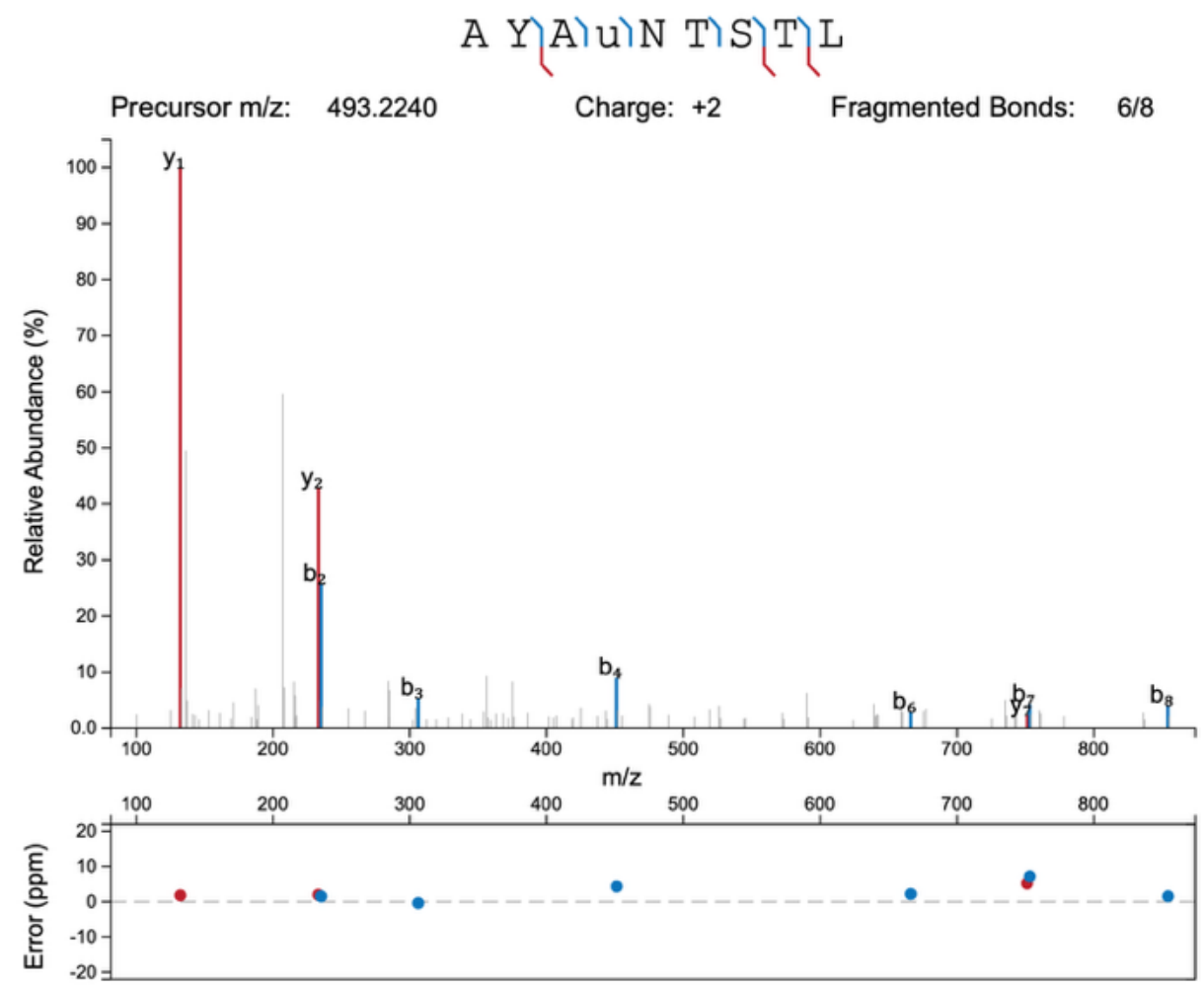

18

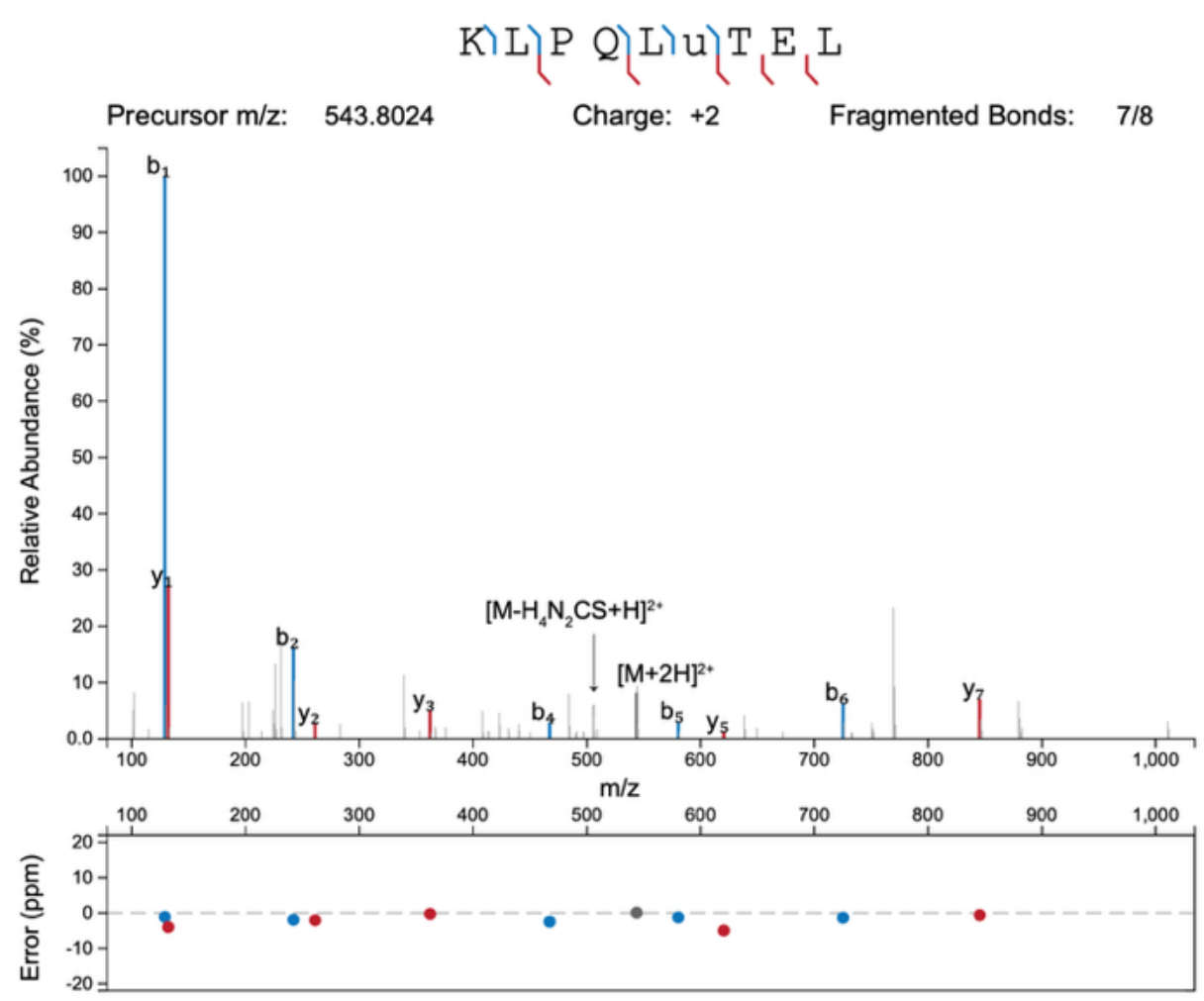

19

Figure S15. High resolution MS-MS analysis of peptide isothiourea intermediates **13**, **18**, and **19**. Cys(isothiourea) residues are indicated by lower-case u, and Dha residues are indicated by lower-case C. The Interactive Peptide Annotator tool was used.[4]

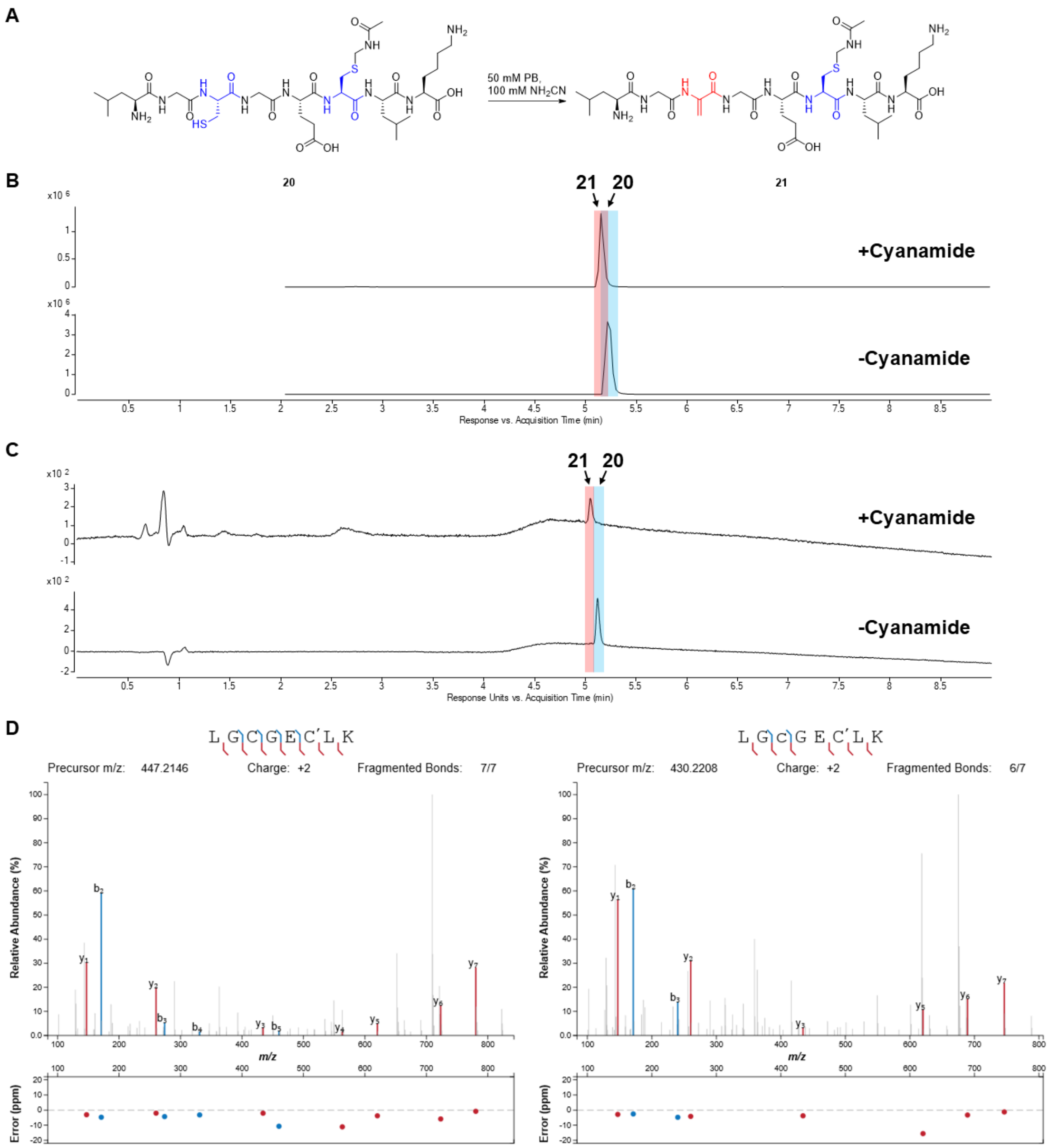

Figure S16. Conversion of **20** to **21**. A) Reaction scheme for the conversion of **20** to **21**. B) and C) Solutions of **20** analyzed by LCMS before and after treatment with cyanamide. B) Extracted ion-chromatograms extracting the following masses: 893.4219 (**20**) and 859.4352 (**21**). C) LC traces collected using UV-vis detection. The wavelength was set to 220 nm. For B) and C) the samples were not purified by HPLC prior to analysis. D) High resolution MS-MS analysis of substrate and product. Dha residues are indicated by lower-case c. Unmodified Cys residues are indicated by an upper-case C. Cys residues protected by the Acm group are indicated by an upper-case C prime. The Interactive Peptide Annotator tool was used to make panel D).[4]

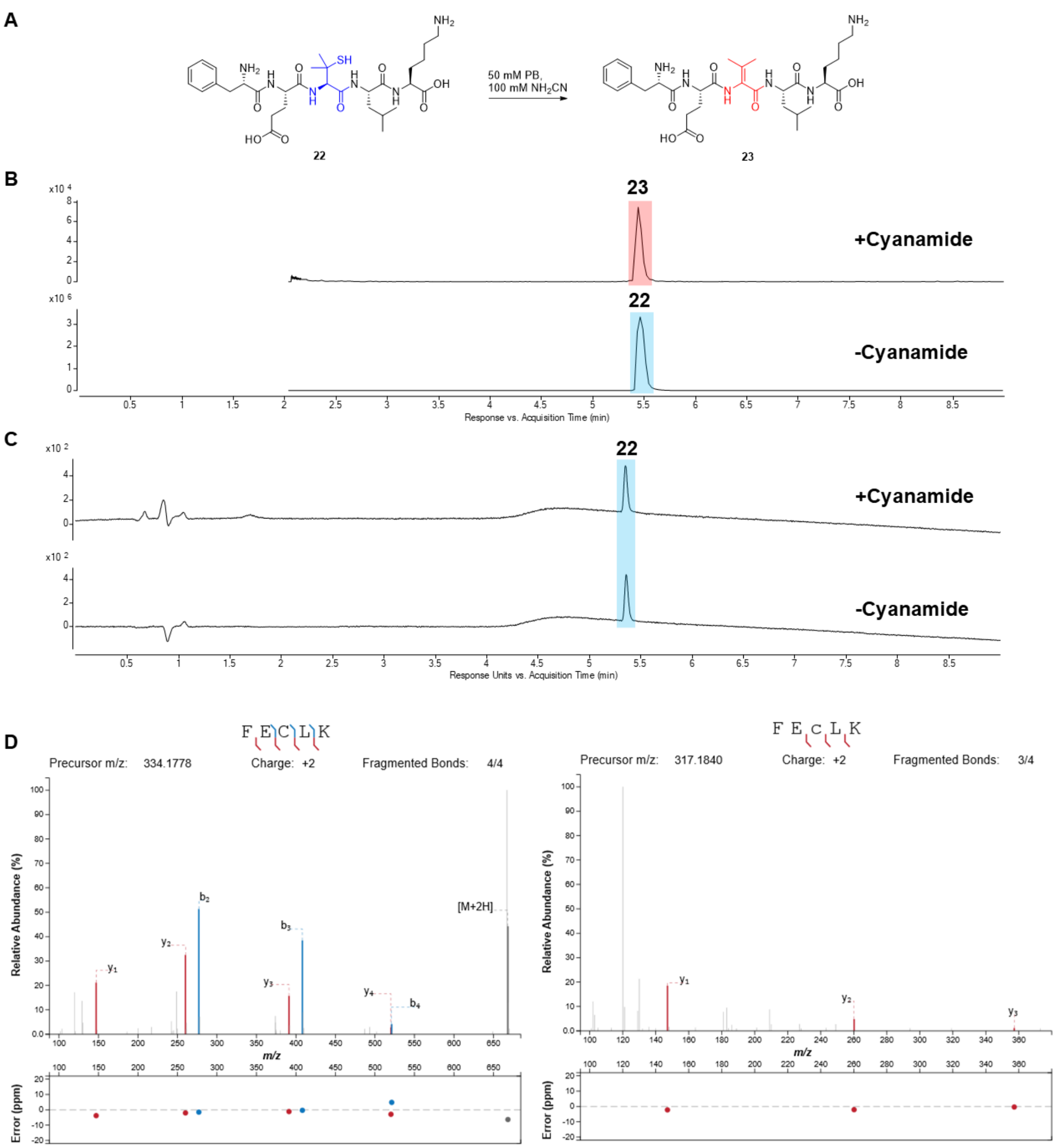

Figure S17. Attempted conversion of **22** to **23**. A) Reaction scheme for the conversion of **22** to **23**. B) and C) Solutions of **22** analyzed by LCMS before and after treatment with cyanamide. B) Extracted ion-chromatograms extracting the following masses: 667.3484 (**22**) and 633.3606 (**23**). C) LC traces collected using UV-vis detection. The wavelength was set to 220 nm. For B) and C) the samples were not purified by HPLC prior to analysis. D) High resolution MS-MS analysis of substrate and product. Dehydrovaline residues are indicated by lower-case c. Penicillamine residues are indicated by an upper-case C. The Interactive Peptide Annotator tool was used to make panel D).[4]

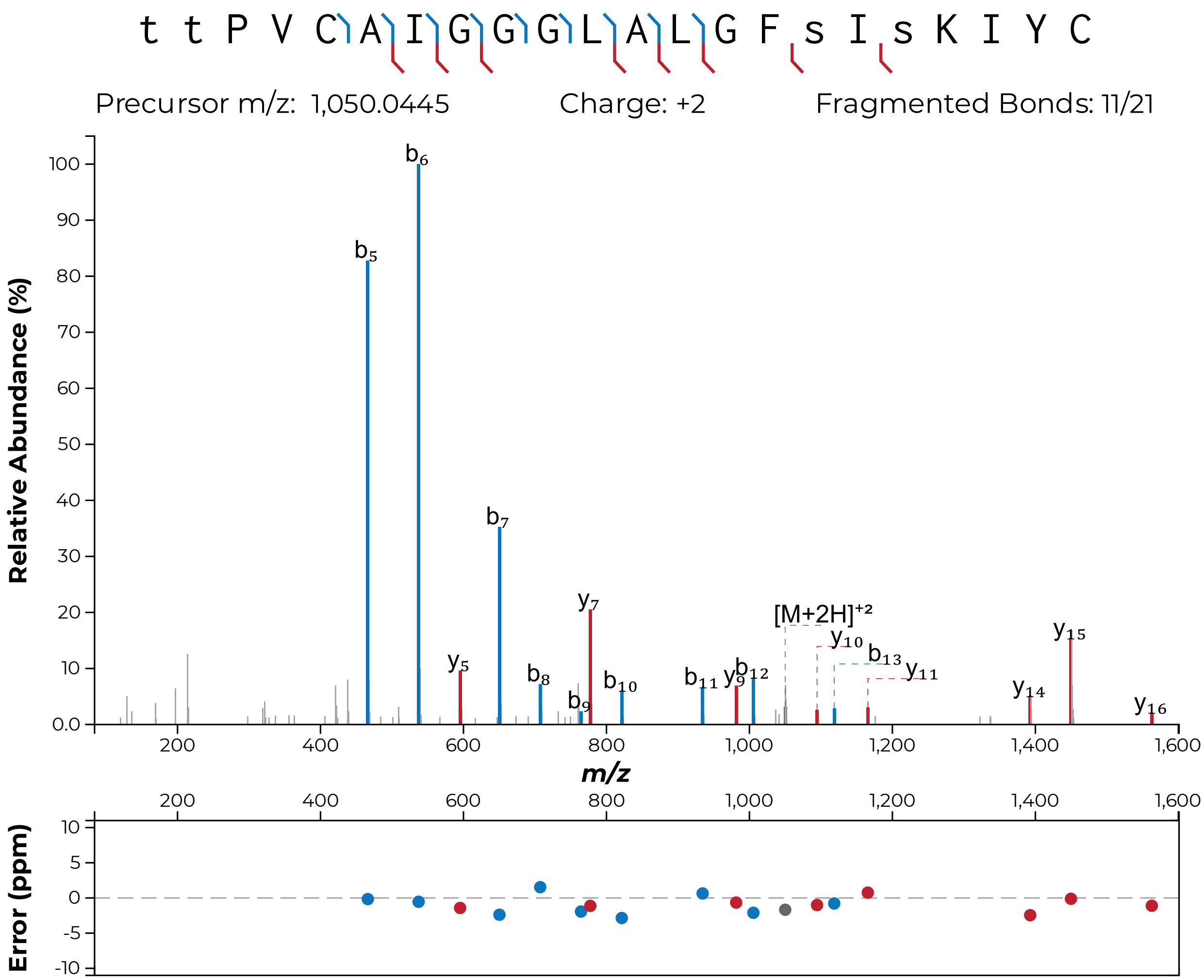

Figure S18. MS-MS analysis of Bib1β$_{Dha}$. The fragmentation data was analyzed using the Interactive Peptide Spectral Annotator (IPSA),[4] which generates predicted peptide fragments based on user-entered modifications. Lower case t was entered as Thr – 18 Da, and lowercase s was entered as Ser – 18 Da.

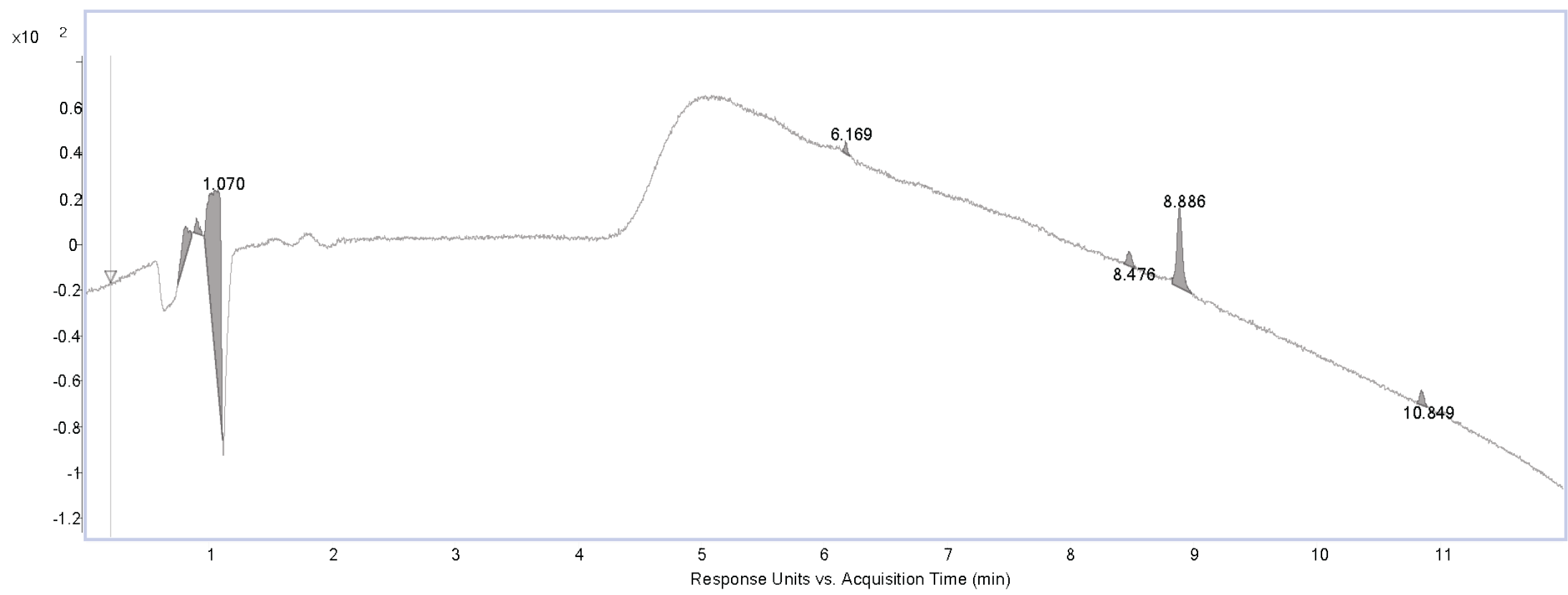

Figure S19. UPLC trace of Bib1β_{Dha} analyzed by UV-vis at 205 nm. The peak with a retention time of 8.886 min corresponds to Bib1β_{Dha}.

References

(1) Engelhardt, D. B.; Donnelly, B. L.; Beadle, J.; van Belkum, M. J.; Vederas, J. C. Ring-opening reactions for the solid-phase synthesis of nisin lipopeptide analogues. *Org. Biomol. Chem.* **2022**, *20* (45), 8988-8999.

(2) Todorovic, M.; Rivollier, P.; Wong, A. A. W. L.; Wang, Z.; Pryyma, A.; Nguyen, T. T.; Newell, K. C.; Froelich, J.; Perrin, D. M. Rationally Designed Amanitins Achieve Enhanced Cytotoxicity. *J. Med. Chem.* **2022**, *65* (15), 10357-10376.

(3) Mukherjee, S.; Huo, L.; Thibodeaux, G. N.; van der Donk, W. A. Synthesis and bioactivity of diastereomers of the virulence lanthipeptide cytolysin. *Org. Lett.* **2016**, *18*, 6188-6191.

(4) Brademan, D. R.; Riley, N. M.; Kwiecien, N. W.; Coon, J. J. Interactive peptide spectral annotator: A versatile web-based tool for proteomic applications. *Mol. Cell. Proteom.* **2019**, *18*, S193-S201.

(5) Moreira, R.; Yang, Y.; Luo, Y.; Gilmore, M. S.; van der Donk, W. A. Bibacillin 1: a two-component lantibiotic from *Bacillus thuringiensis*. *RSC Chem. Biol.* **2024**, *5*, 1060–1073.

(6) Moreira, R.; Chakraborty, B.; Yang, Y.; Padhi, C.; Gilmore, M. S.; Nair, S. K.; van der Donk, W. A. Combatting virulent gut bacteria by inhibiting the biosynthesis of a two-component lanthipeptide toxin. *Nat. Commun.* **2025**, *16*, 6936.

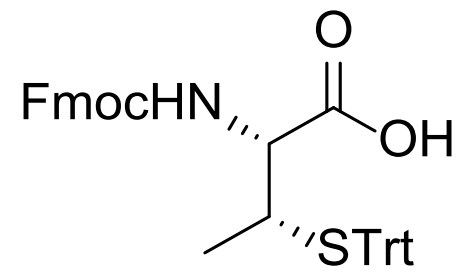

^{1}H NMR (500 MHz, CD_2Cl_2) spectrum of **S2**

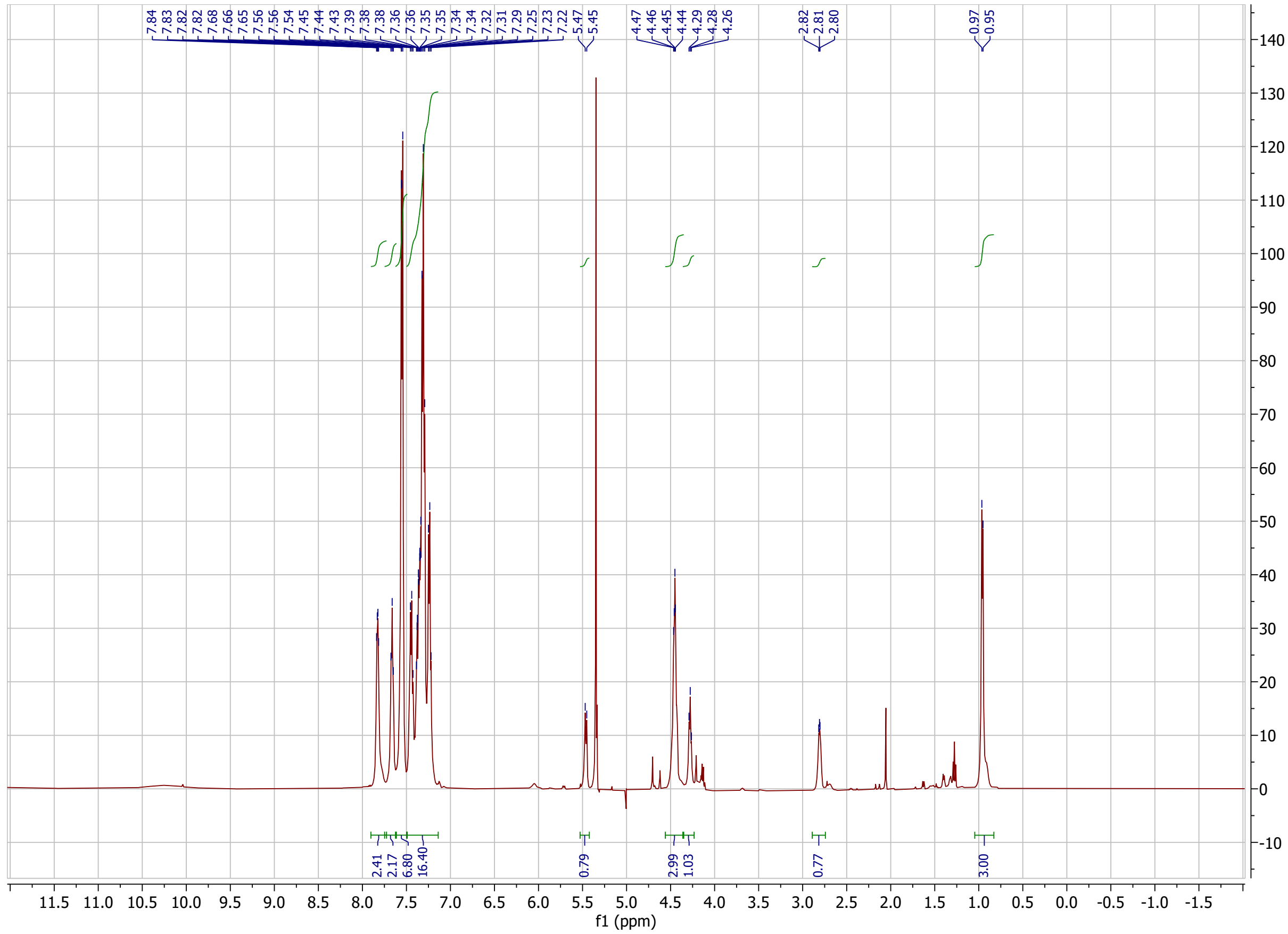

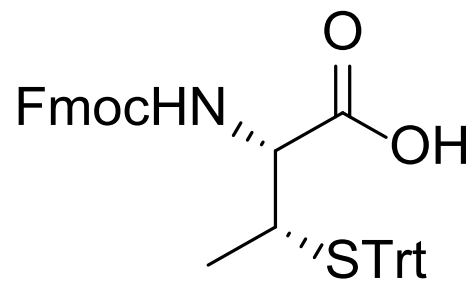

^{13}C NMR (125 MHz, CD_2Cl_2) spectrum of **S2**

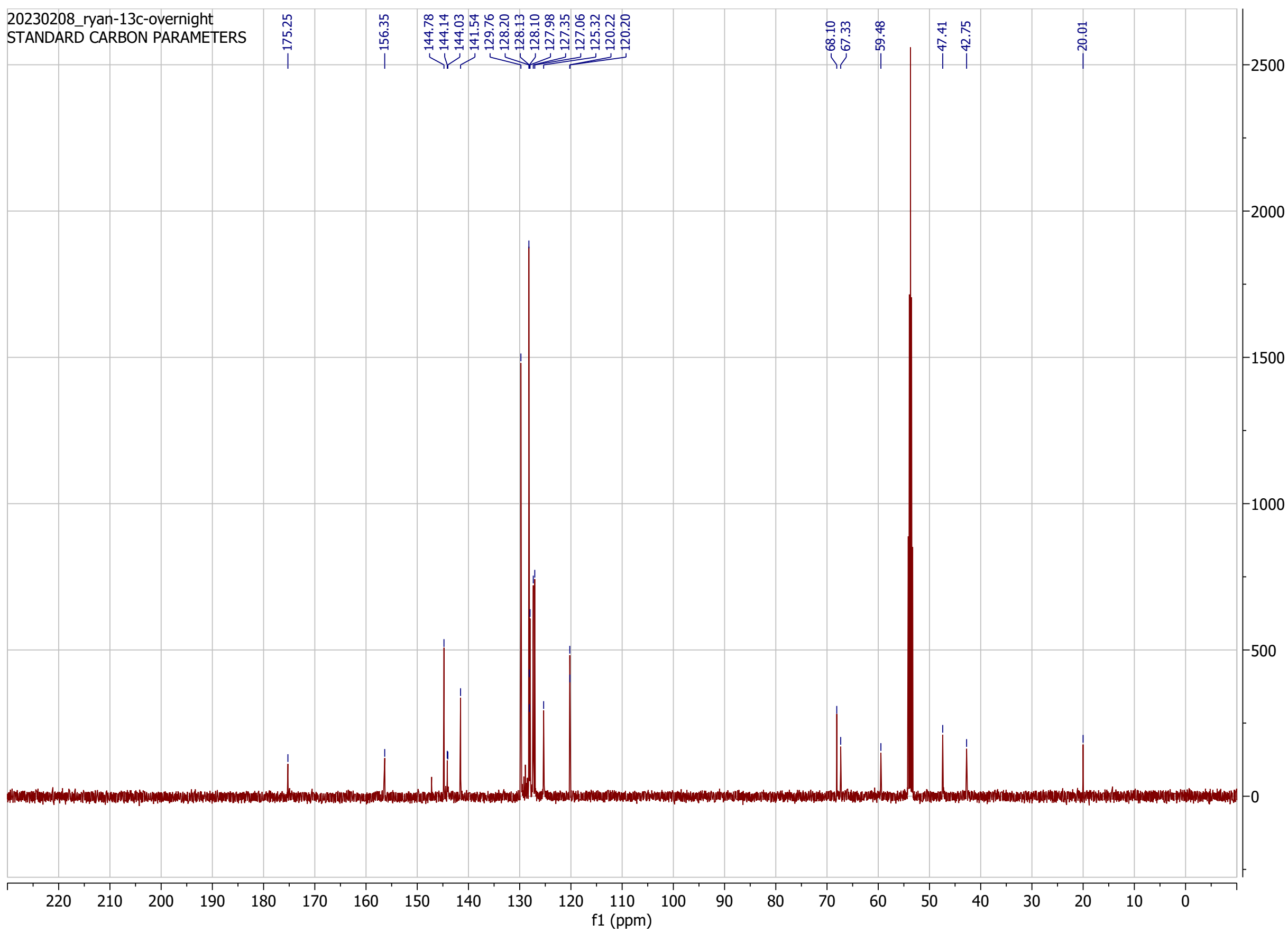

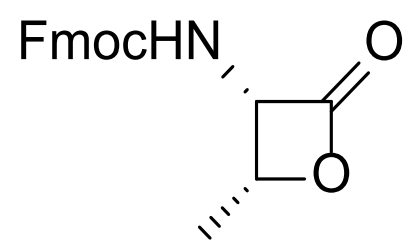

^{1}H NMR (500 MHz, $CDCl_3$) spectrum of **S4**

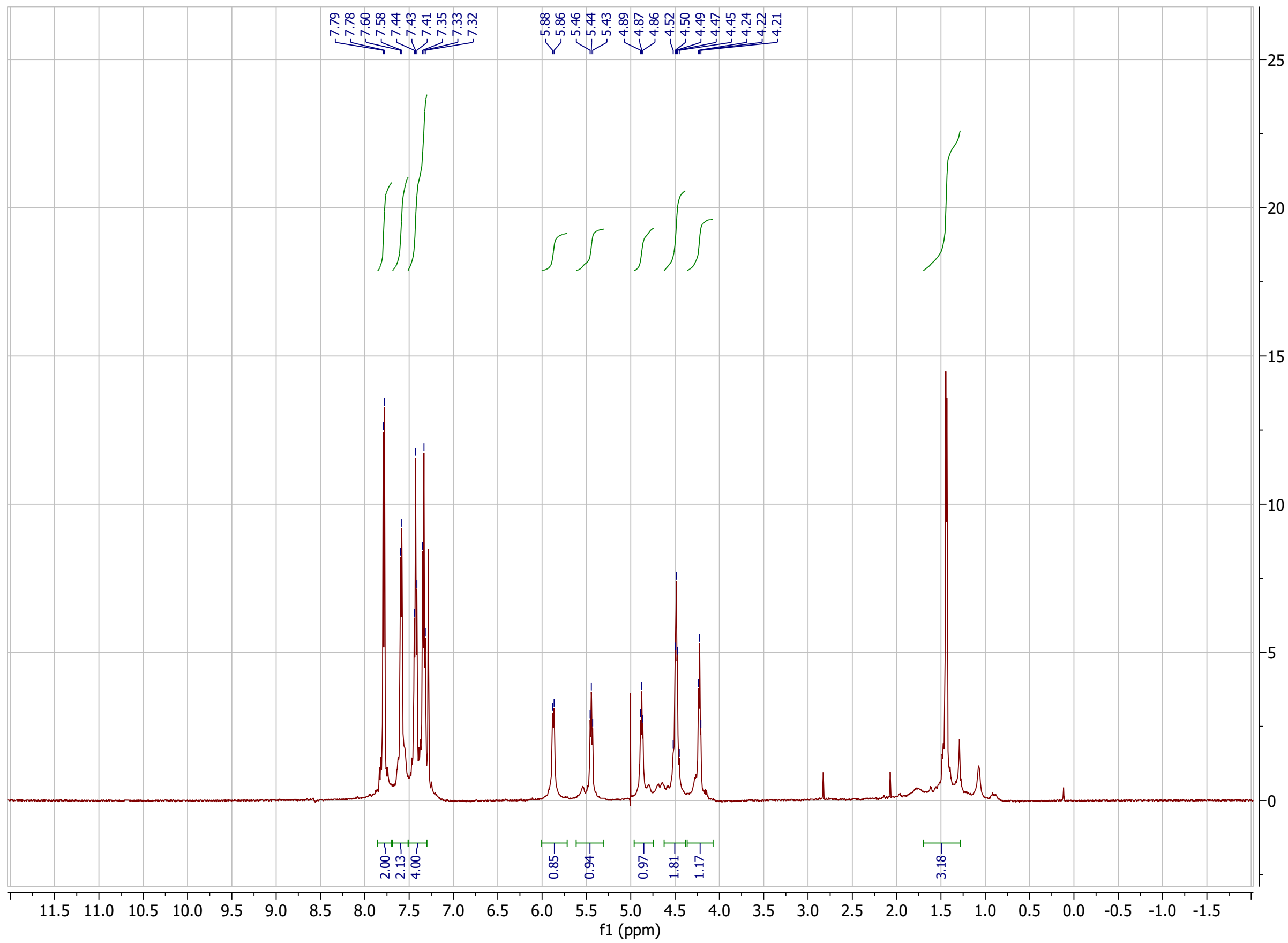

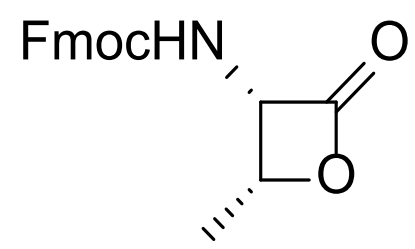

^{13}C NMR (75 MHz, CD_2Cl_2) spectrum of **S4**

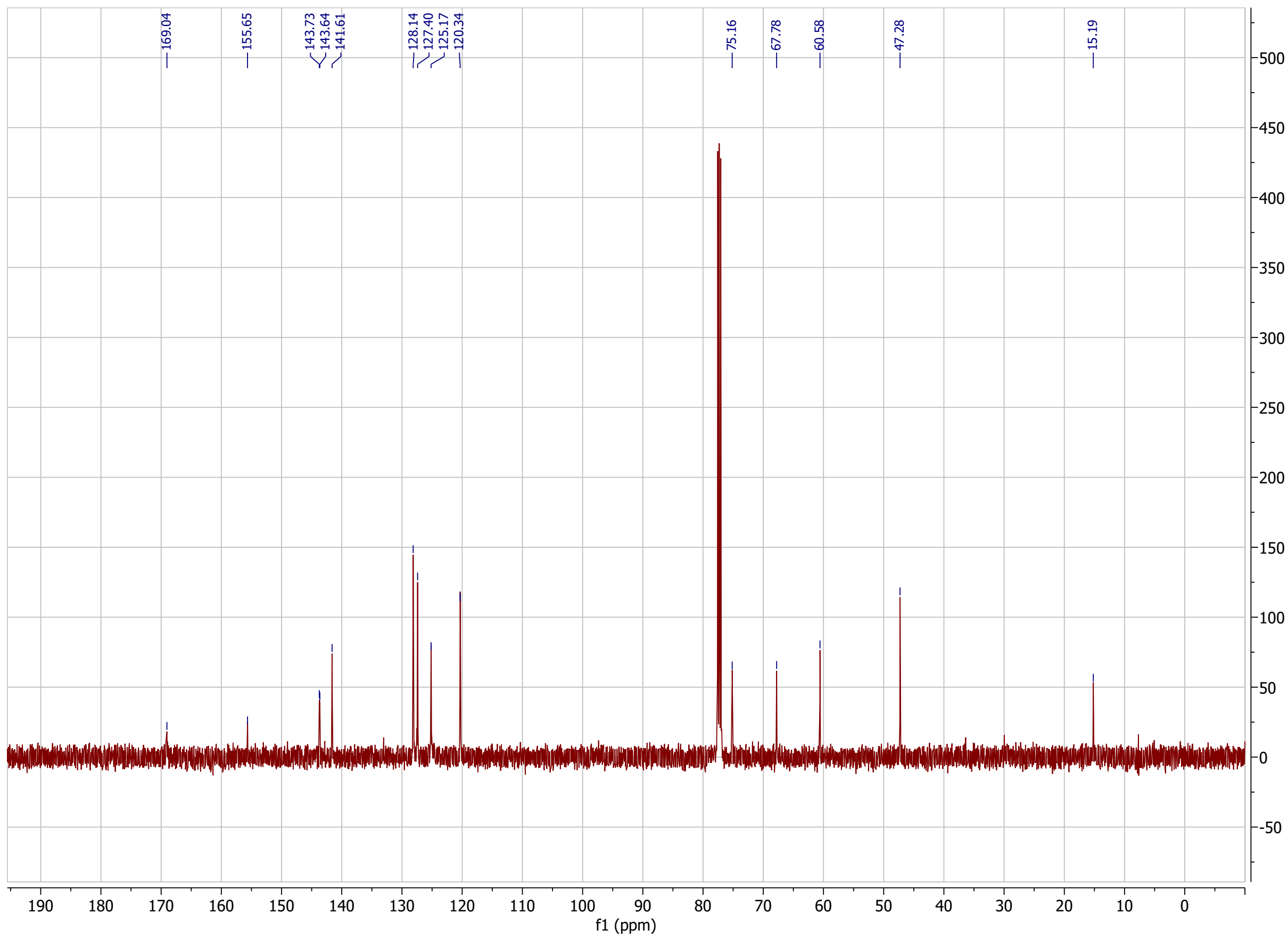

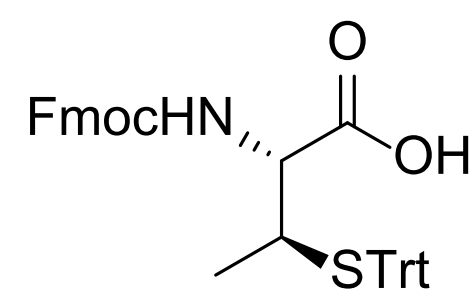

^{1}H NMR (500 MHz, CD_2Cl_2) spectrum of **S5**

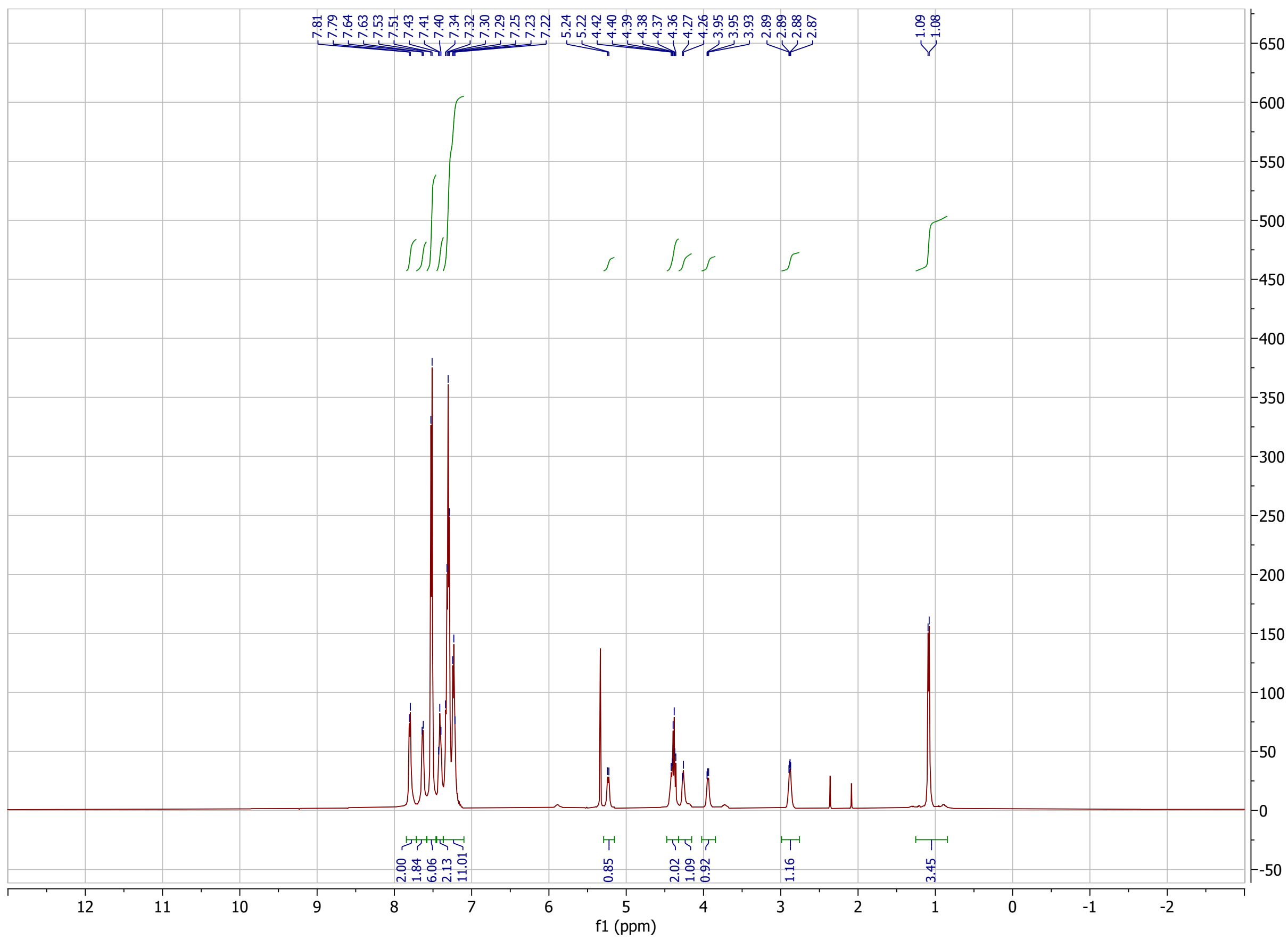

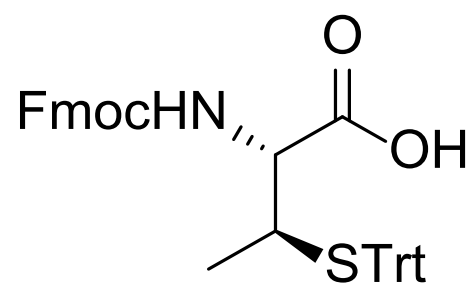

^{13}C NMR (125 MHz, $CDCl_3$) spectrum of **S5**

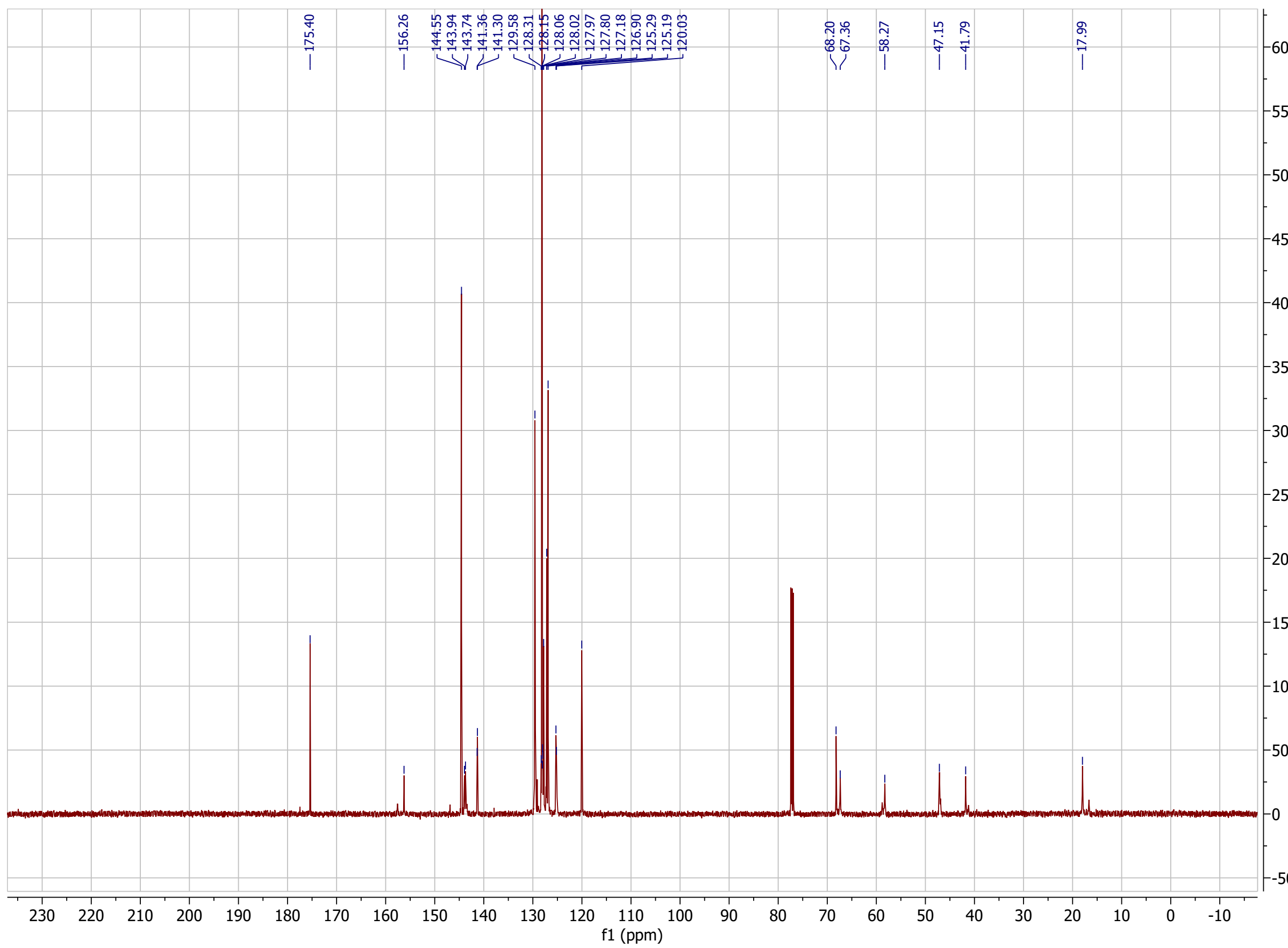

Supplement: Supplementary file 1 [file NIHMS2207430-supplement-SI.pdf]
